# Comparative study of CO_2_ insertion into pincer supported palladium alkyl and aryl complexes†

**DOI:** 10.1039/d3sc01459b

**Published:** 2023-07-12

**Authors:** Anthony P. Deziel, Sahil Gahlawat, Nilay Hazari, Kathrin H. Hopmann, Brandon Q. Mercado

**Affiliations:** a Department of Chemistry, Yale University P. O. Box 208107 New Haven Connecticut 06520 USA nilay.hazari@yale.edu; b Department of Chemistry, UiT The Arctic University of Norway N-9307 Tromsø Norway kathrin.hopmann@uit.no; c Hylleraas Center for Quantum Molecular Sciences, UiT The Arctic University of Norway 9037 Tromsø Norway

## Abstract

The insertion of CO_2_ into metal alkyl bonds is a crucial elementary step in transition metal-catalyzed processes for CO_2_ utilization. Here, we synthesize pincer-supported palladium complexes of the type (^*t*Bu^PBP)Pd(alkyl) (^*t*Bu^PBP = B(NCH_2_P^*t*^Bu_2_)_2_C_6_H_4_^−^; alkyl = CH_2_CH_3_, CH_2_CH_2_CH_3,_ CH_2_C_6_H_5_, and CH_2_-4-OMe-C_6_H_4_) and (^*t*Bu^PBP)Pd(C_6_H_5_) and compare the rates of CO_2_ insertion into the palladium alkyl bonds to form metal carboxylate complexes. Although, the rate constant for CO_2_ insertion into (^*t*Bu^PBP)Pd(CH_2_CH_3_) is more than double the rate constant we previously measured for insertion into the palladium methyl complex (^*t*Bu^PBP)Pd(CH_3_), insertion into (^*t*Bu^PBP)Pd(CH_2_CH_2_CH_3_) occurs approximately one order of magnitude slower than (^*t*Bu^PBP)Pd(CH_3_). CO_2_ insertion into the benzyl complexes (^*t*Bu^PBP)Pd(CH_2_C_6_H_5_) and (^*t*Bu^PBP)Pd(CH_2_-4-OMe-C_6_H_4_) is significantly slower than any of the n-alkyl complexes, and CO_2_ does not insert into the palladium phenyl bond of (^*t*Bu^PBP)Pd(C_6_H_5_). While (^*t*Bu^PBP)Pd(CH_2_CH_3_) and (^*t*Bu^PBP)Pd(CH_2_CH_2_CH_3_) are resistant to β-hydride elimination, we were unable to synthesize complexes with *n*-butyl, iso-propyl, and *tert*-butyl ligands due to β-hydride elimination and an unusual reductive coupling, which involves the formation of new C–B bonds. This reductive process also occurred for (^*t*Bu^PBP)Pd(CH_2_C_6_H_5_) at elevated temperature and a related process involving the formation of a new H–B bond prevented the isolation of (^*t*Bu^PBP)PdH. DFT calculations provide insight into the relative rates of CO_2_ insertion and indicate that steric factors are critical. Overall, this work is one of the first comparative studies of the rates of CO_2_ insertion into different metal alkyl bonds and provides fundamental information that may be important for the development of new catalysts for CO_2_ utilization.

## Abstract 2

The synthesis of a range of PBP supported palladium pincer complexes with different alkyl ligands is described. The rates of CO_2_ insertion into the alkyl group are quantified and rationalized based on the identity of the alkyl ligand.

## Graphical abstract

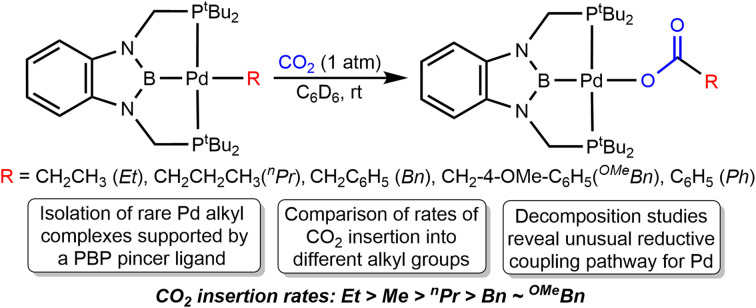

## Introduction

There is considerable interest in the use of carbon dioxide (CO_2_) as a carbon containing chemical feedstock due to its low cost, non-toxic nature, and abundance.^1^ However, only a small number of chemicals are currently industrially produced from CO_2_.^1*g*^ This is in part because the kinetic barriers associated with bond forming processes involving CO_2_ are often prohibitively high. Transition metal catalysts represent a promising method to increase the range of products generated from CO_2_ because they can create lower energy pathways for activating and functionalizing CO_2_.^1^ To date, most transition metal catalysts for CO_2_ utilization have converted CO_2_ into other C_1_ products such as methane, CO, formic acid, and methanol and there are limited examples of catalysts that can form products containing C–C bonds, such as fuels, from CO_2_.^1*g*^ As a result, the formation of products containing a C–C bond from CO_2_ has been identified as a high priority research area by the United States National Academies of Science.^2^1
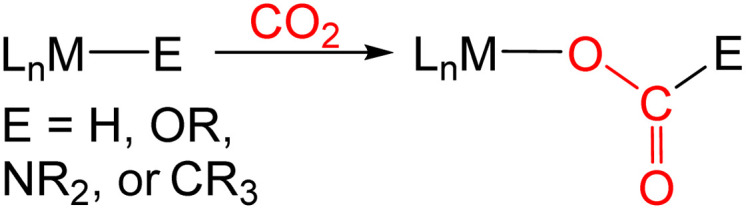


In many transition metal catalyzed processes for CO_2_ utilization, the insertion of CO_2_ into a metal–E σ-bond (for example E = H, OR, NR_2_, or CR_3_) is a crucial elementary step (eqn (1)).^3^ This is especially the case for late transition metals, where the relative weakness of the M–O bonds makes subsequent cleavage of the M–O bond more facile.^3^ The insertion of CO_2_ into a metal alkyl bond is a particularly important reaction because it can ultimately result in the generation of products containing a C–C bond. For example, Group 10 catalysts have been used for the formation of carboxylic acids through the carboxylation of a variety of alkyl halides and pseudo halides.^4^ In these reactions, C–C bonds are proposed to form between CO_2_ and the alkyl electrophile *via* the insertion of CO_2_ into a metal alkyl bond. However, at this stage there is limited experimental information on the pathways for CO_2_ insertion into metal alkyl bonds, as most studies have primarily involved isolated examples with a single metal complex,^5–14^ and thus, it is unclear how changing the nature of the alkyl group or ancillary ligand impacts the reaction. Further, kinetic studies are relatively rare,^6,8*a*,*b*,9*d*,11*c*,13*g*,14*b*^ which means that computational results cannot be benchmarked against experimental data.

**Fig. 1:**
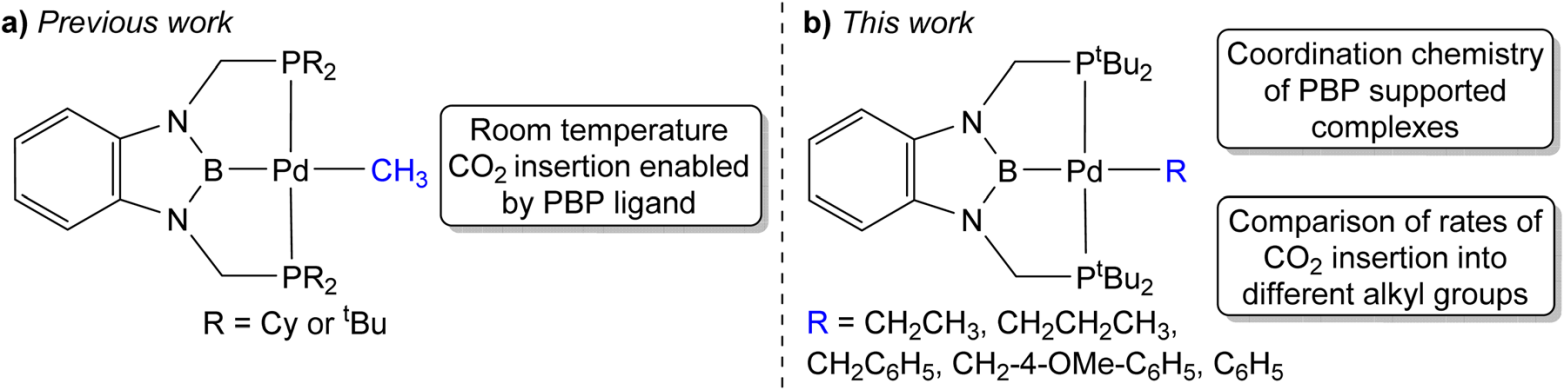
(a) Previous example of CO_2_ insertion into ^R^PBP supported palladium methyl complexes at room temperature. (b) ^*t*Bu^PBP supported palladium complexes studied in this work, which reveal fundamental information about the coordination chemistry of the ^*t*Bu^PBP ligand and enable a comparison between the rates of CO_2_ insertion as a function of the alkyl ligand.

Previous kinetic studies exploring CO_2_ insertion into well-defined metal alkyl complexes have almost exclusively focused on metal methyl species.^6,8*a*,*b*,9*d*,11*c*,13*g*,14*b*^ This is because of the stability of metal methyl complexes, which in contrast to longer chain alkyl containing complexes, such as metal ethyl complexes, do not undergo β-hydride elimination. A major limitation in studying CO_2_ insertion into metal methyl bonds, and in particular the types of Group 10 metal alkyl complexes that are relevant to catalysis, is the paucity of systems that are stable and react under mild reaction conditions. Most systems require high temperatures and do not give quantitative yields of products, which prevents kinetic studies. We recently described the insertion of CO_2_ into palladium and nickel methyl complexes supported by ^R^PBP (^R^PBP = B(NCH_2_PR_2_)_2_C_6_H_4_^−^; R = Cy or ^*t*^Bu) pincer ligands (Fig. 1a).^13*g*^ The strong *trans*-influence of the boryl donor in the pincer ligand destabilizes the methyl group and as a consequence these complexes insert CO_2_ at room temperature, which enabled us to perform detailed kinetic studies on CO_2_ insertion into a metal methyl bond.

We hypothesized that the ^R^PBP framework may stabilize palladium complexes with other alkyl ligands, as pincer ligands are known to inhibit β-hydride elimination from square planar palladium(ii) complexes.^15^ Further, given that the ^R^PBP ligand can facilitate CO_2_ insertion reactions under mild conditions,^13*g*^ we postulated that the synthesis of a family of ^R^PBP supported palladium alkyl complexes would enable us to perform a rare experimental comparison of the rates of CO_2_ insertion as the alkyl ligand is varied. In this work, we describe the synthesis of a series of ^*t*Bu^PBP supported palladium complexes with ethyl, n-propyl, benzyl, and phenyl ligands. Although (^*t*Bu^PBP)Pd(CH_2_CH_3_) (1-Et), (^*t*Bu^PBP)Pd(CH_2_CH_2_CH_3_) (1-*^n^*Pr), (^*t*Bu^PBP)PdCH_2_C_6_H_5_ (1-Bn), and (^*t*Bu^PBP)PdCH_2_-4-OMe-C_6_H_4_ (1-^OMe^Bn), are sufficiently stable in solution to be isolated, attempts to synthesize complexes with *n*-butyl, iso-propyl, and *tert*-butyl ligands were unsuccessful due to rapid decomposition *via* either β-hydride elimination or an unusual reductive pathway that generates a new C–B bond. A similar reductive process to form a new H–B bond occurs in the putative hydride complex (^*t*Bu^PBP)PdH. The stability of 1-Et, 1-*^n^*Pr, 1-Bn, and 1-^OMe^Bn allowed us to determine the rates of CO_2_ insertion into the palladium alkyl bonds. The rate constant for CO_2_ insertion into 1-Et is *over double* the rate constant previously measured for CO_2_ insertion into (^*t*Bu^PBP)Pd(CH_3_) (1-Me),^13*g*^ while insertion into 1-*^n^*Pr occurs at *approximately one-tenth* the rate of 1-Me. This is a remarkable difference given the relatively minor changes in the alkyl ligand. The insertion of CO_2_ into the benzyl complexes, 1-Bn and 1-^OMe^Bn, is significantly slower than any of n-alkyl complexes and CO_2_ does not insert into the palladium phenyl bond of the related complex (^*t*Bu^PBP)Pd(C_6_H_5_) (1-Ph). DFT calculations enabled the rationalization of the relative rates of CO_2_ insertion and suggest that steric factors are the predominant reason for the differences in the rates of insertion between 1-Me, 1-Et, and 1-*^n^*Pr. Overall, the fundamental insight on CO_2_ insertion provided in this work will likely assist in the development of improved and new catalysts for CO_2_ utilization.

## Results and discussion

### Synthesis of ^*t*Bu^PBP supported palladium alkyl and aryl complexes

**Fig. 2:**
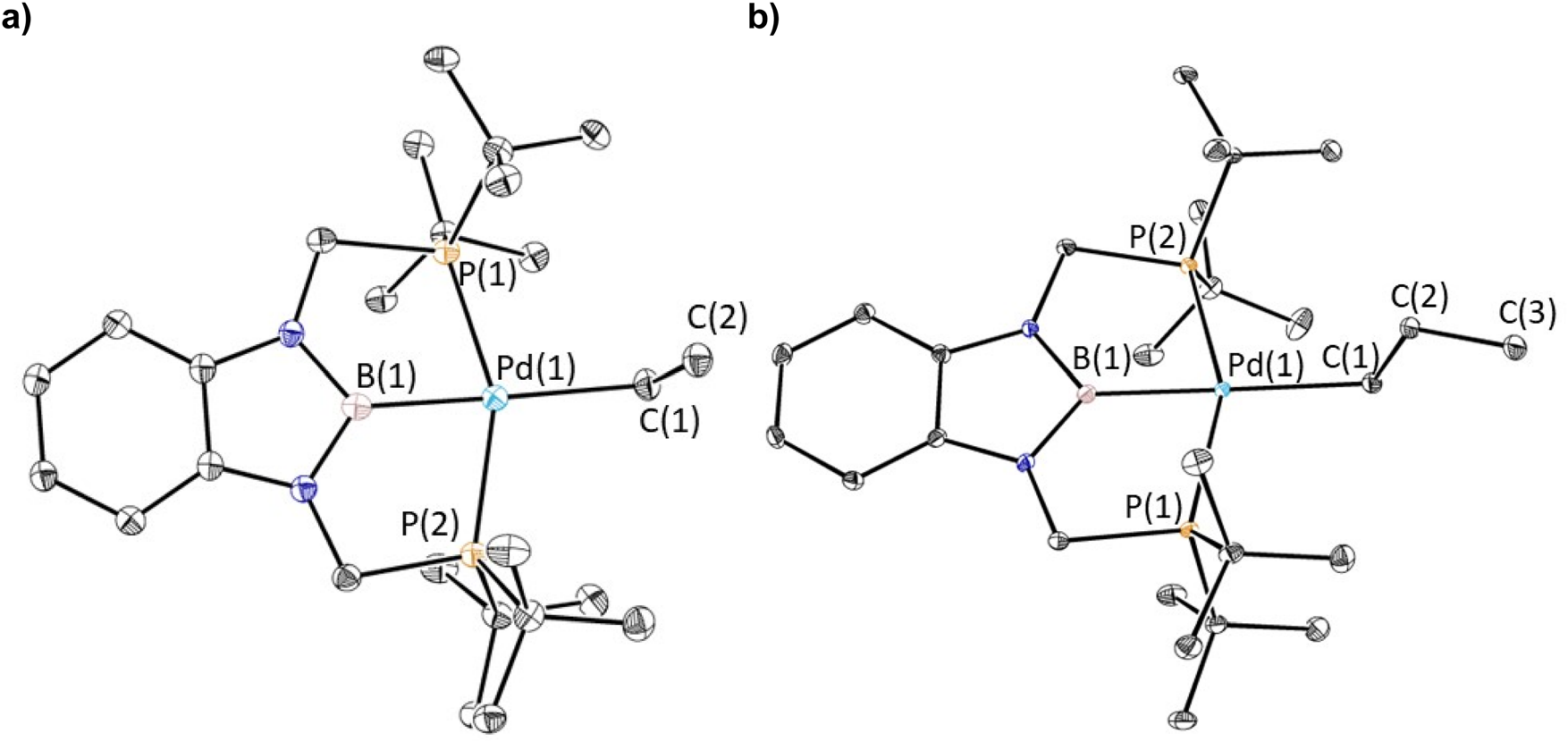
(a) Solid-state structure of 1-Et with thermal ellipsoids at 30% probability. Hydrogen atoms are omitted for clarity. Selected distances (Å) and angles (°): Pd(1)–B(1) 2.029(7), Pd(1)–C(1) 2.226(6), Pd(1)–P(1) 2.3184(15), Pd(1)–P(2) 2.3189(14), C(1)–C(2) 1.506(9), B(1)–Pd(1)–C(1) 175.3(2), B(1)–Pd(1)–P(1) 76.39(19), B(1)–Pd(1)–P(2) 78.23(19), C(1)–Pd(1)–P(1) 103.15(18), C(1)–Pd(1)–P(2) 102.61(18), P(1)–Pd(1)–P(2) 153.99(6), Pd(1)–C(1)–C(2) 115.8(4). (b) Solid-state structure of 1-*^n^*Pr with thermal ellipsoids at 30% probability. Hydrogen atoms are omitted for clarity. Selected distances (Å) and angles (°): Pd(1)–B(1) 2.020(3), Pd(1)–C(1) 2.209(2), Pd(1)–P(1) 2.3143(5), Pd(1)–P(2) 2.3135(5), C(1)–C(2) 1.531(3), C(2)–C(3) 1.528(3), P(1)–Pd(1)–P(2) 154.23(2), C(1)–Pd(1)–P(1) 101.54(6), C(1)–Pd(1)–P(2) 103.95(6), B(1)–Pd(1)–P(1) 77.67(7), B(1)–Pd(1)–P(2) 77.07(7), B(1)–Pd(1)–C(1) 177.01(9), Pd(1)–C(1)–C(2) 114.49(15).

Previous studies have demonstrated that reactions of complexes of the form (^R^PBP)PdCl (R = ^i^Pr or ^*t*^Bu) with MeLi generate stable palladium methyl complexes.^13*g*^ In an analogous fashion, treatment of (^*t*Bu^PBP)PdCl (1-Cl) with EtLi or ^*n*^PrMgCl in benzene results in the formation of (^*t*Bu^PBP)Pd(CH_2_CH_3_) (1-Et) and (^*t*Bu^PBP)Pd(CH_2_CH_2_CH_3_) (1-*^n^*Pr), which were isolated in yields of 76 and 59%, respectively, after recrystallization (eqn (2)).^16^1-Et is a rare example of an isolated pincer supported palladium ethyl complex,^15,17^ while 1-*^n^*Pr is to the best of our knowledge only the second example of an isolated palladium propyl complex.^18^1-Et and 1-*^n^*Pr are indefinitely stable at room temperature in benzene and β-hydride elimination to generate ethylene or propene and a putative palladium hydride (*vide infra*) only occurs at temperatures greater than 60 °C. Recrystallization from pentane generated crystals of 1-Et and 1-*^n^*Pr suitable for X-ray diffraction (Fig. 2a and b). In both cases, the geometry around Pd is distorted square planar and the P(1)–Pd(1)–P(2) bond angles are 153.99(6) and 154.23(2)° in 1-Et and 1-*^n^*Pr, respectively, indicating that the phosphorus donors of the pincer ligand deviate significantly from linearity. The Pd–B bond distances are 2.029(7) Å in 1-Et and 2.020(3) Å in 1-*^n^*Pr, which are significantly longer than the Pd–B bond distance in (^*t*Bu^PBP)PdCl (Pd–B is 1.972(4) Å in 1-Cl).^19^ This is consistent with the ethyl or propyl ligand exerting a stronger *trans*-influence than a chloride ligand. Although, crystallographically characterized examples of palladium ethyl complexes are rare,^15,17,20^ the Pd–C bond distance in 1-Et is longer than those typically reported (Pd(1)–C(1) is 2.226(6) Å in 1-Et) and is most comparable to a PSiP-supported palladium ethyl complex, which also contains a strong *trans*-influence donor (silyl) opposite the palladium.^15^ In 1-*^n^*Pr the Pd(1)–C(1) is 2.209(2) Å, which is within error of the Pd–C bond distance in 1-Et. The carbon atom bound to palladium (C(1)) is distorted from tetrahedral in both 1-Et and 1-*^n^*Pr and Pd(1)–C(1)–C(2) angles of 115.8(4)° and 114.49(15)°, respectively, are observed. Analysis of the literature reveals that this is a general trend for Group 10 alkyl complexes.^21^ Overall, the geometrical parameters around palladium are similar in 1-Me,^13*g*^1-Et, and 1-*^n^*Pr, with the exception that the Pd–C bond distance in 1-Me is slightly shorter than in 1-Et or 1-*^n^*Pr (Pd(1)–C(1) is 2.185(4) Å in 1-Me), which is qualitatively consistent with DFT calculations (*vide infra*). This suggests that as expected the change from methyl to ethyl to *n*-propyl has little impact on the geometry around palladium.2
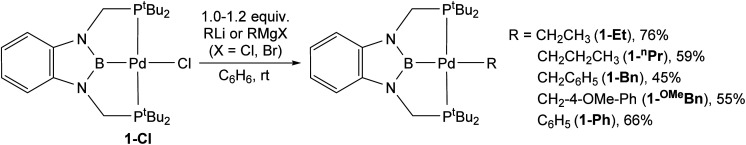


**Fig. 3:**
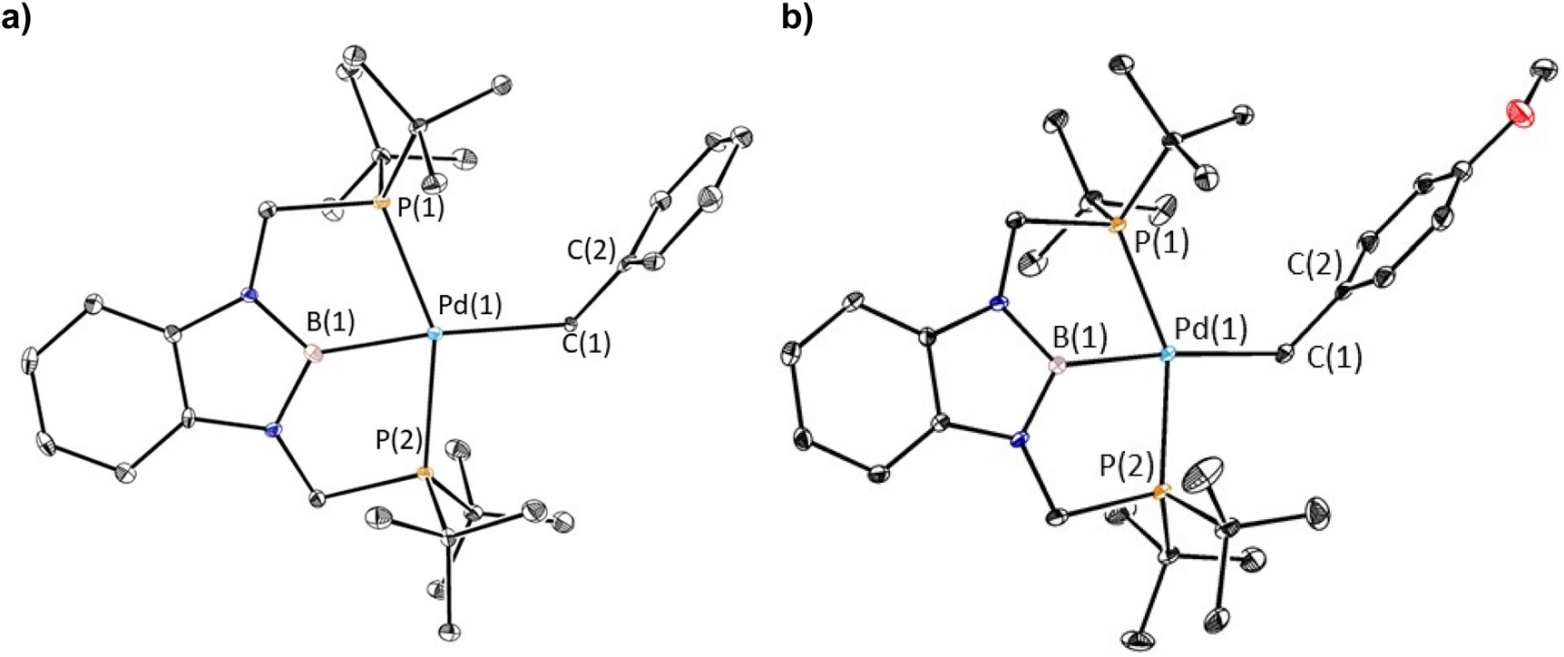
(a) Solid-state structure of 1-Bn with thermal ellipsoids at 30% probability. Hydrogen atoms are omitted for clarity. Selected distances (Å) and angles (°): Pd(1)–B(1) 2.032(4), Pd(1)–C(1) 2.260(3), Pd(1)–P(1) 2.3429(10), Pd(1)–P(2) 2.3407(10), C(1)–C(2) 1.477(5), B(1)–Pd(1)–C(1) 174.32(15), B(1)–Pd(1)–P(1) 76.60(12), B(1)–Pd(1)–P(2) 77.27(12), C(1)–Pd(1)–P(1) 108.72(9), C(1)–Pd(1)–P(2) 97.33(9), P(1)–Pd(1)–P(2) 153.77(3), Pd(1)–C(1)–C(2) 127.7(2). (b) Solid-state structure of 1-^OMe^Bn with thermal ellipsoids at 30% probability. Hydrogen atoms are omitted for clarity. Selected distances (Å) and angles (°): Pd(1)–B(1) 2.025(3), Pd(1)–C(1) 2.249(3), Pd(1)–P(1) 2.3360(7), Pd(1)–P(2) 2.3402(8), C(1)–C(2) 1.486(4), P(1)–Pd(1)–P(2) 154.50(3), C(1)–Pd(1)–P(1) 107.42(8), C(1)–Pd(1)–P(2) 97.83(7), B(1)–Pd(1)–P(1) 77.05(9), B(1)–Pd(1)–P(2) 77.53(9), B(1)–Pd(1)–C(1) 174.21(11), Pd(1)–C(1)–C(2) 123.44(18).

The reaction of 1-Cl with (CH_2_C_6_H_5_)MgCl or (4-OMe-CH_2_C_6_H_4_)MgCl resulted in the isolation of 1-Bn or 1-^OMe^Bn, which are rare examples of pincer supported benzyl complexes,^22^ in yields of 45% or 55%, respectively (eqn (2)). In both cases, it is important to remove the MgCl_2_ by-product from the Grignard reagent or the benzyl complexes slowly convert back to 1-Cl in solution. In fact, the relatively low yields of 1-Bn and 1-^OMe^Bn are in part due to the successive recrystallizations that are required to ensure MgCl_2_ impurities are not present. Both 1-Bn and 1-^OMe^Bn were characterized by X-ray crystallography (Fig. 3a and b). The solid-state structures indicate that the geometry around palladium is distorted square planar and the geometrical parameters associated with the binding of the ^*t*Bu^PBP ligand in 1-Bn and 1-^OMe^Bn are analogous to those in 1-Et and 1-*^n^*Pr. The long Pd–B bond distances (2.032(4) Å in 1-Bn and 2.025(3) Å in 1-^OMe^Bn) are consistent with the high *trans*-influence of the benzyl ligand. The benzyl ligand binds in an η^1^-fashion with Pd–C bond distances of 2.260(3) Å in 1-Bn and 2.249(3) Å in 1-^OMe^Bn, which are longer than almost all other palladium complexes that feature an η^1^-benzyl ligand.^22^ Further, the carbon atom bound to palladium (C(1)) is significantly distorted from tetrahedral, with Pd(1)–C(1)–C(2) angles of 127.7(2)° and 123.44(18)° observed for 1-Bn and 1-^OMe^Bn, respectively. Although this deviation from tetrahedral is typical for Group 10 benzyl complexes,^21*a*,*c*,23^ these are some of the largest angles reported perhaps due to the steric congestion around the palladium.

**Fig. 4:**
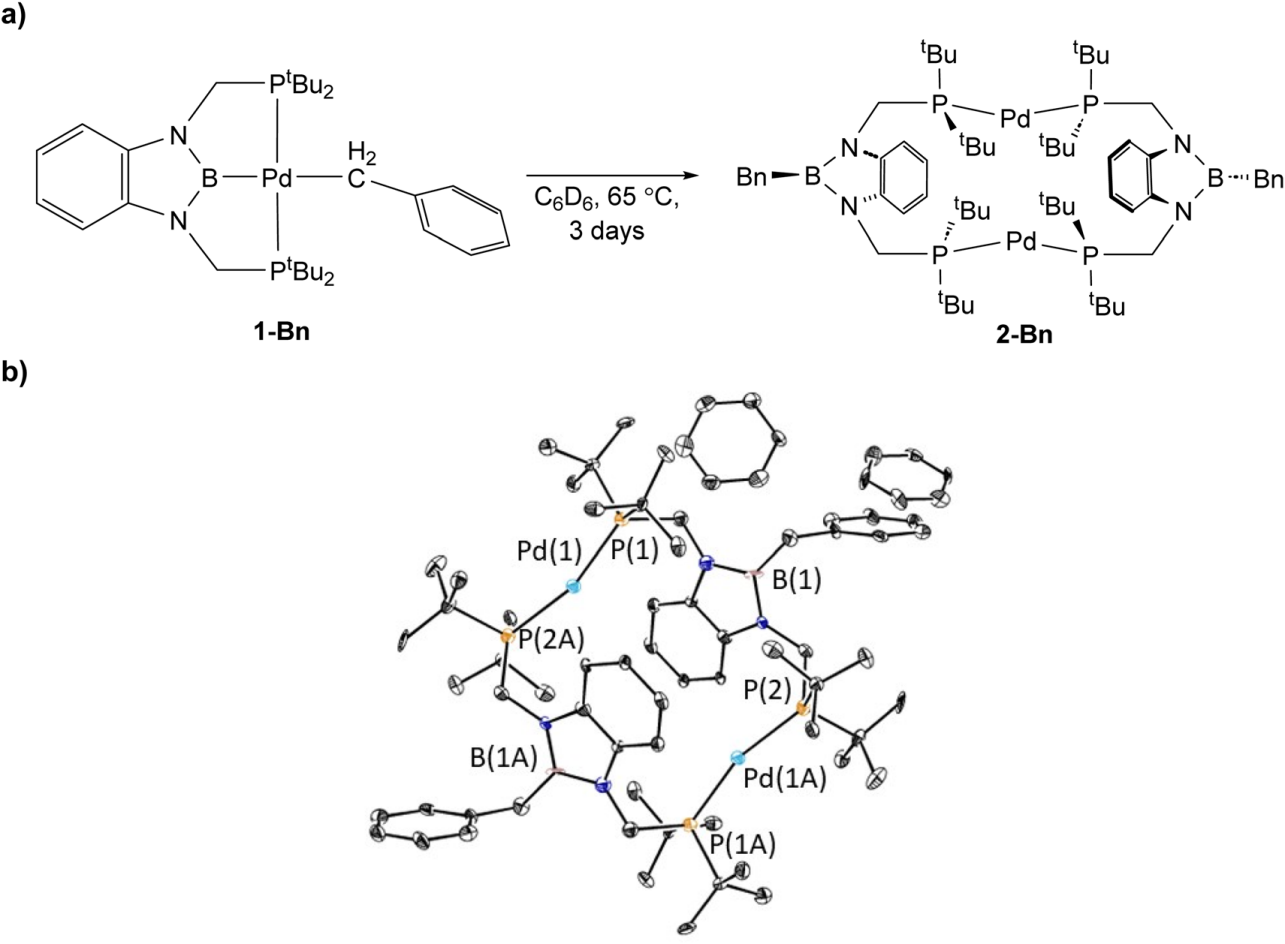
(a) Reaction scheme for decomposition of (^*t*Bu^PBP)Pd(CH_2_C_6_H_5_) (1-Bn) to (^*t*Bu^PB^Bn^P)_2_Pd_2_ (2-Bn) and (b) solid-state structure of 2-Bn with thermal ellipsoids at 30% probability. Hydrogen atoms are omitted for clarity. Selected distances (Å) and angles (°): Pd(1)–P(1) 2.277(2), Pd(1)–P(2A) 2.273(2), P(1)–Pd(1)–P(2A) 163.34(9).

1-Bn is stable when left in C_6_D_6_ at room temperature, but complete decomposition is observed when it is heated for 3 days at 65 °C, with the major product being a new dimeric complex, (^*t*Bu^PB^Bn^P)_2_Pd_2_ (2-Bn, Bn = benzyl) (Fig. 4a). Based on NMR spectroscopy we propose that 1-^OMe^Bn decomposes *via* a similar pathway (see ESI†). 2-Bn was characterized by X-ray crystallography (Fig. 4b). In 2-Bn, two new C–B bonds have formed presumably due to a reductive coupling reaction between the benzyl ligands and the boron atom of the ^*t*Bu^PBP ligands. This causes a reduction in the palladium center from palladium(ii) in 1-Bn to palladium(0) in 2-Bn. The boron atom of the pincer ligand no longer coordinates to the palladium center and the two phosphorus donors of the new bidentate ^*t*Bu^PB^Bn^P ligands do not coordinate to the same palladium center but instead coordinate to two different palladium atoms. The pathway for this ligand rearrangement is unclear. Consistent with the reduction in oxidation state, the geometry around the palladium centers in 2-Bn are distorted linear. We have previously observed a similar decomposition pathway for (^*t*Bu^PBP)Ni(CH_3_), which results in the formation of a nickel(0) dinitrogen complex,^13*g*^ but this is the first time the reductive decomposition pathway has been observed for palladium. We hypothesize that reductive coupling occurs more readily in 1-Bn compared to 1-Et or 1-Me (where it is not observed to any significant extent spectroscopically) because there is greater steric congestion in the case of the palladium benzyl complex. This is also in agreement with the observation of reductive coupling in the case of (^*t*Bu^PBP)Ni(CH_3_) but not 1-Me, as the smaller nickel center presumably results in a more congested metal center.^13*g*^ Our results suggest that C–B bond formation is potentially a general decomposition pathway for ^R^PBP supported complexes, rather than a curiosity that is only relevant to a single complex.

Although the reaction between 1-Cl and EtLi results in clean formation of 1-Et, the corresponding reactions between 1-Cl and ^*n*^BuLi, ^i^PrLi, and ^*t*^BuMgCl did not result in the generation of isolable palladium alkyl complexes and instead various decomposition products are observed (Table 1).^24,25^ In the case of ^*t*^BuMgCl, the initial metathesis reaction is slow and even after three days at room temperature some 1-Cl is still present, along with three new peaks in the ^31^P NMR spectra. Although we do not observe (^*t*Bu^PBP)Pd(^*t*^Bu) (1-*^t^*Bu) directly, the peaks observed are consistent with the formation and decomposition of (^*t*Bu^PBP)PdH (1-H) (*vide infra* and see ESI†). 1-H presumably forms *via* β-hydride elimination from 1-*^t^*Bu and in agreement with this proposal iso-butene is observed by ^1^H NMR spectroscopy. This suggests that 1-*^t^*Bu is unstable at room temperature and performing the reaction at low temperature is not possible due to the slow rate of the initial metathesis reaction.

**Table 1:** Summary of the reactions of RMgCl or RLi with (^*t*Bu^PBP)PdCl (1-Cl)

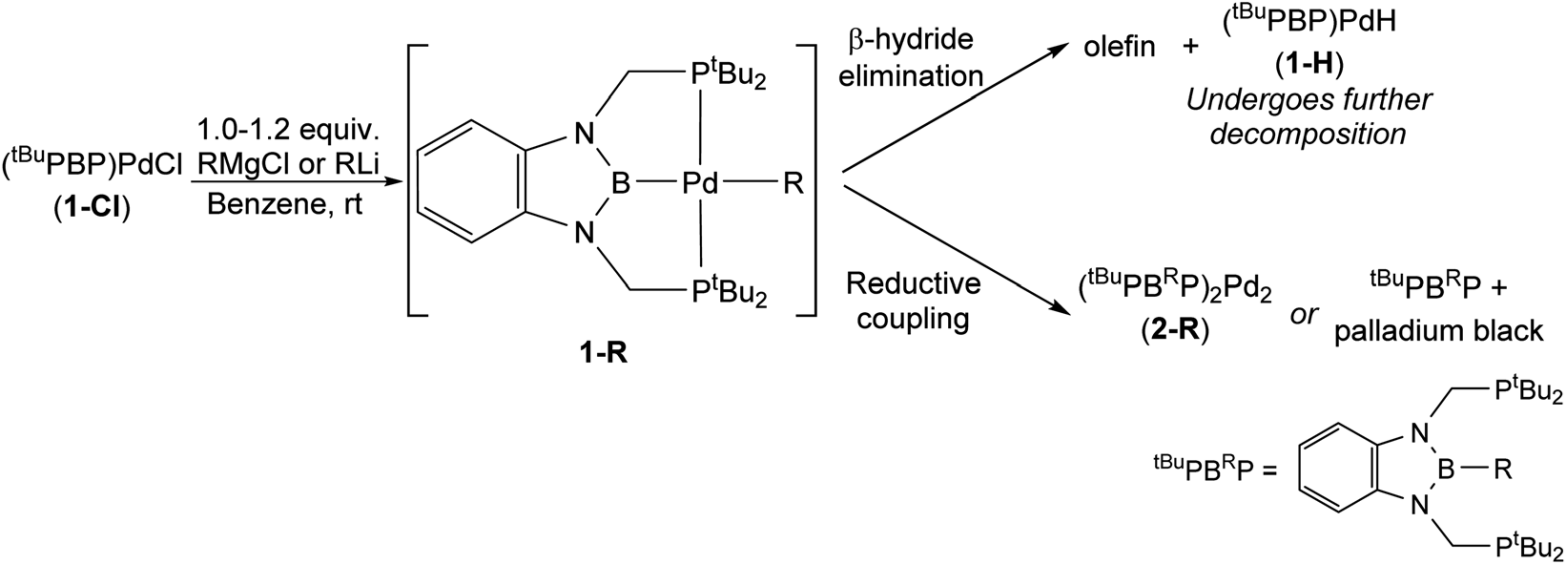				
Entry	Reagent	1-R isolable	Stability of 1-R at rt	Decomposition pathway
1	MeLi	Yes^a^	Stable^a^	Slow reductive coupling (at 65 °C) to give 2-Me (see ESI)
2	EtLi	Yes	Stable	β-Hydride elimination (at 65 °C)
3	^n^PrMgCl	Yes	Stable	β-Hydride elimination (at 60 °C)
4	^*n*^BuLi	No	Unstable	Reductive coupling to give ^*t*Bu^PB^*n*Bu^P and palladium black
5	^i^PrLi	No	Not observed	Reductive coupling to give ^*t*Bu^PB^iPr^P and palladium black
6	^*t*^BuMgCl	No	Not observed	β-Hydride elimination
7	BenzylMgCl	Yes	Stable	Reductive coupling (at 65 °C) to give 2-Bn
8	4-OMe-BenzylMgCl	Yes	Stable	Reductive coupling (at 65 °C) to give 2-^OMe^Bn^b^

a See ref. 13g.

b 2-^OMe^Bn was characterized by analogy to 2-Bn and was not isolated.

The reaction between 1-Cl and ^i^PrLi is rapid and after 10 minutes at room temperature there is no 1-Cl left in the reaction mixture. At this time, one major peak is observed in the ^31^P NMR spectrum at 15.6 ppm, which based on its downfield chemical shift is unlikely to be (^*t*Bu^PBP)Pd(^i^Pr) (1-^i^Pr). Typically, palladium(ii) complexes containing a ^*t*Bu^PBP ligand have chemical shifts between 60–120 ppm, whereas the chemical shift of the free ^*t*Bu^PB^H^P ligand is 17.0 ppm.^26^ There is also no evidence for the generation of products associated with β-hydride elimination from 1-^i^Pr, as no signals corresponding to (^*t*Bu^PBP)PdH (1-H) (or related decomposition products, *vide infra*) are observed in the ^1^H or ^31^P NMR spectra, and there are no resonances associated with propene in the ^1^H NMR spectrum. Instead, we propose that the major species in the ^31^P NMR spectrum is the organic compound ^*t*Bu^PB^iPr^P, which was confirmed by mass spectrometry (see ESI†). We propose that ^*t*Bu^PB^iPr^P forms from the rapid reductive decomposition of 1-^i^Pr, which is generated but not observed in the reaction. The reductive process that 1-^i^Pr is postulated to undergo is akin to what we observed in the decomposition of 1-Bn (*vide supra*) in that a new C–B bond is formed. However, in the case of 1-^i^Pr, we only see the free organic product ^*t*Bu^PB^iPr^P and there is no evidence that ^*t*Bu^PB^iPr^P coordinates to palladium to form a dimer analogous to 2-Bn. Instead, palladium black precipitates out of solution. At this stage, it is unclear why ^*t*Bu^PB^iPr^P does not coordinate to palladium in a similar fashion to ^*t*Bu^PB^Bn^P, and it is possible although unlikely that ^*t*Bu^PB^iPr^P is formed through a pathway that does not even involve the formation of 1-^i^Pr.

The reaction between 1-Cl and ^*n*^BuLi proceeds in an analogous fashion to the reaction between 1-Cl and ^i^PrLi and ultimately gives palladium black and ^*t*Bu^PB^*n*Bu^P. However, the proposed intermediate alkyl complex, (^*t*Bu^PBP)Pd(^*n*^Bu) (1-*^n^*Bu), is more stable and at −35 °C the reaction mixture contained primarily 1-*^n^*Bu (∼97%), with only a small amount of the organic decomposition product ^*t*Bu^PB^*n*Bu^P (∼3%) (see ESI†). Heating the sample to room temperature resulted in an increase in the amount of decomposition product and it was not possible to isolate 1-*^n^*Bu. In contrast, as described above, it is possible to cleanly isolate 1-*^n^*Pr from the reaction between 1-Cl and ^*n*^PrMgCl and decomposition of 1-*^n^*Pr only occurs at 60 °C (eqn (2)). In this case, propene is observed in the ^1^H NMR spectra along with products consistent with the formation and decomposition of 1-H (*vide infra*), suggesting that decomposition primarily occurs *via* β-hydride elimination. Similar decomposition *via* β-hydride elimination is observed at 65 °C for 1-Et, with ethylene observed as a by-product. We also examined the stability of previously reported 1-Me^13*g*^ and demonstrated that at 65 °C it undergoes very slow C–B reductive coupling to form (^*t*Bu^PB^Me^P)_2_Pd_2_ (2-Me) (see ESI†). Our results indicate that the stability of ^*t*Bu^PBP ligated Pd alkyl complexes is related to the steric bulk of the alkyl group, so the order of stability is 1-*^t^*Bu ∼ 1-^i^Pr < 1-*^n^*Bu < 1-*^n^*Pr ∼ 1-Et < 1-Me. Interestingly, the least and most sterically bulky complexes decompose *via* β-hydride elimination, while those with intermediate steric properties decompose through reductive coupling. 1-Me is an exception as it lacks any β-hydrogens and therefore despite its small size decomposes *via* reductive coupling.

**Fig. 5:**
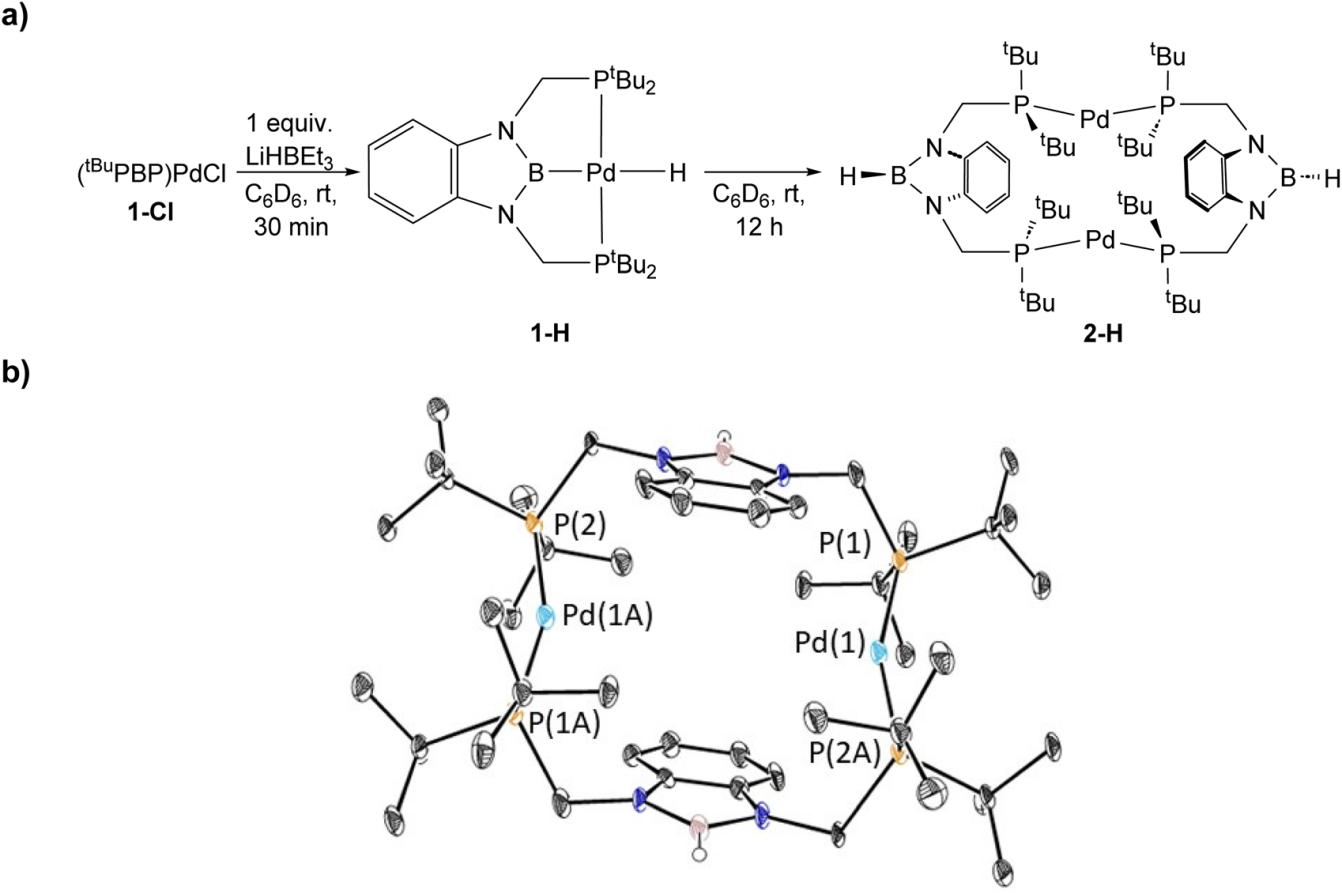
(a) Synthesis and decomposition of ^*t*Bu^PBP supported palladium hydride, 1-H, to form (^*t*Bu^PB^H^P)_2_Pd_2_ (2-H). The decomposition at room temperature in solution prevented the isolated of 1-H. (b) Solid-state structure of 2-H with thermal ellipsoids at 30% probability. Hydrogen atoms are omitted for clarity. Selected distances (Å) and angles (°): Pd(1)–P(1) 2.2821(15), Pd(1)–P(2A) 2.2850(15), P(1)–Pd(1)–P(2A) 159.99(5).

To verify our hypothesis that the rapid decomposition of 1-*^t^*Bu results in the formation of the palladium hydride complex 1-H, we attempted to independently prepare 1-H. Reaction of 1-Cl with 1 equivalent of LiHBEt_3_ in C_6_D_6_ generated two major products in an approximately 55 : 45 ratio by ^1^H and ^31^P NMR spectroscopy (Fig. 5). We propose that one of these is 1-H because the ^31^P NMR chemical shift (115 ppm, 55%) is very close to the shift for the corresponding (^*t*Bu^PBP)PtH complex (114.5 ppm).^27^ The other resonance in the ^31^P NMR spectra is observed at 96.4 ppm, but even though it is present in both the decomposition of 1-*^t^*Bu, 1-Et, and 1-*^n^*Pr, as well as the attempted direct synthesis of 1-H, we are unsure of the identity of the complex giving rise to this signal. Further, although the complex giving rise to the signal at 96.4 ppm is relatively stable over 12 hours, the complex giving rise to the signal at 115 ppm decomposes at room temperature in C_6_D_6_ to give a dimeric palladium(0) complex, 2-H, in which the hydride has reductively coupled with the boron atom of the ^*t*Bu^PBP ligand to give a new H–B bond (Fig. 5). This is an analogous process to the decomposition of 1-Bn, except an H–B bond is formed instead of a C–B bond. 2-H was characterized by single crystal X-ray diffraction (Fig. 5b) and contains two distorted linear palladium(0) centers. The phosphorous atoms of the pincer ligands have rearranged so they bind to two different palladium centers rather than a single palladium center. In this case, it is presumably not steric factors that drive the decomposition of the palladium hydride but the formation of a strong H–B bond. Given the large number of stable pincer-supported palladium hydrides,^28^ we hypothesize that 1-H is unstable because of the *trans*-influence of the boryl ligand, which significantly destabilizes the hydride relative to other species which have weaker *trans*-influence ligands opposite the hydride.

To compare the reactivity of ^*t*Bu^PBP supported palladium alkyl complexes with a ^*t*Bu^PBP supported palladium aryl species, we prepared (^*t*Bu^PBP)Pd(C_6_H_5_) (1-Ph) (eqn (2)). Although, the synthesis of 1-Ph followed the same route as the palladium alkyl species described above,^29^ the reaction of 1-Cl with PhMgBr was significantly slower than the corresponding reactions with alkyl lithium or Grignard reagents. Specifically, the reaction with PhMgBr took two days to reach completion at room temperature, whereas the reactions with alkyl lithium or Grignard reagents were typically complete in less than one hour at room temperature (except for ^*t*^BuMgCl). This is likely related to the lower nucleophilicity of aryl Grignard reagents compared with alkyl Grignard reagents. After recrystallization to remove Mg salt impurities, we were able to isolate 1-Ph in 66% yield. 1-Ph was characterized by X-ray crystallography (see ESI†). The Pd–C bond distance in is 2.162(3) Å, which is significantly shorter than the Pd–C bond length in all of our palladium alkyl complexes. This is likely due to the fact that the carbon atom bound to palladium in 1-Ph is sp^2^-hybridized and is consistent with the trend observed for PCP-supported pincer complexes.^14^

### Reactivity of ^*t*Bu^PBP supported palladium alkyl and aryl complexes with CO_2_

**Fig. 6:**
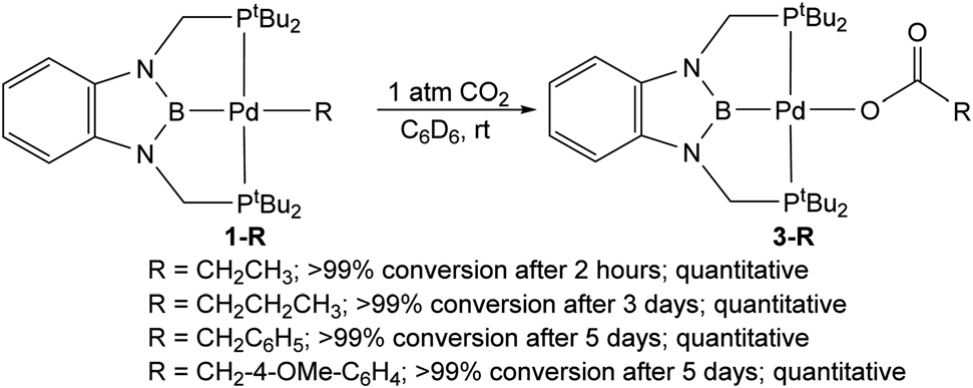
Relative rates of CO_2_ insertion into ^*t*Bu^PBP supported palladium alkyl complexes to form palladium carboxylate complexes.

**Fig. 7:**
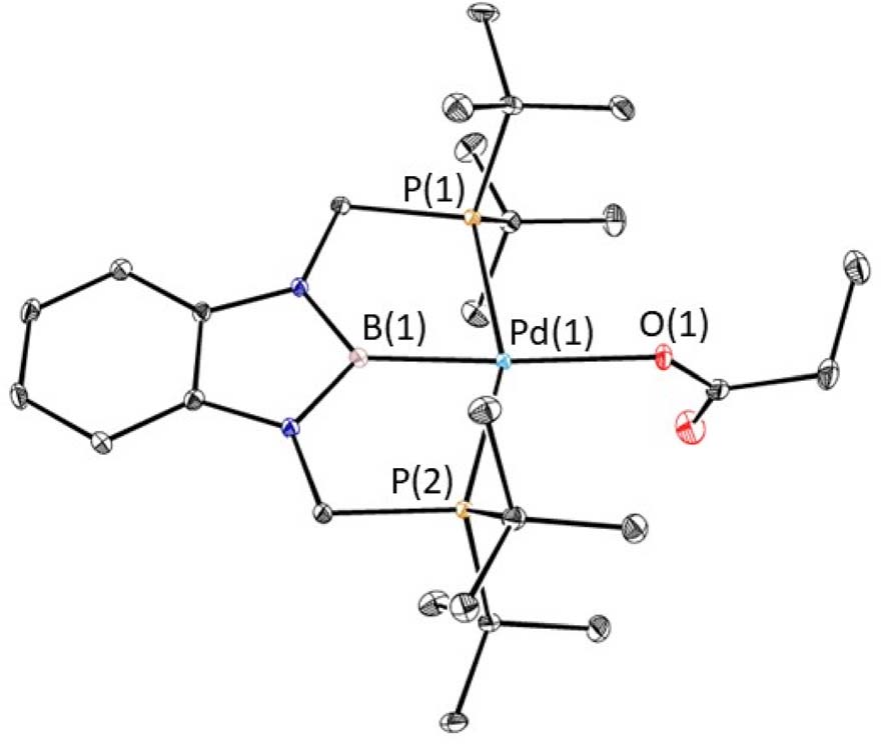
Solid-state structure of 3-Et with thermal ellipsoids at 30% probability. Hydrogen atoms are omitted for clarity. Selected distances (Å) and angles (°): Pd(1)–B(1) 1.973(2), Pd(1)–O(1) 2.1803(14), Pd(1)–P(1) 2.3449(5), Pd(1)–P(2) 2.3450(5), B(1)–Pd(1)–O(1) 173.80(7), B(1)–Pd(1)–P(1) 78.20(7), B(1)–Pd(1)–P(2) 78.45(7), O(1)–Pd(1)–P(1) 101.78(4), O(1)–Pd(1)–P(2) 101.30(4), P(1)–Pd(1)–P(2) 156.617(19), Pd(1)–O(1)–C(1) 123.46(14).

The reaction of 1-Et with 1 atm of CO_2_ in C_6_D_6_ at room temperature quantitively generated the palladium carboxylate complex (^*t*Bu^PBP)Pd{OC(O)CH_2_CH_3_} (3-Et) in approximately 2 hours (Fig. 6). This is the fastest rate of CO_2_ insertion observed for a pincer supported palladium alkyl species. 3-Et was isolated and characterized using single crystal X-ray diffraction (Fig. 7). The solid-state structure confirms κ^1^-binding of the carboxylate. The Pd–O bond distance is 2.1803(14) Å, which is relatively long for a palladium(ii) carboxylate complex.^13*g*,14*a*,30^ This suggests that it will be easier to cleave the Pd–O bond in 3-Et compared to related palladium carboxylate complexes, which have been generated *via* CO_2_ insertion reactions.^6,14*a*^ The Pd–B bond distance is significantly shorter in 3-Et (1.973(2) Å) compared with 1-Et (2.029(7) Å), which is consistent with the carboxylate ligand exerting a significantly weaker *trans*-influence than the ethyl ligand.

**Fig. 8:**
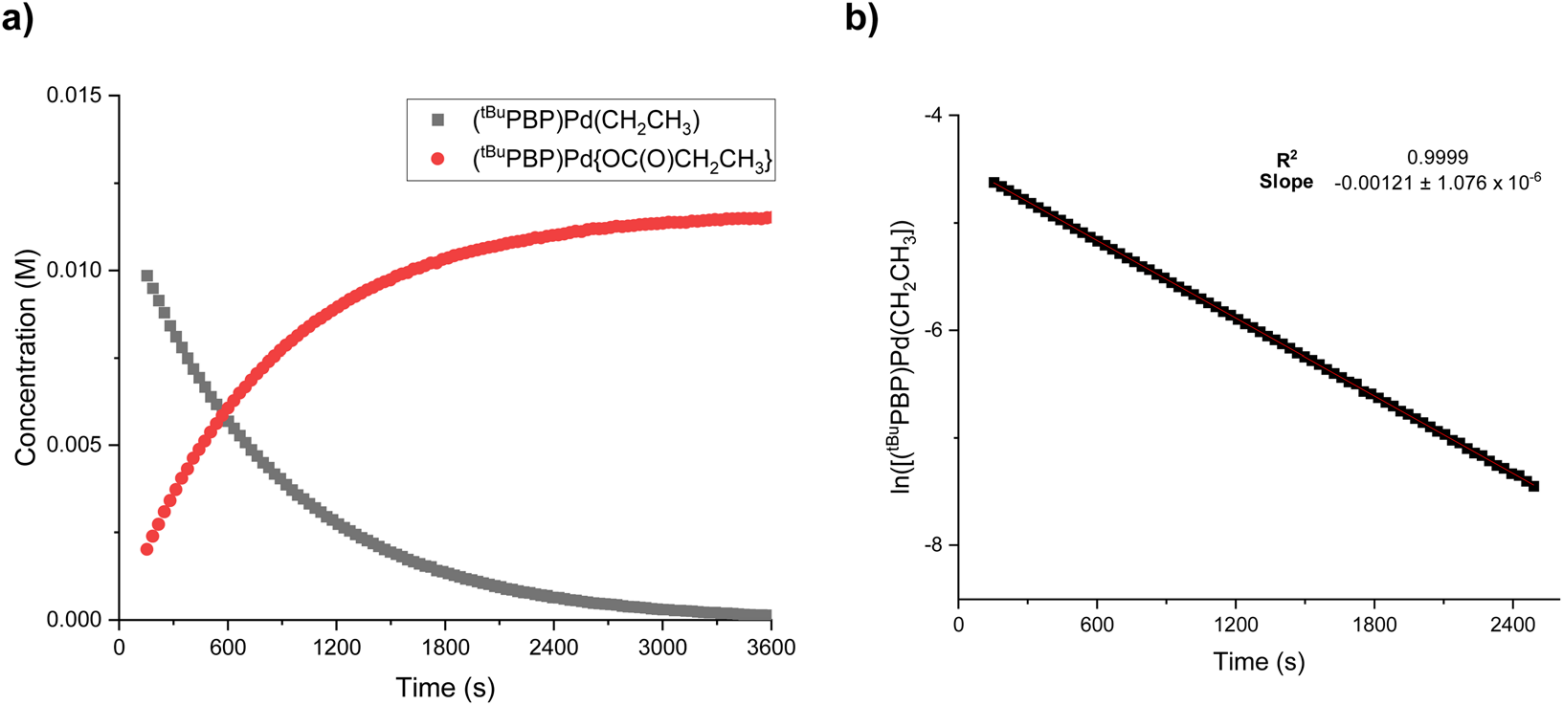
Representative traces for the insertion of CO_2_ into (^*t*Bu^PBP)Pd(CH_2_CH_3_) (1-Et) at 30 °C in C_6_D_6_ with 1 atm of CO_2_ showing (a) the concentrations of (^*t*Bu^PBP)Pd(CH_2_CH_3_) (1-Et) and (^*t*Bu^PBP)Pd{OC(O)CH_2_CH_3_} (3-Et) as a function of time and (b) the ln of the concentration of (^*t*Bu^PBP)Pd(CH_2_CH_3_) (1-Et) as a function of time.

The rapid insertion of CO_2_ into 1-Et at room temperature enabled us to use NMR spectroscopy to measure the kinetics of the reaction. We performed kinetics experiments under pseudo-first order conditions with an excess of CO_2_ and measured both the disappearance of 1-Et and the appearance of 3-Et (Fig. 8a). The reaction is first order in both 1-Et and [CO_2_], so the overall rate law is *k*_1_[(^*t*Bu^PBP)Pd(CH_2_CH_3_)][CO_2_] (Fig. 8b and ESI†). We were able to obtain values of *k*_1_ at different temperatures by dividing the *k*_obs_ values obtained from a plot of ln([(^*t*Bu^PBP)Pd(CH_2_CH_3_)]) *versus* time by the concentration of CO_2_ (Table 2 and ESI†). The most striking feature of our *k*_1_ values is that at 40 °C, the rate constant for CO_2_ insertion into 1-Et is *more than double* the rate constant for insertion into 1-Me that we measured previously.^13*g*^ In the only other comparative study of the rates of CO_2_ insertion into metal methyl and ethyl species, Darensbourg and co-workers observed that CO_2_ insertion into [RW(CO)_5_]^−^ (R = CH_3_ or CH_2_CH_3_) is 1.5 times faster for methyl than for ethyl, the opposite trend to our system.^8*b*^ At this stage, given the paucity of other comparative studies on the rates of CO_2_ insertion into different metal alkyls, it is unclear if either result is an outlier or the nature of the underlying factors that cause the variation in trends.

**Table 2:** Comparison of the rate constants for CO_2_ insertion into (^*t*Bu^PBP)Pd(alkyl) at various temperatures, solvents, and 1 atm of CO_2_

Entry	Complex	Temperature (°C)	Solvent	*k*_1_ (M^−1^ s^−1^ × 10^−2^)^a^
1	1-Et	25	C_6_D_6_	1.2
2	1-Et	30	C_6_D_6_	2.1
3	1-Et	35	C_6_D_6_	2.9
4	1-Et	40	C_6_D_6_	3.4
5	1-Et	45	C_6_D_6_	4.7
6	1-Me	40	C_6_D_6_	1.3
7	1-Me	45	C_6_D_6_	2.3
8	1-Bn	30	Pyridine-*d*_5_	0.48
9	1-^OMe^Bn	30	Pyridine-*d*_5_	0.43

a These values are the average of two trials and the errors are ±10%.

Using the values of *k*_1_ at different temperatures we determined the activation parameters for CO_2_ insertion into 1-Et through Eyring analysis. The enthalpy of activation, Δ*H*^‡^, is 11.3 ± 1.1 kcal mol^−1^, the entropy of activation, Δ*S*^‡^, is −29.0 ± 2.9 cal mol^−1^ K^−1^, and Δ*G*^‡^_298_ is 20.0 ± 2.0 kcal mol^−1^ (see ESI†). All of these values are within error to those previously measured for 1-Me,^13*g*^ suggesting that the reactions proceed *via* similar pathways. The enthalpy for CO_2_ insertion into 1-Et is lower than that observed for insertion into palladium methyl complexes with pincer ligands that contain a lower *trans*-influence donor in the central position. For example, Δ*H*^‡^ for CO_2_ insertion into (^*t*Bu^PCP)Pd(CH_3_) (^*t*Bu^PCP = 2,6-C_6_H_3_(CH_2_P^*t*^Bu_2_)_2_) is 17.4 ± 1.7 kcal mol^−1^.^14*b*^ This is consistent with the ^*t*Bu^PBP ligand destabilizing 1-Et by weakening the Pd–C bond of the palladium ethyl ligand. The negative entropy of activation is similar to those observed in other systems for CO_2_ insertion^13*g*,14*b*^ and is in agreement with a rate-limiting transition state in which two molecules are combining to form one compound in the transition state.

The reaction of 1-*^n^*Pr with 1 atm of CO_2_ in C_6_D_6_ at room temperature also cleanly generated the palladium carboxylate complex (^*t*Bu^PBP)Pd{OC(O)CH_2_CH_2_CH_3_} (3-*^n^*Pr) (Fig. 6). Surprisingly, CO_2_ insertion into 1-*^n^*Pr is significantly slower than the corresponding insertion reactions with 1-Me and 1-Et. In the case of 1-*^n^*Pr, the reaction required 3 days to reach completion at room temperature.^31^ This slow rate of insertion precluded the measurement of a rate constant using our NMR method, but based on the reaction half-life of approximately 11 hours we estimate that insertion into 1-*^n^*Pr occurs approximately *one order of magnitude slower than* the rate of insertion into 1-Me. Further, we were unable to heat the reaction of 1-*^n^*Pr with CO_2_ to sufficiently speed up the reaction, as this resulted in decomposition of 1-*^n^*Pr. Overall, our results show that the simple change in alkyl group from 1-Me to 1-Et to 1-^i^Pr results in significant and non-intuitive changes in the rates of CO_2_ insertion. This is potentially important in catalysis as it implies the rate of CO_2_ insertion into palladium alkyl complexes (and potentially other metal alkyl complexes) will be heavily substrate dependent.

The reactions of the benzyl complexes 1-Bn and 1-^OMe^Bn with 1 atm of CO_2_ at room temperature formed the carboxylate complexes (^*t*Bu^PBP)Pd{OC(O)CH_2_C_6_H_5_} (3-Bn) and (^*t*Bu^PBP)Pd{OC(O)CH_2_-4-OMe-C_6_H_4_} (3-^OMe^Bn), respectively (Fig. 6). These reactions were significantly slower than the corresponding insertion reactions with 1-Me, 1-Et, or even 1-*^n^*Pr. For example, in C_6_D_6_ at room temperature the reactions took approximately 5 days to reach completion. The slower rate of insertion into palladium benzyl complexes compared with palladium n-alkyl complexes is unsurprising as the benzylic carbon bound to palladium is expected to be a worse nucleophile due to the electron-withdrawing nature of the aromatic group, which makes it less energetically favorable to attack electrophilic CO_2_. In order to measure the kinetics of insertion into 1-Bn and 1-^OMe^Bn we needed to increase the rate of the reaction. Unfortunately, it is not possible to raise the temperature to promote CO_2_ insertion into 1-Bn in C_6_D_6_ as this leads to decomposition to form the palladium(0) complex, 2-Bn, as well as the CO_2_ inserted product. However, we have previously demonstrated that the rates of CO_2_ insertion reactions can be increased by performing the reaction in solvents with a higher Dimroth–Reichardt *E*_T_(30) parameter,^13*g*,32^ which is an empirical measure of the polarity of a solvent.^33,34^ When CO_2_ insertion reactions were performed in pyridine-*d*_5_, the reactions with 1-Bn and 1-^OMe^Bn were complete in approximately 5 hours at room temperature, with no evidence for the formation of palladium(0) complexes. This again highlights the dramatic effect of solvent on CO_2_ insertion reactions.^13*g*,32,35^ At 30 °C, the rate constants for CO_2_ insertion into 1-Bn and 1-^OMe^Bn in pyridine-*d*_5_ were 0.0048 ± 0.0005 and 0.0043 ± 0.0004 M^−1^ s^−1^, respectively. The fact that these values are the same within error indicates that the substitution on the phenyl ring surprisingly does not significantly affect the nucleophilicity of the carbon bound to palladium and means that in the catalytic carboxylation of benzylic substrates^36^ the electronic effect of the substituents on the rate of CO_2_ insertion is likely minimal. Unfortunately, we are unable to measure the rate constants for CO_2_ insertion into 1-Me or 1-Et in pyridine-*d*_5_ because the reaction occurs too fast to obtain an accurate rate constant using NMR spectroscopy. However, we estimate a minimum rate constant of 0.2 M^−1^ s^−1^, which is significantly faster than for the benzyl compounds.

In contrast to our results with palladium alkyl complexes, no reaction was observed when 1-Ph was treated with 1 atm of CO_2_, even after prolonged heating at elevated temperature. Although at this stage it is unclear whether kinetic or thermodynamic factors are responsible for the lack of reactivity, our result is consistent with observations for other pincer supported Group 10 phenyl complexes, which also do not react with CO_2_.^13*b*,*e*^ Hence, although the PBP ligand promotes CO_2_ insertion into palladium alkyl complexes, it does not facilitate insertion reactions into palladium aryl complexes.

### Computational studies of CO_2_ insertion into palladium alkyl and aryl complexes

**Fig. 9:**
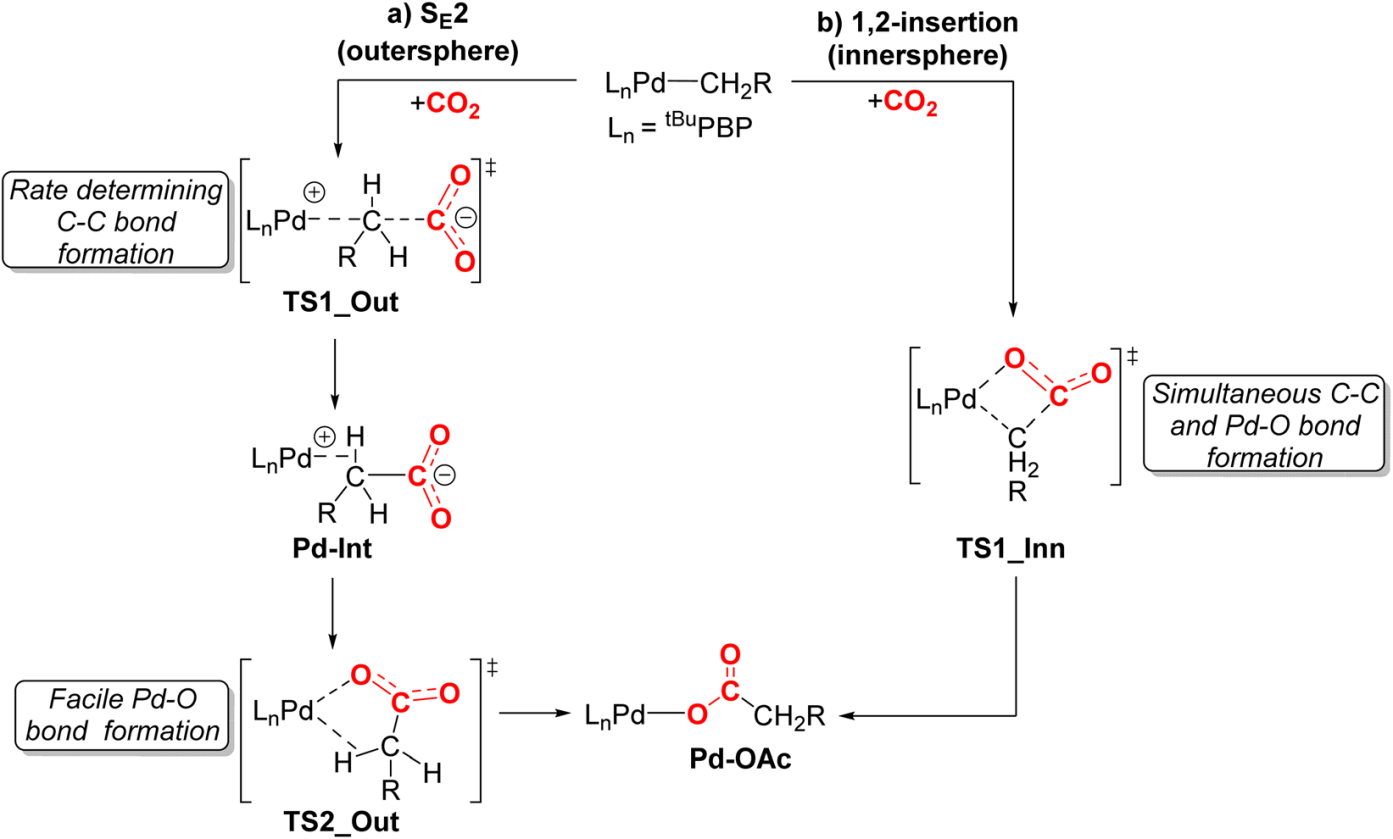
Two plausible mechanisms for CO_2_ insertion into pincer-supported palladium alkyl complexes: (a) S_E_2 (outersphere) and (b) 1,2-insertion (innersphere). For 1-Me we previously demonstrated that the S_E_2 pathway is lower energy and the initial nucleophilic attack of the carbon atom of the methyl group on CO_2_ is rate-determining.^13*g*^

We performed DFT calculations (PBE0-D3BJ, IEFPCM) to further understand the mechanism of CO_2_ insertion into 1-Me, 1-Et, 1-*^n^*Pr, 1-Bn, and 1-^OMe^Bn. Previously, we have demonstrated that CO_2_ insertion into 1-Me follows an S_E_2 (or outersphere) pathway (Fig. 9a),^13*g*^ in which the first and rate-limiting step is nucleophilic attack of the carbon atom of the palladium methyl on the electrophilic carbon atom of CO_2_ to form the C–C bond. Notably, there is no interaction between CO_2_ and the palladium center at the transition state. The second step in CO_2_ insertion into 1-Me*via* an S_E_2 pathway has a significantly lower barrier and involves the rearrangement of an carboxylate-palladium ion pair, bound through a C–H σ-bond, to the neutral Pd–O containing product.^13*g*^ An alternative pathway involving 1,2-insertion (innersphere, Fig. 9b) in which both the Pd–O and C–C bonds are formed at the same transition state was calculated to be energetically unfavorable for 1-Me.

**Fig. 10:**
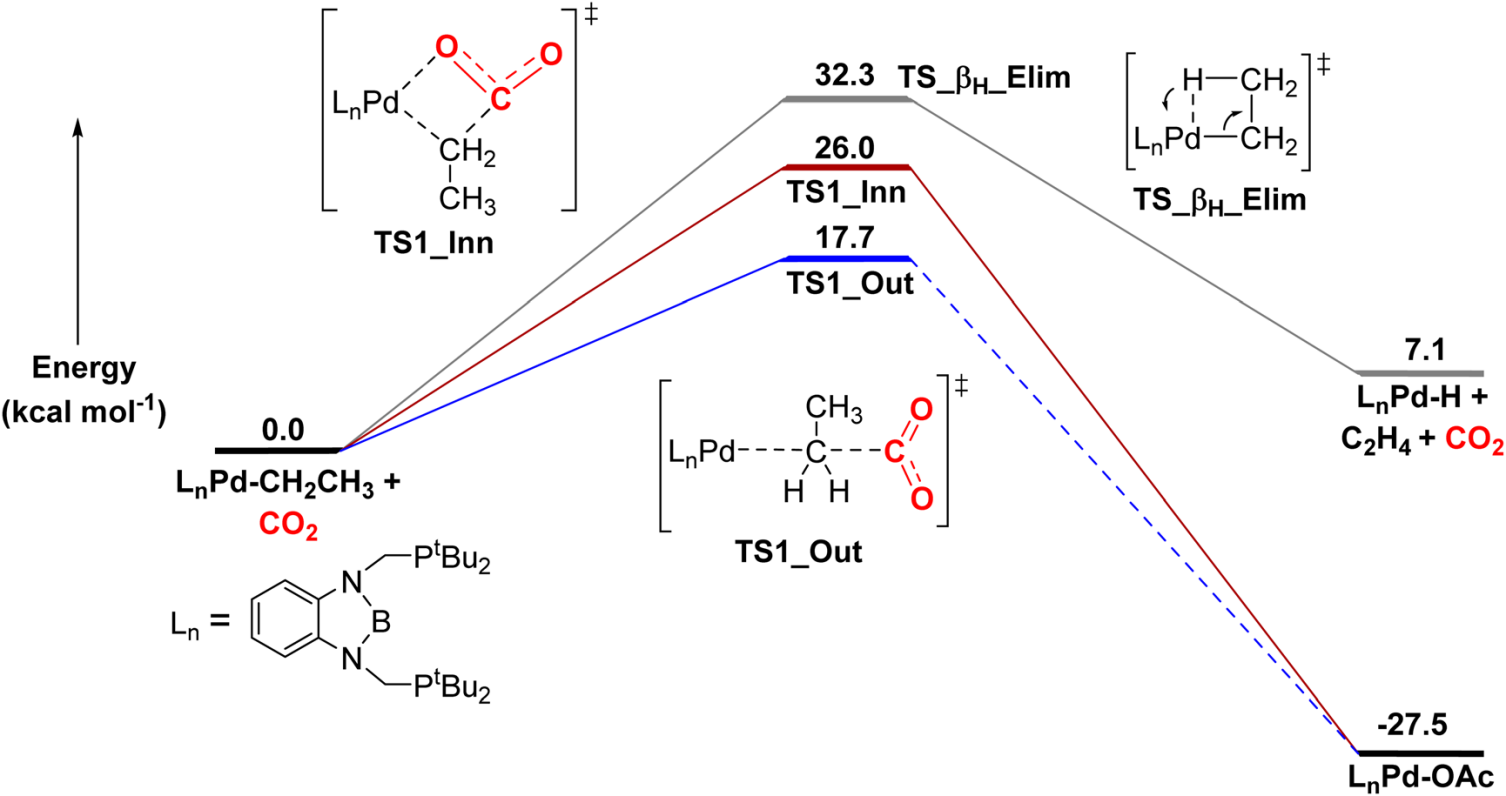
Relative free energies of CO_2_ insertion into 1-Et*via* an S_E_2 (outersphere) and 1,2-addition (innersphere) pathway, as well as the energy for β-hydride elimination. In the S_E_2 pathway, we were unable to find the intermediate and barrier for the second rearrangement step to form the palladium carboxylate product, as the rearrangement occurs spontaneously during geometry optimization. However, this process has previously been demonstrated to be low energy in related systems.^13*g*^

Here, we calculated that for CO_2_ insertion into 1-Et, the barrier for the first step in the S_E_2 pathway is 17.7 kcal mol^−1^ at 298 K (Fig. 10, Table 3).^37^ This is in good agreement with the experimentally determined barrier of 20.0 ± 2.0 kcal mol^−1^ (*vide supra*). The barrier for the innersphere 1,2-insertion pathway is calculated to be 26.0 kcal mol^−1^, unambiguously indicating that the S_E_2 pathway is preferred. A surprising feature of 1-Et is that it does not undergo facile β-hydride elimination. We calculated that the barrier for β-hydride elimination is relatively high (32.3 kcal mol^−1^), consistent with the stability of the complex towards β-hydride elimination. β-Hydride elimination is presumably disfavored because of the rigidity of the pincer ligand, which makes it energetically difficult for the complex to distort to form the syn co-planar arrangement of the palladium, C_α_, C_β_, and H required for β-hydride elimination.

**Table 3:** Calculated barriers for CO_2_ insertion into (^R^PBP)Pd(alkyl) complexes

Complex	Δ*G*^‡^ (kcal mol^−1^)
(^*t*Bu^PBP)Pd(CH_3_) (1-Me)	19.4
(^*t*Bu^PBP)Pd(CH_2_CH_3_) (1-Et)	17.7
(^*t*Bu^PBP)Pd(CH_2_CH_2_CH_3_) (1-*^n^*Pr)	21.8
(^*t*Bu^PBP)Pd{CH(CH_3_)_2_} (1-^i^Pr)	28.2
(^*t*Bu^PBP)Pd(CH_2_C_6_H_5_) (1-Bn)	20.3
(^*t*Bu^PBP)Pd(CH_2_-4-OMeC_6_H_4_) (1-^OMe^Bn)	20.5
(^*t*Bu^PBP)Pd(CH_2_-4-CF_3_C_6_H_4_) (1-^CF3^Bn)	20.6
(^*t*Bu^PBP)Pd(C_6_H_5_) (1-Ph)	34.6^a^
(^iPr^PBP)Pd(CH_3_)	16.8^a^
(^iPr^PBP)Pd(CH_2_CH_3_)	18.7
(^iPr^PBP)Pd(CH_2_CH_2_CH_3_)	20.3
(^Me^PBP)Pd(CH_3_)	17.5^a^
(^Me^PBP)Pd(CH_2_CH_3_)	17.3^a^
(^Me^PBP)Pd(CH_2_CH_2_CH_3_)	17.5^a^

a The lowest energy pathway for CO_2_ insertion is the 1,2-insertion (or innersphere) mechanism rather than the S_E_2 (or outersphere) mechanism.

DFT calculations predict that the barriers for CO_2_ insertion into 1-Me and 1-*^n^*Pr are 19.4 kcal mol^−1^ and 21.8 kcal mol^−1^(Table 3), respectively, which means that the calculations are in line with the experimental trends in rate (1-Et > 1-Me > 1-*^n^*Pr).^38^ The rate-determining transition states for CO_2_ insertion into 1-Me, 1-Et, and 1-*^n^*Pr are analogous (the first step in the S_E_2 mechanism), so the differences in rate are not related to a change in mechanism. Instead, we propose that the difference in rates is due primarily to steric factors, which affect the relative stability of both the reactant complexes and the transition states. 1-Et and 1-*^n^*Pr are likely slightly destabilized relative to 1-Me because of steric interactions between the ethyl or propyl ligand and the *tert*-butyl substituents of the ^*t*Bu^PBP ligand. This is reflected by the increased thermodynamic favorability of CO_2_ insertion into 1-Et and 1-*^n^*Pr compared to 1-Me (Δ*G*° = −27.5 and −26.2 kcal mol^−1^, respectively, *versus* −22.9 kcal mol^−1^). Presumably, in the carboxylate complexes, the steric pressure is relieved because of the absence of hydrogens on the oxygen bound to palladium.

**Fig. 11:**
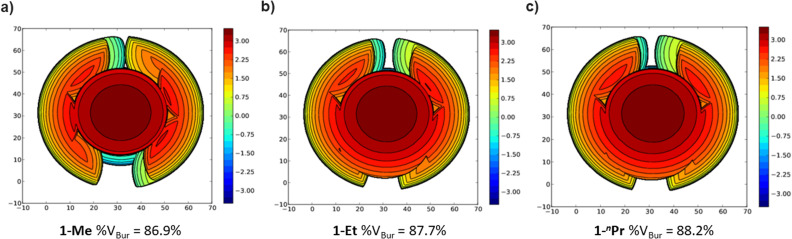
Topographic steric maps of (a) 1-Me, (b) 1-Et, and (c) 1-*^n^*Pr as viewed down the C–Pd bond towards the plane defined by P–Pd–P.^39^

**Fig. 12:**
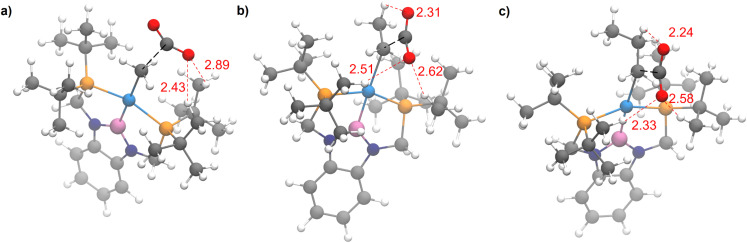
Rate determining transition states for CO_2_ insertion into (a) 1-Me, (b) 1-Et, and (c) 1-*^n^*Pr. Close contacts between ligand C–H bonds and the incipient carboxylate group are highlighted in red. The bond forming atoms are connected by a dotted black line.

The steric properties of 1-Me, 1-Et, and 1-*^n^*Pr were quantitatively evaluated by calculating the percent buried volume (%*V*_Bur_) of these complexes based on their crystal structures using the Salerno molecular buried volume program (SambVca 2.1) (Fig. 11).^39^ Although there is only a small difference in %*V*_Bur_ between the three complexes (86.9% for 1-Me, 87.7% for 1-Et, and 88.2% for 1-*^n^*Pr) the absolute magnitude of these numbers indicates the high degree of steric crowding around the palladium centers and suggests that small changes could have a significant impact on the rate of CO_2_ insertion. Our proposal is that 1-Et is sufficiently sterically crowded to destabilize the ethyl ligand, but still open enough for CO_2_ to easily approach the ethyl group. This increases the rate of insertion in comparison to 1-Me. In contrast, even though 1-*^n^*Pr is sufficiently sterically crowded to destabilize the n-propyl ligand, the complex is so congested that it is unfavorable for CO_2_ to approach, which increases the barrier for insertion. Consistent with this proposal, the computed barrier for CO_2_ insertion into the even more sterically congested (^*t*Bu^PBP)Pd{CH(CH_3_)_2_} (1-^i^Pr) increases to 28.2 kcal mol^−1^ (Table 3). Finally, the trajectory of electrophilic attack of CO_2_ is quite different for 1-Me compared to 1-Et and 1-*^n^*Pr. In 1-Me the CO_2_ is nearly orthogonal to the palladium methyl bond, whereas in 1-Et and 1-*^n^*Pr is it essentially co-planar (Fig. 12). In 1-Et and 1-Pr this geometry leads to close contacts between three C–H bonds and the incipient carboxylate group, which may help stabilize the emerging charge on the carboxylate group. We were unable to locate a similar TS geometry for insertion into 1-Me, and in the calculated TS there are only interactions between two C–H bonds and the incipient carboxylate group. In the case of 1-Pr, destabilization due to steric strain is likely a larger force than stabilization due to an extra non-covalent interaction and therefore the overall barrier is higher.

To further probe the role of steric factors on the rates of CO_2_ insertion, we performed calculations on the smaller model complexes (^R^PBP)Pd(alkyl) (R = Me or ^i^Pr; alkyl = CH_3_, CH_2_CH_3_, or CH_2_CH_2_CH_3_). Interestingly, in the case of ^Me^PBP, DFT predicts that the preferred CO_2_ insertion pathway changes from S_E_2 to 1,2-insertion for all tested alkyls (see ESI†), suggesting that the size of the ligand is crucial in determining the reaction pathway. We propose that complexes with a smaller steric profile are more likely to react *via* a 1,2-insertion pathway compared with complexes that are more congested, because in this case it is easier for CO_2_ to interact with the metal center. Further, in the case of ^Me^PBP supported complexes, the calculated rates of insertion are the same for the methyl, ethyl, and n-propyl species, suggesting that the nature of the alkyl group is less important for systems that react through a 1,2-insertion pathway. This is unsurprising, as in the 1,2-insertion pathway the metal center is directly involved, which likely lessens the impact of the alkyl group. For the ^iPr^PBP supported palladium complexes, 1,2-insertion is preferred for (^iPr^PBP)Pd(CH_3_), whereas the S_E_2 pathway is preferred for the ethyl and propyl complexes, with the later showing higher barriers than (^iPr^PBP)Pd(CH_3_). The barrier for insertion into (^iPr^PBP)Pd(CH_2_CH_3_) is lower than for (^iPr^PBP)Pd(CH_2_CH_2_CH_3_), indicating that there is a steric effect with the ^iPr^PBP pincer ligand. The computed results with the smaller ancillary ligands suggest that the observation that CO_2_ insertion is faster for 1-Et than for 1-Me or 1-*^n^*Pr is unlikely to be general to all systems. The ^*t*Bu^PBP ligand creates a sufficiently crowded environment where a minor change in the sterics results in non-intuitive changes in rate, whereas for other supporting ligands this will not be the case, as evidenced by our calculated results with ^iPr^PBP and ^Me^PBP.

We next investigated the barriers for CO_2_ insertion into the palladium benzyl complexes 1-Bn, 1-^OMe^Bn, as well as the hypothetical complex (^*t*Bu^PBP)Pd(CH_2_-4-CF_3_-C_6_H_4_) (1-^CF3^Bn) (Table 3). The calculated barrier for CO_2_ insertion into 1-Bn (20.3 kcal mol^−1^) is higher than for 1-Me and 1-Et, in agreement with our experimental observations. In contrast, we computationally predict that insertion into 1-*^n^*Pr is more challenging than insertion into 1-Bn, which contradicts our experimental results, but likely reflects some computational error. When calculations were performed with other functionals (see ESI†), there were cases where the barrier for insertion into 1-Bn was higher than for 1-*^n^*Pr, indicating that different DFT functionals provide slighty different TS structures. The lowest energy pathway for insertion into 1-Bn involves an S_E_2 mechanism and the geometric parameters at the transition state for insertion are similar to those observed for 1-Me, 1-Et, and 1-*^n^*Pr. Previous calculations on CO_2_ insertion into palladium benzyl species have also invoked an S_E_2 pathway.^40^ Calculations on CO_2_ insertion into 1-^OMe^Bn or 1-^CF3^Bn indicate that the barriers for insertion into these species are approximately the same as for the unsubstituted palladium benzyl species. This is unexpected as it suggests that the impact of the para-substituent on the nucleophilicity of the benzylic carbon is negligible even though this substituent should impact the energy of the π*-orbital which stabilizes or destabilizes the carbon. Nevertheless, our calculations are in agreement with the experimental rate constants of 1-Bn and 1-^OMe^Bn being within error (*vide supra*).

In contrast to the facile insertion of CO_2_ into palladium alkyl complexes supported by ^*t*Bu^PBP ligands, we did not observe CO_2_ insertion into 1-Ph. To understand this reactivity, we calculated the kinetic and thermodynamic parameters associated with CO_2_ insertion into 1-Ph. Although the reaction is considerably thermodynamically downhill (by −19.2 kcal mol^−1^), the kinetic barrier is 34.6 kcal mol^−1^, which explains why no reaction is observed experimentally. Consistent with our previous results for CO_2_ insertion into palladium–C(sp^2^) bonds,^40^ the transition state is classified as innersphere, with a Pd–C_CO_2__ interaction of 3.03 Å. To understand the influence of the ^*t*Bu^PBP ligand on CO_2_ insertion into 1-Ph, we calculated the energy of the transition state for CO_2_ insertion into (^*t*Bu^PCP)Pd(C_6_H_5_) (^*t*Bu^PCP = 2,6-C_6_H_3_(CH_2_P^*t*^Bu_2_)_2_), which features a pincer ligand with a lower *trans*-influence donor opposite the phenyl group. In this case, the activation energy associated with an innersphere transition state is 46.3 kcal mol^−1^, indicating that influence of the ^*t*Bu^PBP ligand is significant. However, in order for CO_2_ insertion to become kinetically viable experimentally, a different approach needs to be adopted than introducing a stronger *trans*-influence ligand opposite the phenyl ligand, as the ^*t*Bu^PBP ligand is one of the strongest *trans*-influence ligands available and it does not lower the activation energy for CO_2_ insertion enough for the reaction to proceed under mild conditions.

## Conclusions

In this work, we prepared and crystallographically characterized an unusual series of ^*t*Bu^PBP supported palladium alkyl and aryl complexes including species with ethyl, n-propyl, benzyl, and phenyl ligands. In contrast, ^*t*Bu^PBP supported palladium complexes with *n*-butyl, iso-propyl, and *tert*-butyl ligands were either unstable or could not be observed. The rates of decomposition of the alkyl complexes are related to the steric bulk of the alkyl ligand with a putative *tert*-butyl complex decomposing faster than the ethyl species. The palladium alkyl complexes decompose *via* two different routes. The least and most sterically bulky complexes containing ethyl or *tert*-butyl ligands decompose *via* β-hydride elimination. In contrast, complexes containing *n*-butyl and iso-propyl ligands are stable towards β-hydride elimination and along with benzyl complexes decompose *via* an uncommon reductive coupling reaction, which involves the formation of a new C–B bond and either well-defined palladium(0) dimers or palladium black. Attempts to synthesize a ^*t*Bu^PBP supported palladium hydride were unsuccessful because a similar reductive coupling occurred to generate a dimeric palladium(0) complex with two new H–B bonds. The observation of decomposition *via* reductive coupling across a series of complexes confirms that this is a general reaction for ^R^PBP supported complexes.

^*t*Bu^PBP ligated palladium complexes with ethyl, n-propyl, and benzyl ligands all cleanly insert CO_2_ to form the corresponding carboxylate complexes, allowing for a rare study of the rates of CO_2_ insertion across an analogous series of metal alkyl complexes. Kinetic studies demonstrate that the rate of CO_2_ insertion into 1-Et is more than double the rate for insertion into 1-Me, which in turn is ten times faster than the rate of insertion into 1-*^n^*Pr. CO_2_ insertion into ^*t*Bu^PBP supported palladium benzyl complexes is much slower than insertion into n-alkyl complexes, likely because the carbon atom of the benzyl group is less nucleophilic. DFT calculations indicate that insertion reactions into 1-Me, 1-Et, 1-*^n^*Pr, and 1-Bn proceed *via* an outersphere S_E_2 pathway and steric factors are responsible for the observed differences in rate between the n-alkyl complexes. They also suggest that the counterintuitive trends in the rates of CO_2_ insertion observed in the present work will not occur for all metal alkyl systems but are related to the specific steric factors present in this group of complexes. Although the strong *trans*-influence of the boryl ligand in ^*t*Bu^PBP promotes CO_2_ insertion into palladium alkyl complexes, no reaction is observed between 1-Ph and CO_2_. Overall, our results highlight how the rates of CO_2_ vary across an analogous series of palladium alkyl and aryl complexes and DFT calculations provide explanations for the observed trends. This information will be valuable for the development of catalytic reactions that involve CO_2_ insertion into metal alkyl bonds as an elementary step.

## Data availability

Crystallographic data has been deposited at the CCDC with numbers 2243696–2243703 and 2264960. NMR spectra and the coordinates and energies of DFT optimized geometries are provided as part of the ESI.†

## Author contributions

APD and NH conceptualized the project. APD performed experimental investigations and SG performed computational investigations. NH supervised experimental studies and KHH supervised computational studies. All authors were involved in writing the manuscript.

## Conflicts of interest

The authors declare no competing financial interests.

## Supplementary Material

SC-014-D3SC01459B-s001

SC-014-D3SC01459B-s002

SC-014-D3SC01459B-s003
