# Supplementary material for: Comparative study of CO2 insertion into pincer supported palladium alkyl and aryl complexes
Source: Chem Sci. 2023 Jul 12;14(30):8164–79. doi: 10.1039/d3sc01459b (PMC10395277; doi:10.1039/d3sc01459b)
Supplement: SC-014-D3SC01459B-s002 [file SC-014-D3SC01459B-s002.pdf]

Electronic Supplementary Material (ESI) for Chemical Science.
This journal is © The Royal Society of Chemistry 2023

Supporting Information: Comparative Studies of CO_2 Insertion into Pincer Supported Palladium Alkyl and Aryl Bonds

Anthony P. Deziel,[a] Sahil Gahlawat,[b,c] Nilay Hazari,[a,*] Kathrin H. Hopmann,[b,*] & Brandon Q. Mercado[a]

[a]Department of Chemistry, Yale University, P. O. Box 208107, New Haven, Connecticut, 06520, USA. E-mail: nilay.hazari@yale.edu.

[b]Department of Chemistry, UiT The Arctic University of Norway, N-9307 Tromsø, Norway. E-mail: kathrin.hopmann@uit.no.

[c]Hylleraas Center for Quantum Molecular Sciences, UiT The Arctic University of Norway, 9307 Tromsø, Norway.

SI: Experimental Details S2

SII: Synthesis and Characterization of New Compounds S3

SIII: Attempted Synthesis of Unstable (^{tBu}PBP)Pd-C$_{(alkyl)}$ and Related Complexes S9

SIV: Determination of Qualitative Rates of CO_2 Insertion S20

SV: Representative Kinetic Data Workup: Determination of k_1 Using ^{1}H NMR Spectroscopy S21

SVI: Control Experiments to Determine the Effect of Impurities on the Rate of CO_2 Insertion S23

SVII: Determination of [CO_2] by Quantitative $^{13}C\{^1H\}$ NMR Spectroscopy S24

SVIII: Reaction Order in CO_2 for the Insertion of CO_2 into (^{tBu}PBP)Pd(CH_2CH_3) S26

SIX: Eyring Analysis for the Insertion of CO_2 into (^{tBu}PBP)Pd(CH_2CH_3) in C_6D_6 S27

SX: NMR and IR Spectra of New Compounds S28

SXI: Computational Details S51

SXII: X-Ray Diffraction Data S53

SXIII: References S59

SI. Experimental Details

All experiments were performed under an N_2 atmosphere using either standard Schlenk techniques or an MBraun glovebox unless noted. Under standard operating procedures for glovebox use: purging was not performed between uses of pentane, benzene, and toluene. Solvents used in synthesis were deoxygenated by sparging with nitrogen and dried through an activated alumina column on an Innovative Technology Inc. system. Commercial chemicals were used as received. Anhydrous CO_2 was obtained from Airgas Inc. C_6D_6, toluene-d_8, and pyridine-d_5 were purchased from Cambridge Isotope Laboratories, Inc., degassed via three freeze-pump-thaw cycles and then dried using a plug of activated alumina and stored over molecular sieves. The pressure of CO_2 was measured with a Wallace & Tiernan 61B-1D-0800 Absolute Pressure Gauge. NMR spectra were recorded on Agilent DD2 -400, -500, -600 spectrometers at ambient probe temperatures unless otherwise specified. Chemical shifts for ^1H and $^{13}C\{^1H\}$ NMR spectra are reported in ppm and referenced to residual internal protio solvent. Chemical shifts for $^{31}P\{^1H\}$ NMR spectra are referenced using ^1H resonances based on relative gyromagnetic ratios of the nuclei.[1] IR spectra were collected on a Bruker Alpha FTIR spectrometer under 1 atm N_2. Mass spectrometric measurements were performed with a Thermo Fisher QExactive Orbitrap LC-MS system using continuous injection with a syringe. Samples were prepared in a glove box and loaded into a gas tight syringe, Hamilton 1750, for sample injection. The syringe and the PEEK capillaries to the ion source of the MS were cleaned with dry and oxygen-free solvents before sample injection. Samples were held at room temperature and continuously injected using a syringe pump at 50 μL/min. Electrospray was used for desolvatization and ionization, with the electrospray needle held at +3.5kV. Compressed air was used as desolvatization gas, capillary temperature was at 320 °C, probe heater temperature at 40 °C and sheath gas flow was at 5 L/min. Resolution was set to 35,000 M/ΔM. Mass spectra were recorded in the range of 150 to 750 m/z in positive ion mode. Measurements and data post-processing were performed with Thermo Xcalibur 4.1.31.9. (^{tBu}PBP)PdCl was synthesized according to a literature procedure.[2] New complexes were not characterized by elemental analysis due to the sensitivity of the samples to trace amounts of air or moisture. However, NMR spectra that demonstrate a high level of purity are provided as part of the supporting information.

SII. Synthesis and Characterization of New Compounds

(^{tBu}PBP)Pd(CH_2CH_3) (**1-Et**)

(^{tBu}PBP)PdCl (59.7 mg, 0.104 mmol) was dissolved in 5 mL of benzene and added to a 4-dram scintillation vial. 0.21 mL of a 0.5 M EtLi solution in a benzene/cyclohexane mixture (0.105 mmol, 1 equivalent) was added dropwise to the benzene solution. An immediate color change to orange was observed. The solution was allowed to stand for 1 hour and then was filtered through Celite to separate the salt precipitate from the desired product. The volatiles were removed from the filtrate under vacuum to give crude (^{tBu}PBP)Pd(CH_2CH_3) as a beige solid. The solid was then recrystallized twice in *n*-pentane at -35 °C to yield (^{tBu}PBP)Pd(CH_2CH_3) as colorless crystals (45.0 mg, 0.073 mmol, 76%) suitable for X-ray diffraction. Clean product can also be generated through the initial reaction of (^{tBu}PBP)PdCl with 2.0 M EtMgCl in THF followed by the same workup procedure.

^{1}H NMR (500 MHz, C_6D_6): δ 7.18 (dd, *J* = 5.7, 3.0 Hz, 2H, CH_{Ar}), 7.00 (dd, *J* = 5.8, 3.0 Hz, 2H, CH_{Ar}), 3.73 (vt, *J* = 2.3 Hz, 4H, CH_2), 2.09 (t, *J* = 8.1 Hz, 3H, Pd-CH_2CH_3), 1.49 (dt, *J* = 8.1, 5.3 Hz, 2H, Pd-CH_2CH_3), 1.23 (t, *J* = 6.7 Hz, 36H, C(CH_3)$_3$). ^{13}C{^{1}H} NMR (151 MHz, C_6D_6): δ 139.45 (t, *J* = 8.9 Hz, C_{Ar}), 118.56 (s, CH_{Ar}), 109.46 (s, CH_{Ar}), 42.77 (t, *J* = 14.6 Hz, CH_2), 35.94 (t, *J* = 5.7 Hz, *C*(CH_3)$_3$), 29.84 (t, *J* = 3.8 Hz, C(CH_3)$_3$), 19.22 (s, Pd-CH_2CH_3), -0.88 (br s, Pd-CH_2CH_3). ^{31}P{^{1}H} NMR (202 MHz, C_6D_6): δ 94.92 (s, ^{tBu}*P*B*P*).

At 65 °C (^{tBu}PBP)Pd(CH_2CH_3) starts to undergo β-hydride elimination, as evidenced by the presence of ethylene in the ^{1}H NMR spectrum, along with new peaks in the ^{31}P NMR spectrum.

(^{tBu}PBP)Pd($CH_2CH_2CH_3$) (**1-^{n}Pr**)

(^{tBu}PBP)PdCl (42.0 mg, 0.0730 mmol) was dissolved in 5 mL of benzene and added to a 4-dram scintillation vial. 0.04 mL of a 2.0 M ^{n}PrMgCl solution in diethyl ether (0.08 mmol, 1.1 equivalents) was added dropwise to the benzene solution. An immediate color change to yellow was observed. The solution was allowed to stand for 5 minutes and then was immediately filtered through Celite to separate the salt precipitate from the desired product. The volatiles were removed from the filtrate under vacuum to give crude (^{tBu}PBP)Pd($CH_2CH_2CH_3$) as a beige solid. The solid was then recrystallized twice in *n*-pentane at -35 °C to yield (^{tBu}PBP)Pd($CH_2CH_2CH_3$) as colorless crystals (25.1 mg, 0.0433 mmol, 59%) suitable for X-ray diffraction.

^{1}H NMR (500 MHz, C_6D_6): δ 7.17 (dd, *J* = 5.7, 3.2 Hz, 2H, CH_{Ar}), 7.00 (dd, *J* = 5.6, 3.3 Hz, 2H, CH_{Ar}), 3.73 (vt, *J* = 2.1 Hz, 4H, CH_2), 2.19-2.11 (m, 3H, Pd-$CH_2CH_2CH_3$), 1.62 (t, *J* = 7.0 Hz, 3H, Pd-$CH_2CH_2CH_3$), 1.44-1.38 (m, 3H, Pd-$CH_2CH_2CH_3$), 1.22 (t, *J* = 6.6 Hz, 36H, C(CH_3)$_3$). ^{13}C{^{1}H} NMR (151 MHz, C_6D_6): δ 139.43 (t, *J* = 8.7 Hz, C_{Ar}), 118.57 (s, CH_{Ar}), 109.46 (s, CH_{Ar}), 42.74 (t, *J* = 14.7 Hz, CH_2),

35.95 (t, J = 6.1 Hz, C(CH_3)$_3$), 29.81 (t, J = 3.8 Hz, C(CH_3)$_3$), 28.66 (s, Pd-$CH_2CH_2CH_3$), 24.92 (s, Pd-$CH_2CH_2CH_3$), 13.05 (br s, Pd-$CH_2CH_2CH_3$). $^{31}P\{^1H\}$ NMR (202 MHz, C_6D_6): δ 95.08 (s, ^{tBu}PBP).

At 60 °C (^{tBu}PBP)Pd($CH_2CH_2CH_3$) starts to undergo β-hydride elimination, as evidenced by the presence of propene in the ^{1}H NMR spectrum, along with new peaks in the ^{31}P NMR spectrum.

(^{tBu}PBP)Pd($CH_2C_6H_5$) (**1-Bn**)

(^{tBu}PBP)PdCl (50.4 mg, 0.0877 mmol) was dissolved in 5 mL of benzene and added to a 4-dram scintillation vial. 0.07 mL of a 1.32 M benzylMgCl solution in THF (0.092 mmol, 1.05 equivalents) was added dropwise to the benzene solution. An immediate color change to yellow was observed. The solution was allowed to stand for 1 hour and then was filtered through Celite to separate the salt precipitate from the desired product. The volatiles were removed from the filtrate under vacuum to give crude (^{tBu}PBP)Pd($CH_2C_6H_5$) as a yellow solid. The solid was then recrystallized twice in *n*-pentane at -35 °C to yield (^{tBu}PBP)Pd($CH_2C_6H_5$) as yellow crystals (30.6 mg, 0.0485 mmol, 55%) suitable for X-ray diffraction.

^{1}H NMR (500 MHz, C_6D_6): δ 7.43 (d, J = 7.5 Hz, 2H, Pd-$CH_2C_6H_5$), 7.28 (t, J = 7.6 Hz, 2H, Pd-$CH_2C_6H_5$), 7.15 (dd, J = 5.7, 3.2 Hz, 2H, CH_{Ar}), 6.96 (dd, J = 5.6, 3.2 Hz, 2H, CH_{Ar}), 6.90 (t, J = 7.3 Hz, 1H, Pd-$CH_2C_6H_5$), 3.67 (vt, J = 2.1 Hz, 4H, CH_2), 2.91 (vt, J = 3.6 Hz, 2H, Pd-$CH_2C_6H_5$), 1.11 (t, J = 6.7 Hz, 36H, C(CH_3)$_3$). $^{13}C\{^1H\}$ NMR (151 MHz, C_6D_6): δ 161.60 (t, J = 2.6 Hz, Pd-$CH_2C_6H_5$), 138.93 (t, J = 8.9 Hz, C_{Ar}), 128.06 (partially obscured by solvent, Pd-$CH_2C_6H_5$), 127.66 (s, Pd-$CH_2C_6H_5$), 118.78 (s, CH_{Ar}), 118.36 (s, Pd-$CH_2C_6H_5$), 109.41 (s, CH_{Ar}), 42.13 (t, J = 14.5 Hz, CH_2), 35.65 (t, J = 5.9 Hz, C(CH_3)$_3$), 29.62 (t, J = 3.6 Hz, C(CH_3)$_3$), 22.15 (t, J = 9.2 Hz, Pd-$CH_2C_6H_5$). $^{31}P\{^1H\}$ NMR (202 MHz, C_6D_6): δ 89.65 (s, ^{tBu}PBP).

Thermal decomposition of (^{tBu}PBP)Pd($CH_2C_6H_5$) to form ($^{tBu}PB^{Bn}P$)$_2$Pd$_2$ (**2-Bn**)

(^{tBu}PBP)Pd($CH_2C_6H_5$) (5.5 mg, 0.0087 mmol) was dissolved in 0.5 mL of C_6D_6 and added to a J-Young NMR tube. The tube was heated at 65 °C in an oil bath for 3 days and was periodically monitored by NMR spectroscopy. After 3 days, full conversion from the starting material was achieved, and a major product at 41.60 ppm, which integrated to 90% of the resonances in the $^{31}P\{^1H\}$ NMR spectrum was observed. The NMR tube was laid sideways at room temperature for 2 days, where colorless crystals suitable for X-ray diffraction crashed out of solution. XRD studies of these crystals gave the structure of ($^{tBu}PB^{Bn}P$)$_2$Pd$_2$. The major peaks in the ^{1}H NMR spectrum are consistent with the structure of the dimer.

^{1}H NMR (400 MHz, C_6D_6): δ 7.79 (dd, J = 5.8, 3.2 Hz, 4H, CH_{Ar}), 7.14-7.03 (m, 8H, CH_{Ar} (4H) and CH_{Ar} (4H)), 7.02-6.96 (m, 6H, CH_{Ar} (4H) and CH_{Ar} (2H)), 4.18 (dt, J = 14.8, 2.7 Hz, 4H, CH_2), 3.62 (dt, J = 14.8, 3.1 Hz, 4H, CH_2), 3.00 (s, 4H, CH_2), 1.45 (t, J = 6.0 Hz, 36H, C(CH_3)$_3$), 1.41 (t, J = 5.8 Hz, 36H, C(CH_3)$_3$). $^{31}P\{^1H\}$ NMR (162 MHz, C_6D_6): δ 41.60 (s, ($^{tBu}PB^{Bn}P$)$_2$Pd$_2$).

(^{tBu}PBP)Pd(CH_2-4-OMe-C_6H_4) (**1-^{OMe}Bn**)

(^{tBu}PBP)PdCl (50.9 mg, 0.0885 mmol) was dissolved in 5 mL of benzene and added to a 4-dram scintillation vial. 0.4 mL of a 0.25 M 4-methoxy-benzylMgCl solution in THF (0.092 mmol, 1.05 equivalents) was added dropwise to the benzene solution. An immediate color change to yellow was observed. The solution was allowed to stand for 1 hour and then was filtered through Celite to separate the salt precipitate from the desired product. The volatiles were removed from the filtrate under vacuum to give crude (^{tBu}PBP)Pd(CH_2-4-OMe-C_6H_4) as a yellow solid. The solid was then recrystallized twice in *n*-pentane at -35 °C to yield (^{tBu}PBP)Pd(CH_2-4-OMe-C_6H_4) as yellow crystals (38.8 mg, 0.0587 mmol, 66%) suitable for X-ray diffraction.

^{1}H NMR (500 MHz, C_6D_6): δ 7.34 (d, *J* = 8.7 Hz, 2H, Pd-$CH_2C_6\mathit{H}_4OCH_3$), 7.15 (dd, *J* = 5.7, 3.2 Hz, 2H, $C\mathit{H}_{Ar}$), 6.97 (dd, *J* = 5.6 Hz, 3.2 Hz, 2H, $C\mathit{H}_{Ar}$), 6.94 (d, *J* = 8.6 Hz, 2H, Pd-$CH_2C_6\mathit{H}_4OCH_3$), 3.69 (vt, *J* = 2.1 Hz, 4H, $C\mathit{H}_2$), 3.55 (s, 3H, $OC\mathit{H}_3$), 2.83 (t, *J* = 3.8 Hz, 2H, Pd-$C\mathit{H}_2C_6H_4OCH_3$), 1.12 (t, *J* = 6.7 Hz, 36H, $C(C\mathit{H}_3)_3$). ^{13}C{^{1}H} NMR (151 MHz, C_6D_6): δ 153.99 (s, Pd-$CH_2\mathit{C}_6H_4OCH_3$), 153.09 (t, *J* = 2.3 Hz, Pd-$CH_2\mathit{C}_6H_4OCH_3$), 139.02 (t, *J* = 8.7 Hz, C_{Ar}), 128.42 (s, Pd-$CH_2\mathit{C}_6H_4OCH_3$), 118.74 (s, $\mathit{C}H_{Ar}$), 113.92 (s, Pd-$CH_2\mathit{C}_6H_4OCH_3$), 109.41 (s, $\mathit{C}H_{Ar}$), 55.35 (s, $O\mathit{C}H_3$), 42.26 (t, *J* = 14.7 Hz, $\mathit{C}H_2$), 35.67 (t, *J* = 6.1 Hz, $\mathit{C}(CH_3)_3$), 29.65 (t, *J* = 3.8 Hz, $C(\mathit{C}H_3)_3$), 19.84 (t, *J* = 8.4 Hz, Pd-$\mathit{C}H_2C_6H_4OCH_3$). ^{31}P{^{1}H} NMR (202 MHz, C_6D_6): δ 90.15 (s, ^{tBu}*P*B*P*).

Thermal decomposition of (^{tBu}PBP)Pd(CH_2-4-OMe-C_6H_4) to form (^{tBu}PB^{OMeBn}P)$_2$Pd$_2$ (**2-^{OMe}Bn**)

(^{tBu}PBP)Pd(CH_2-4-OMe-C_6H_4) (4.8 mg, 0.0073 mmol) was dissolved in 0.5 mL of C_6D_6 and added to a J-Young NMR tube. The tube was heated at 65 °C in an oil bath for 3 days and was periodically monitored by NMR spectroscopy. After 3 days, full conversion from the starting material was achieved, and a major product at 41.66 ppm, which integrated to 96% of the resonances in the ^{31}P{^{1}H} NMR spectrum was observed. These peaks are similar to what was observed in the thermal decomposition of (^{tBu}PBP)Pd($CH_2C_6H_5$), suggesting that the new decomposition product is the Pd(0) dimer, (^{tBu}PB^{OMeBn}P)$_2$Pd$_2$.

^{1}H NMR (400 MHz, C_6D_6): δ 7.82 (dd, *J* = 5.7, 3.3 Hz, 4H, $C\mathit{H}_{Ar}$), 7.06-6.99 (m, 8H, $C\mathit{H}_{Ar}$ (4H) and $C\mathit{H}_{Ar}$ (4H)), 6.77-6.64 (m, 4H, $C\mathit{H}_{Ar}$), 4.23 (dt, *J* = 14.8, 2.7 Hz, 4H, $C\mathit{H}_2$), 3.67 (dt, *J* = 14.8, 2.9 Hz, 4H, $C\mathit{H}_2$), 3.27 (s, 6H, $OC\mathit{H}_3$), 2.98 (s, 4H, $C\mathit{H}_2$), 1.47 (t, *J* = 6.0 Hz, 36H, $C(C\mathit{H}_3)_3$), 1.42 (t, *J* = 5.8 Hz, 36H, $C(C\mathit{H}_3)_3$). ^{31}P{^{1}H} NMR (162 MHz, C_6D_6): δ 41.66 (s, (^{tBu}*P*B^{OMeBn}*P)*$_2$Pd$_2$).

(^{tBu}PBP)Pd(C_6H_5) (**1-Ph**)

Route A: (^{tBu}PBP)PdCl (25.7 mg, 0.0447 mmol) was dissolved in 5 mL of benzene and added to a 4-dram scintillation vial. 0.15 mL of a 3.0 M PhMgBr solution in diethyl ether (0.045 mmol, 1 equivalent) was

added dropwise to the benzene solution. The solution was allowed to stand for 2 days and then was filtered through Celite to separate the salt precipitate from the reaction mixture. The volatiles were removed from the filtrate under vacuum to give a mixture of the starting material (^{tBu}PBP)PdCl, and the desired product (^{tBu}PBP)Pd(C_6H_5). A stoichiometric amount of PhMgBr, based on NMR integrations, was used to repeat the above procedure until only the desired product remained (typically 3 total Grignard additions). This may be necessary due to the tendency of these products to back convert to (^{tBu}PBP)PdCl upon reaction with the halide salt, which has been previously observed.[2] The solid was then recrystallized twice in *n*-pentane at -35 °C to yield (^{tBu}PBP)Pd(C_6H_5) as colorless crystals (14.1 mg, 0.0229 mmol, 45%) suitable for X-ray diffraction.

^{1}H NMR (500 MHz, C_6D_6): δ 8.12 (d, *J* = 7.1 Hz, 2H, Pd-C_6H_5), 7.45 (t, *J* = 7.3 Hz, 2H, Pd-C_6H_5), 7.19-7.15 (m, 3H, Pd-C_6H_5 (1H) and CH_{Ar} (2H)), 7.01 (dd, *J* = 5.7, 3.2 Hz, 2H, CH_{Ar}), 3.74 (vt, *J* = 2.1 Hz, 4H, CH_2), 1.12 (t, *J* = 6.8 Hz, 36H, C(CH_3)$_3$). ^{13}C{^{1}H} NMR (151 MHz, C_6D_6): δ 143.74 (s, Pd-C_6H_5), 139.39 (t, *J* = 8.7 Hz, C_{Ar}), 128.06 (partially obscured by solvent, Pd-C_6H_5), 125.95 (s, Pd-C_6H_5), 121.51 (s, Pd-C_6H_5), 118.76 (s, CH_{Ar}). 109.53 (s, CH_{Ar}), 42.26 (t, *J* = 14.7 Hz, CH_2), 36.05 (t, *J* = 6.4 Hz, *C*(CH_3)$_3$), 29.71 (t, *J* = 3.8 Hz, C(CH_3)$_3$). ^{31}P{^{1}H} NMR (202 MHz, C_6D_6): δ 94.94 (s, ^{tBu}*P*B*P*).

Route B: (^{tBu}PBP)PdCl (11.1 mg, 0.0193 mmol) was dissolved in 0.5 mL of benzene-d_6 and added to a J-Young NMR tube. 0.02 mL of a 1.0 M PhMgBr solution in THF (0.02 mmol, 1.04 equivalent) was added dropwise to the benzene solution. 0.05 mL of degassed 1,4-dioxane was added to the J-Young tube and a white precipitate was immediately observed. The solution was allowed to stand for 3 days at room temperature and the reaction progress was monitored periodically by ^{31}P{^{1}H} NMR spectroscopy until full conversion was observed. The reaction mixture was filtered through a plug of Celite and the volatiles were removed under vacuum. NMR spectroscopy confirmed the remaining solid to be pure (^{tBu}PBP)Pd(C_6H_5) (5.7mg, 0.0093 mmol, 48%) with no evidence of back conversion. This well precedented used of 1,4-dioxane may possibly be extended to the syntheses of the other Pd alkyl species.

(^{tBu}PBP)Pd{OC(O)CH_2CH_3} (**3-Et**)

(^{tBu}PBP)Pd(CH_2CH_3) (15.0 mg, 0.0264 mmol) was dissolved in 500 µL of C_6D_6 and added to a J-Young NMR tube. The solution was degassed via three freeze-pump-thaw cycles and 1 atm CO_2 was added via a Schlenk line. The solution was allowed to stand for 5 hours at room temperature. The volatiles and excess CO_2 were removed under vacuum and the complex was washed with 1 x 2 mL cold *n*-pentane to yield the product, (^{tBu}PBP)Pd{OC(O)CH_2CH_3} as a beige solid (15.8 mg, 0.0258 mmol, 97.5%). Crystals suitable for X-ray diffraction were grown from *n*-pentane at -35 °C.

^{1}H NMR (500 MHz, C_6D_6): δ 7.14 (dd, *J* = 5.6, 3.2 Hz, 2H, CH_{Ar}), 6.92 (dd, *J* = 5.7, 3.2 Hz, 2H, CH_{Ar}), 3.55 (vt, *J* = 2.3 Hz, 4H, CH_2), 2.66 (q, *J* = 7.6 Hz, 2H, CH_2CH$_3$), 1.51 (t, *J* = 7.6 Hz, 3H, CH$_2$CH_3), 1.28 (t, *J* = 7.0 Hz, 36H, C(CH_3)$_3$). $^{13}C\{^{1}H\}$ NMR (151 MHz, C_6D_6): 177.77 (s, Pd-O*C*(O)CH$_2$CH$_3$), 138.44 (t, *J* = 9.0 Hz, C_{Ar}), 118.65 (s, *C*H$_{Ar}$), 108.84 (s, *C*H$_{Ar}$), 39.20 (t, *J* = 14.4 Hz, *C*H$_2$), 34.96 (t, *J* = 6.1 Hz, *C*(CH$_3$)$_3$), 32.02 (s, Pd-OC(O)*C*H$_2$CH$_3$), 28.97 (t, *J* = 3.8 Hz, C(*C*H$_3$)$_3$), 11.55 (s, Pd-OC(O)CH$_2$*C*H$_3$). δ $^{31}P\{^{1}H\}$ NMR (202 MHz, C_6D_6): δ 87.03 (s, ^{tBu}*P*B*P*). IR (Diamond ATR cell, cm^{-1}): 1591 (νCO_2), 1288 (νCO_2).

(^{tBu}PBP)Pd{OC(O)CH$_2$CH$_2$CH$_3$} (**3-^{n}Pr**)

(^{tBu}PBP)Pd(CH$_2$CH$_2$CH$_3$) (2.2 mg, 0.0038 mmol) was dissolved in 500 μL of C_6D_6 and added to a J-Young NMR tube. The solution was degassed via three freeze-pump-thaw cycles and 1 atm CO_2 was added via a Schlenk line. The solution was allowed to stand for 3 days at room temperature. The volatiles and excess CO_2 were removed under vacuum to yield the product, (^{tBu}PBP)Pd{OC(O)CH$_2$CH$_2$CH$_3$} as a beige solid (2.3 mg, 0.0037 mmol, 97%).

^{1}H NMR (400 MHz, C_6D_6): δ 7.14 (dd, *J* = 5.8, 3.2 Hz, 2H, CH_{Ar}), 6.92 (dd, *J* = 5.7, 3.2 Hz, 2H, CH_{Ar}), 3.55 (vt, *J* = 2.3 Hz, 4H, CH_2), 2.66 (t, *J* = 7.3 Hz, 2H, CH_2CH$_2$CH$_3$), 2.08 (h, *J* = 7.3 Hz, 2H, CH$_2$CH_2CH$_3$), 1.28 (t, *J* = 7.0 Hz, 36H, C(CH_3)$_3$), 1.20 (t, *J* = 7.4 Hz, 3H, CH$_2$CH$_2$CH_3). $^{13}C\{^{1}H\}$ NMR (151 MHz, C_6D_6): δ 177.46 (s, Pd-O*C*(O)CH$_2$CH$_2$CH$_3$), 138.85 (t, *J* = 9.0 Hz, C_{Ar}), 119.07 (s, *C*H$_{Ar}$), 109.25 (s, *C*H$_{Ar}$), 41.65 (s, Pd-OC(O)CH$_2$*C*H$_2$CH$_3$), 39.61 (t, *J* = 14.4 Hz, *C*H$_2$), 35.35 (t, *J* = 6.1 Hz, *C*(CH$_3$)$_3$), 29.40 (t, *J* = 3.8 Hz, C(*C*H$_3$)$_3$), 20.89 (s, Pd-OC(O)*C*H$_2$CH$_2$CH$_3$), 15.16 (s, Pd-OC(O)CH$_2$CH$_2$*C*H$_3$). $^{31}P\{^{1}H\}$ NMR (162 MHz, C_6D_6): δ 87.01 (s, ^{tBu}*P*B*P*). IR (Diamond ATR cell, cm^{-1}): 1590 (νCO_2), 1289 (νCO_2).

(^{tBu}PBP)Pd{OC(O)CH$_2$C$_6$H$_5$} (**3-Bn**)

(^{tBu}PBP)Pd(CH$_2$C$_6$H$_5$) (6.1 mg, 0.0097 mmol) was dissolved in 500 μL of C_6D_6 and added to a J-Young NMR tube. The solution was degassed via three freeze-pump-thaw cycles and 1 atm CO_2 was added via a Schlenk line. The solution was allowed to stand for 5 days at room temperature. The volatiles and excess CO_2 were removed under vacuum and the complex was washed with 1 x 2 mL cold *n*-pentane to yield the product, (^{tBu}PBP)Pd{OC(O)CH$_2$C$_6$H$_5$} as a yellow solid (5.9 mg, 0.0087 mmol, 91%).

^{1}H NMR (500 MHz, C_6D_6): δ 7.67 (d, *J* = 7.5 Hz, 2H, CH$_2$C$_6$$H_5$), 7.27 (t, *J* = 7.6 Hz, 2H, CH$_2$C$_6$$H_5$), 7.14-7.10 (m, 3H, CH$_2C_6$$H_5$ (1H) and CH_{Ar} (2H)), 6.90 (dd, *J* = 5.7, 3.2 Hz, 2H, CH_{Ar}), 3.39 (s, 2H, CH_2C$_6$H$_5$), 3.52 (vt, *J* = 2.3 Hz, 4H, CH_2), 1.20 (t, *J* = 7.0 Hz, 36H, C(CH_3)$_3$). $^{13}C\{^{1}H\}$ NMR (151 MHz, C_6D_6): δ 174.66 (s, Pd-O*C*(O)), 140.23 (s, CH$_2$*C*$_6$H$_5$), 138.80 (t, *J* = 9.2 Hz, C_{Ar}), 130.37 (s, CH$_2$*C*$_6$H$_5$), 128.06 (partially obscured by solvent, CH$_2$C$_6$H$_5$), 125.41 (s, CH$_2$*C*$_6$H$_5$), 119.08 (s, *C*H$_{Ar}$), 109.27 (s, *C*H$_{Ar}$), 47.38 (s, *C*H$_2$C$_6$H$_5$), 39.53 (t, *J* = 14.4 Hz, *C*H$_2$), 35.27 (t, *J* = 6.1 Hz, *C*(CH$_3$)$_3$), 29.36 (t, *J* = 3.8 Hz, C(*C*H$_3$)$_3$).

$^{31}P\{^1H\}$ NMR (202 MHz, C_6D_6): δ 86.96 (s, ^{tBu}*P*B*P*). IR (Diamond ATR cell, cm^{-1}): 1601 (νCO_2), 1290 (νCO_2).

(^{tBu}PBP)Pd{OC(O)CH_2-4-OMe-C_6H_4} (**3-^{OMe}Bn**)

(^{tBu}PBP)Pd($CH_2C_6H_4OCH_3$) (7.7 mg, 0.012 mmol) was dissolved in 500 µL of C_6D_6 and added to a J-Young NMR tube. The solution was degassed via three freeze-pump-thaw cycles and 1 atm CO_2 was added via a Schlenk line. The solution was allowed to stand for 7 days at room temperature. The volatiles and excess CO_2 were removed under vacuum and the complex was washed with 1 x 2 mL cold *n*-pentane to yield the product, (^{tBu}PBP)Pd{OC(O)CH_2-4-OMe-C_6H_4} as a yellow solid (6.9 mg, 0.0098 mmol, 84%).

^{1}H NMR (400 MHz, C_6D_6): δ 7.61 (d, *J* = 8.7 Hz, 2H, $CH_2C_6H_4OCH_3$), 7.13 (dd, *J* = 5.7, 3.2 Hz, 2H, CH_{Ar}), 6.97-6.81 (m, 4H, $CH_2C_6H_4OCH_3$ (2H) and CH_{Ar} (2H)), 3.92 (s, 2H, $CH_2C_6H_4OCH_3$), 3.52 (vt, *J* = 2.3 Hz, 4H, CH_2), 3.39 (s, 3H, OCH_3), 1.21 (t, *J* = 7.0 Hz, 36H, $C(CH_3)_3$). $^{13}C\{^1H\}$ NMR (151 MHz, C_6D_6): δ 175.17 (s, Pd-O*C*(O)), 158.23 (s, $CH_2C_6H_4OCH_3$), 138.81 (t, *J* = 9.0 Hz, C_{Ar}), 132.48 (s, $CH_2C_6H_4OCH_3$), 131.15 (s, $CH_2C_6H_4OCH_3$), 119.07 (s, CH_{Ar}), 113.47 (s, $CH_2C_6H_4OCH_3$), 109.26 (s, CH_{Ar}), 54.80 (s, OCH_3), 46.35 (s, $CH_2C_6H_4OCH_3$), 39.55 (t, *J* = 14.4 Hz, CH_2), 35.29 (t, *J* = 6.1 Hz, $C(CH_3)_3$), 29.35 (t, *J* = 3.8 Hz, $C(CH_3)_3$). $^{31}P\{^1H\}$ NMR (162 MHz, C_6D_6): δ 86.97 (s, ^{tBu}*P*B*P*). IR (Diamond ATR cell, cm^{-1}): 1602 (νCO_2), 1290 (νCO_2).

Thermal decomposition of (^{tBu}PBP)Pd(CH_3) (**1-Me**) to form ($^{tBu}PB^{Me}P)_2Pd_2$ (**2-Me**)

(^{tBu}PBP)Pd(CH_3) was synthesized via our previously reported route.[2] (^{tBu}PBP)Pd(CH_3) (8.0 mg, 0.014 mmol) was dissolved in 0.5 mL of C_6D_6 and added to a J-Young NMR tube. The tube was heated at 65 °C in an oil bath for one week and was periodically monitored by NMR spectroscopy. Slow decomposition is observed, where after 5 days there is 10% decomposition to ($^{tBu}PB^{Me}P)_2Pd_2$ (**2-Me**) (66.7 ppm, 10% by integration) based on the $^{31}P\{^1H\}$ NMR spectrum. ($^{tBu}PB^{Me}P)_2Pd_2$ is assigned based on the similar chemical shift in the $^{31}P\{^1H\}$ NMR spectrum to **2-Bn** and **2-H** (see Figure S55).

SIII. Attempted Syntheses of (^{tBu}PBP)Pd-C$_{(alkyl)}$ and Related Complexes

Reaction of (^{tBu}PBP)PdCl and ^{t}BuMgCl

(^{tBu}PBP)PdCl (6.9 mg, 0.012 mmol) was dissolved in 0.5 mL of C_6D_6 and added to a J-Young NMR tube. 0.05 mL of a 1.0 M ^{t}BuMgCl solution in THF (0.05 mmol, 4.2 equivalents) was added dropwise to the C_6D_6 solution. The reaction proceeds slowly with 1.1 equivalents of Grignard reagent, so a larger excess was used to reduce the reaction time. The same products are observed with 1.1 equivalents and 4.2 equivalents, however, even with 4.2 equivalents the reaction still takes hours, compared to just minutes for the other Pd-C(alkyl) species. ^{t}BuMgCl was used instead of ^{t}BuLi as a safety precaution. An immediate color change from clear to light yellow was observed. The reaction mixture became a more intense and darker yellow over time. An NMR spectrum recorded 20 minutes after the reaction started showed five resonances in the $^{31}P\{^{1}H\}$ NMR spectrum. The major peak at 87.5 ppm (40% by integration) corresponds to the (^{tBu}PBP)PdCl starting material. Although (^{tBu}PBP)Pd(C(CH$_3$)$_3$) is not observed in the ^{1}H NMR spectrum, the peaks at 96.4 ppm (34%) and 114.9 ppm (21%) are proposed to be associated with an unknown product (96.4 ppm) and (^{tBu}PBP)PdH (114.9), the β-hydride elimination product of (^{tBu}PBP)Pd(C(CH$_3$)$_3$). Consistent with this, isobutene is observed in the ^{1}H NMR spectrum (δ 4.73 (p, J = 1.3 Hz, 2H) and 1.59 (t, J = 1.3 Hz, 4H)), further supporting rapid β-hydride elimination of the transient (^{tBu}PBP)Pd(C(CH$_3$)$_3$) product. (^{tBu}PBP)PdH then undergoes an unusual dimerization to form a bridging Pd(0) dimeric species, (^{tBu}PB^{H}P)$_2$Pd$_2$ (63.4 ppm, 4%, **2-H**, *vide infra*). There is also a small peak at 16.3 ppm (1%), which may correspond to ^{tBu}PB^{tBu}P, the reductive elimination product directly from (^{tBu}PBP)Pd(C(CH$_3$)$_3$). The peak associated with the putative (^{tBu}PBP)PdH species converts to (^{tBu}PB^{H}P)$_2$Pd$_2$ over time, while the unknown peak at 96.4 ppm remains present.

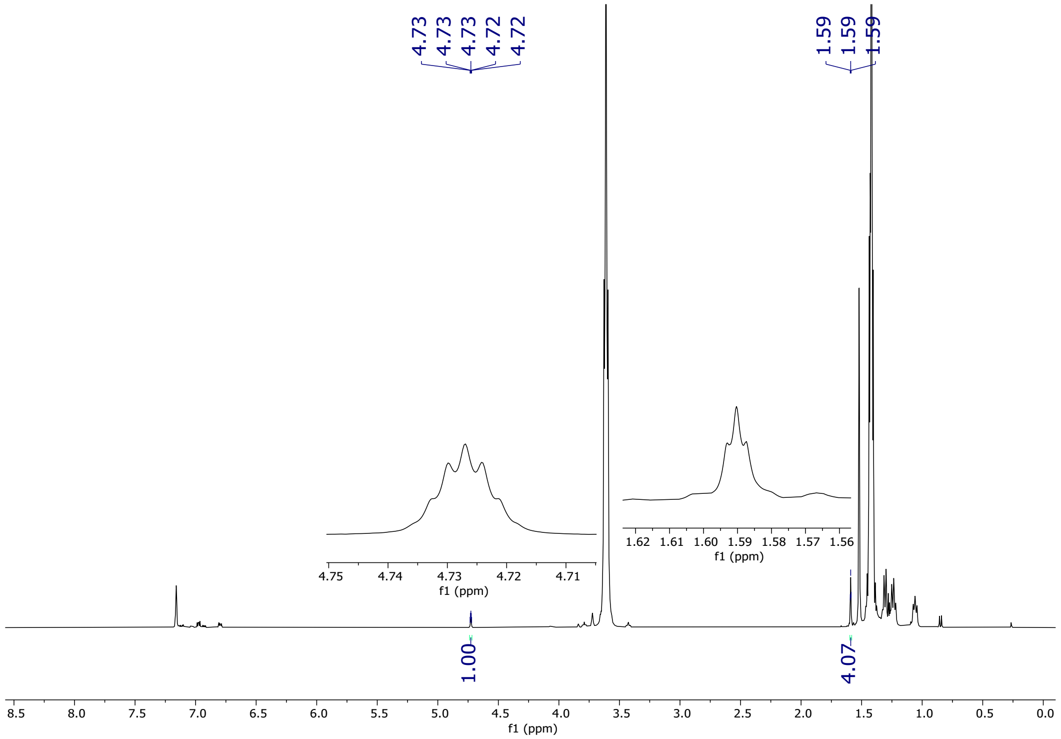

Figure S1. ^{1}H NMR spectrum of the crude reaction mixture after 20 minutes at room temperature in C_6D_6. The peaks corresponding to isobutene are picked, integrated, and enlarged. The peak at 1.59 ppm integrates slightly higher than expected due to overlap with the larger signals upfield of it.

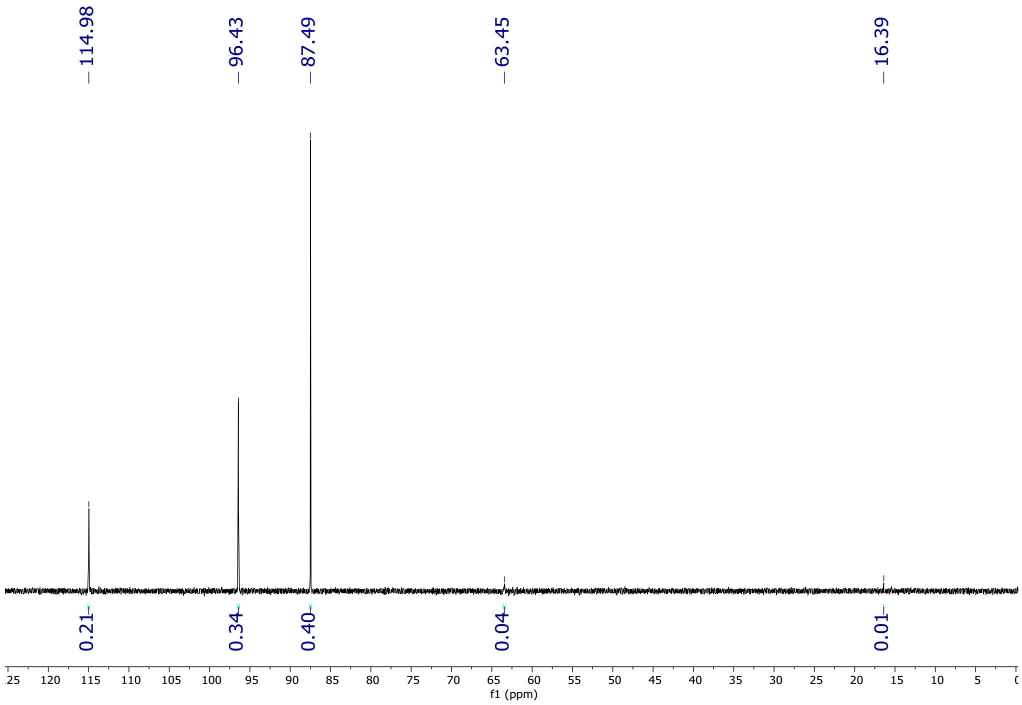

Figure S2. $^{31}P\{^{1}H\}$ NMR spectrum of the crude reaction mixture after 20 minutes at room temperature in C_6D_6.

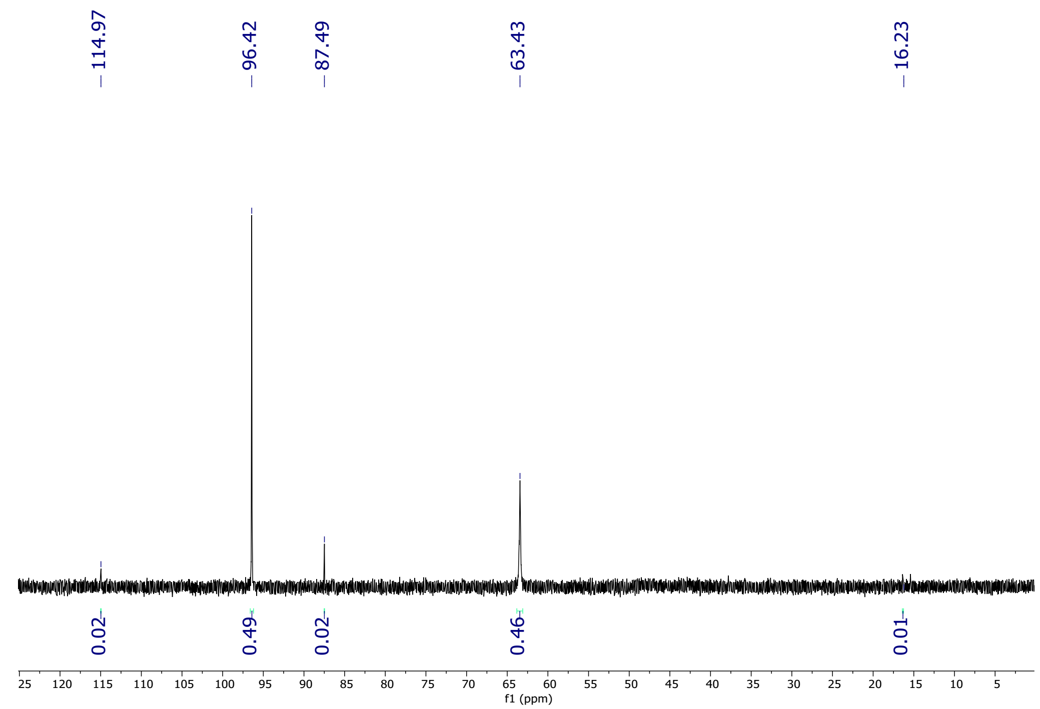

Figure S3. $^{31}P\{^1H\}$ NMR spectrum of the crude reaction mixture after 3 hours at room temperature in C_6D_6. The (^{tBu}PBP)PdCl starting material (87.49 ppm, 2%) is nearly depleted, and the two major products are an unknown species at 96.42 ppm (49%) and a product consistent with decomposition of a palladium hydride, which presumably forms via β-hydride elimination from the transient (^{tBu}PBP)Pd($C(CH_3)_3$) species (63.43 ppm, 46%).

<u>Reaction of (^{tBu}PBP)PdCl and ^{i}PrLi</u>

(^{tBu}PBP)PdCl (5.0 mg, 0.0087 mmol) was dissolved in 0.5 mL of C_6D_6 and added to a J-Young NMR tube. 0.02 mL of a 0.7 M ^{i}PrLi solution in pentane (0.014 mmol, 1.6 equivalents) was added dropwise to the C_6D_6 solution. An immediate color change from clear to yellow was observed. The solution further changed color to orange and then to black over the course of 3 minutes. A black solid, presumably palladium black, precipitated out of solution. A $^{31}P\{^1H\}$ NMR spectrum recorded 10 minutes after the reaction started contained a single resonance assigned to ^{tBu}PB^{iPr}P. Peaks consistent with ^{tBu}PB^{iPr}P are also observed in the ^{1}H NMR spectrum. Isolation of this product was achieved by filtration through a plug of Celite and subsequent removal of the volatiles under vacuum. HRMS was consistent with the formation of ^{tBu}PB^{iPr}P.

^{1}H NMR (400 MHz, C_6D_6): δ 7.75 (dd, J = 5.9, 3.2 Hz, 2H, CH_{Ar}), 7.18 (dd, J = 5.8, 3.2 Hz, 2H, CH_{Ar}), 4.08 (d, J = 3.8 Hz, 4H, CH_2), 2.29 (p, J = 7.7 Hz, 1H, B-$CH(CH_3)_2$), 1.56 (p, J = 7.4 Hz, 6H, B-$CH(CH_3)_2$), 1.09 (d, J = 10.4 Hz, 36H, $C(CH_3)_3$). $^{31}P\{^1H\}$ NMR (162 MHz, C_6D_6): δ 15.62 (s, ^{tBu}*P*B^{iPr}*P*). HRMS (ESI$^+$): 477.3687 [^{tBu}PB^{iPr}P+H]$^+$. Calc for [$C_{27}H_{52}BN_2P_2$]: 477.3693.

NMR spectra for ^{tBu}PB^{iPr}P at 25 °C in C_6D_6 are shown in Figures S4-S5.

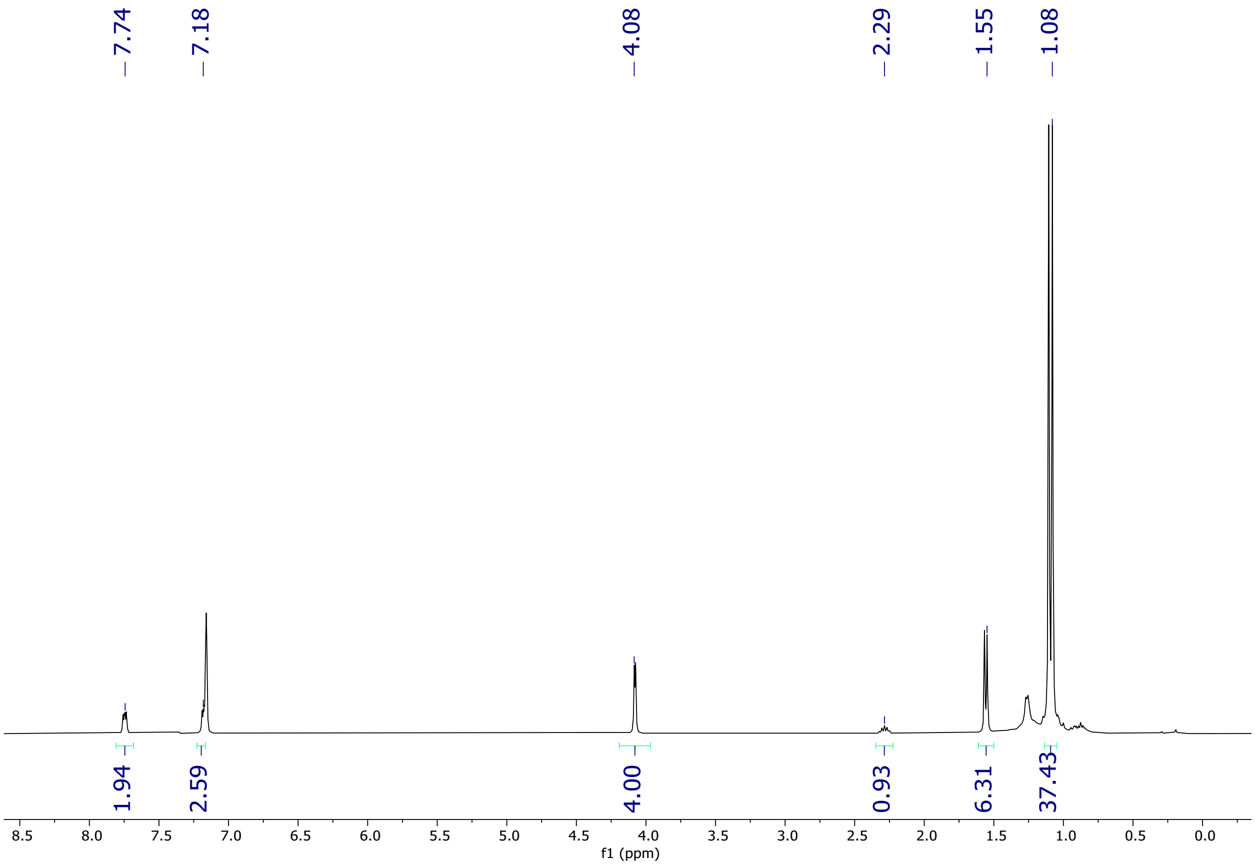

Figure S4. ^{1}H NMR spectrum of $^{tBu}PB^{iPr}P$ in C_6D_6 at room temperature.

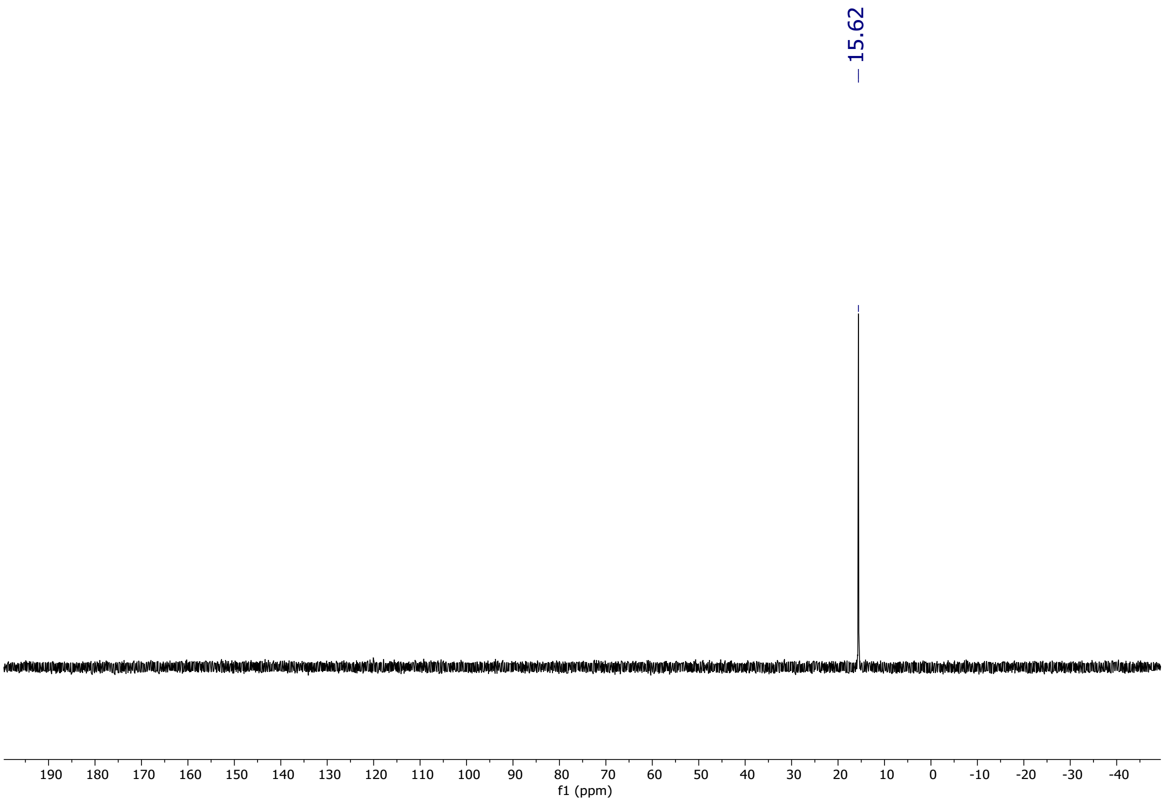

Figure S5. $^{31}P\{^1H\}$ NMR spectrum of $^{tBu}PB^{iPr}P$ in C_6D_6 at room temperature.

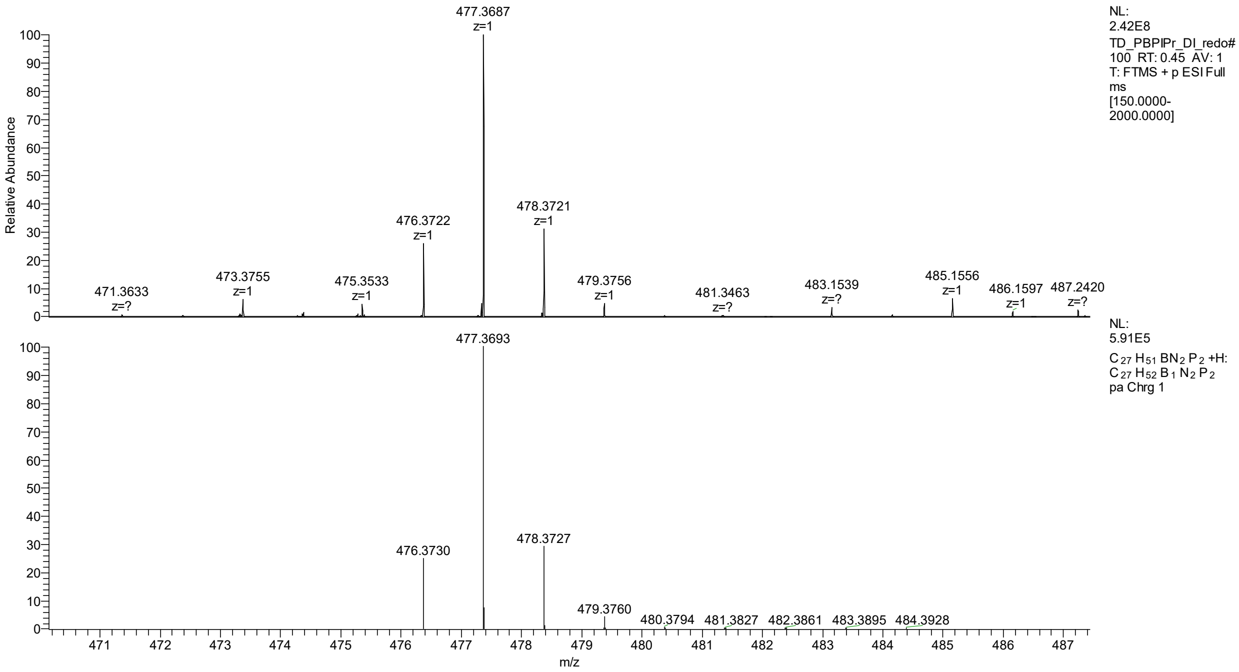

Figure S6. HRMS (ESI+) of $^{tBu}PB^{iPr}P$.

Reaction of (^{tBu}PBP)PdCl and ^{n}BuLi

(^{tBu}PBP)PdCl (10.3 mg, 0.018 mmol) was dissolved in 0.5 mL of C_6D_6 and added to a J-Young NMR tube. 0.01 mL of a 2.5 M ^{n}BuLi solution in hexanes (0.025 mmol, 1.4 equivalents) was added dropwise to the C_6D_6 solution. An immediate color change from clear to light yellow was observed. Two resonances were present in the $^{31}P\{^1H\}$ NMR spectrum recorded 10 minutes after the start of the reaction. The major peak at 94.96 ppm (97% by integration) corresponds to (^{tBu}PBP)Pd($CH_2CH_2CH_2CH_3$), and the minor peak (3% by integration) is the reductively coupled product, ^{tBu}PB^{nBu}P. Although the peak for the CH_2 directly bound to Pd is covered by the hexane solvent peaks, all other peaks for (^{tBu}PBP)Pd($CH_2CH_2CH_2CH_3$) were located in the ^{1}H NMR spectrum. Isolation of the major product was not possible and a small amount of ^{tBu}PB^{nBu}P is present even when the reaction is performed at -35 °C in toluene-d_8. The conversion of (^{tBu}PBP)Pd($CH_2CH_2CH_2CH_3$) to ^{tBu}PB^{nBu}P continues over time.

For (^{tBu}PBP)Pd($CH_2CH_2CH_2CH_3$): ^{1}H NMR (400 MHz, C_6D_6): δ 7.15 (dd, J = 5.7, 3.2 Hz, 2H, CH_{Ar}), 6.99 (dd, J = 5.6, 3.2 Hz, 2H, CH_{Ar}), 3.73 (vt, J = 2.0 Hz, 4H, CH_2), 2.14-2.06 (m, 2H, Pd-$CH_2CH_2CH_2CH_3$), 1.94-1.74 (m, 5H, Pd-$CH_2CH_2CH_2CH_3$ (2H) and Pd-$CH_2CH_2CH_2CH_3$ (3H)), 1.22 (t, J = 6.7 Hz, C(CH_3)$_3$). $^{31}P\{^1H\}$ NMR (162 MHz, C_6D_6): δ 94.96 (s, ^{tBu}*P*B*P*).

For ^{tBu}PB^{nBu}P: $^{31}P\{^1H\}$ NMR (162 MHz, C_6D_6): δ 14.54 (s, ^{tBu}*P*B^{nBu}*P*).

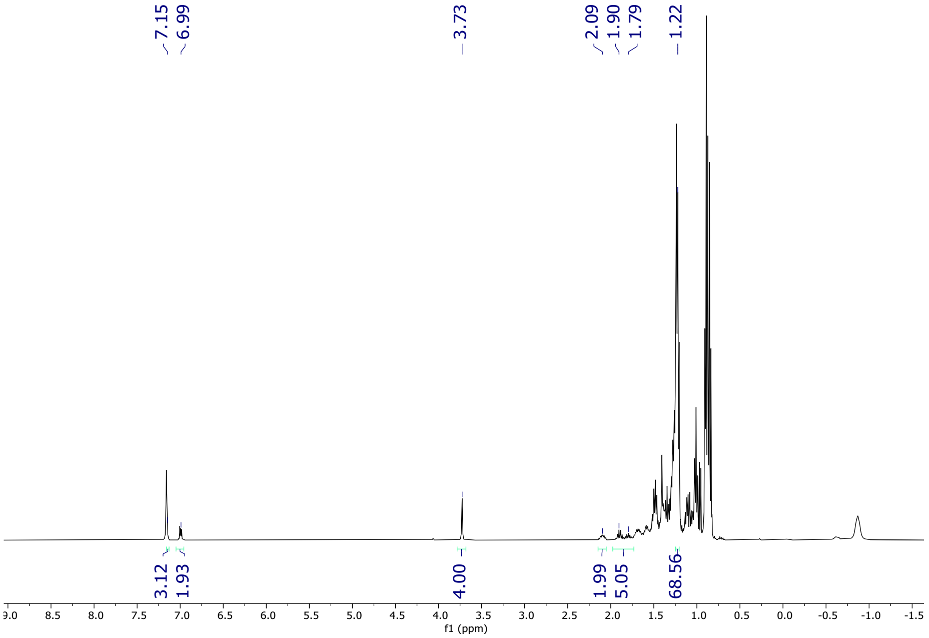

Figure S7. ^{1}H NMR of the crude reaction mixture of (^{tBu}PBP)PdCl with ^{n}BuLi in C_6D_6 after 10 minutes at room temperature. Peaks corresponding to the major product, (^{tBu}PBP)Pd($CH_2CH_2CH_2CH_3$), are picked and integrated.

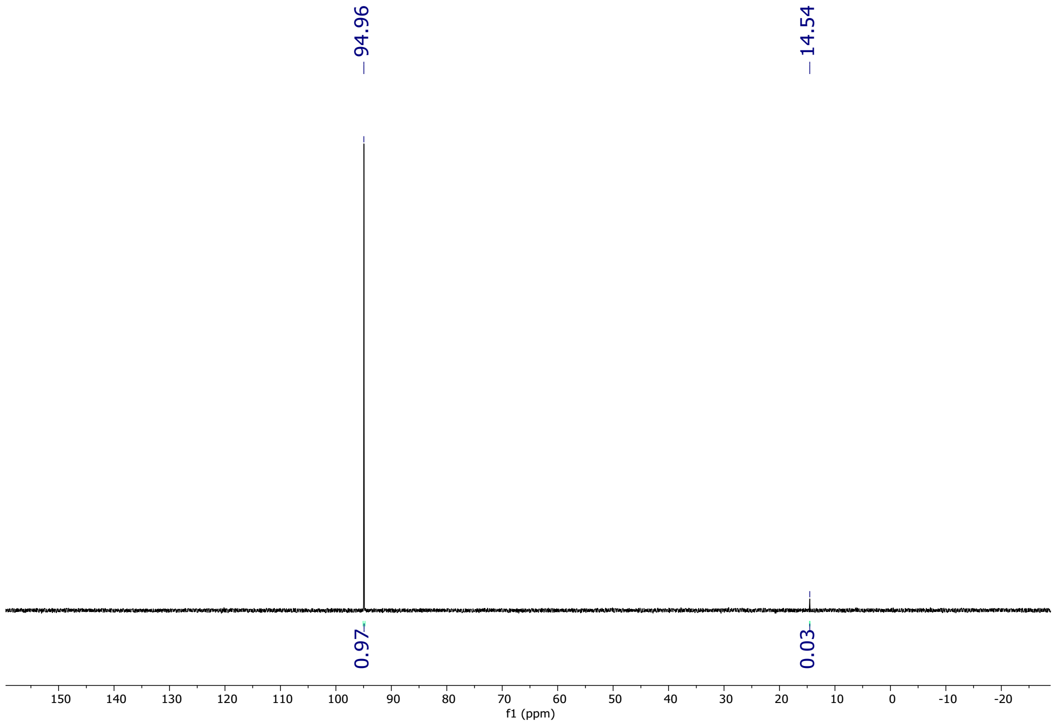

Figure S8. $^{31}P\{^{1}H\}$ NMR of the crude reaction mixture of (^{tBu}PBP)PdCl with ^{n}BuLi in C_6D_6 after 10 minutes at room temperature. The major product, (^{tBu}PBP)Pd($CH_2CH_2CH_2CH_3$), is at 94.96 ppm and the decomposition product, ^{tBu}PB^{nBu}P, is at 14.54 ppm.

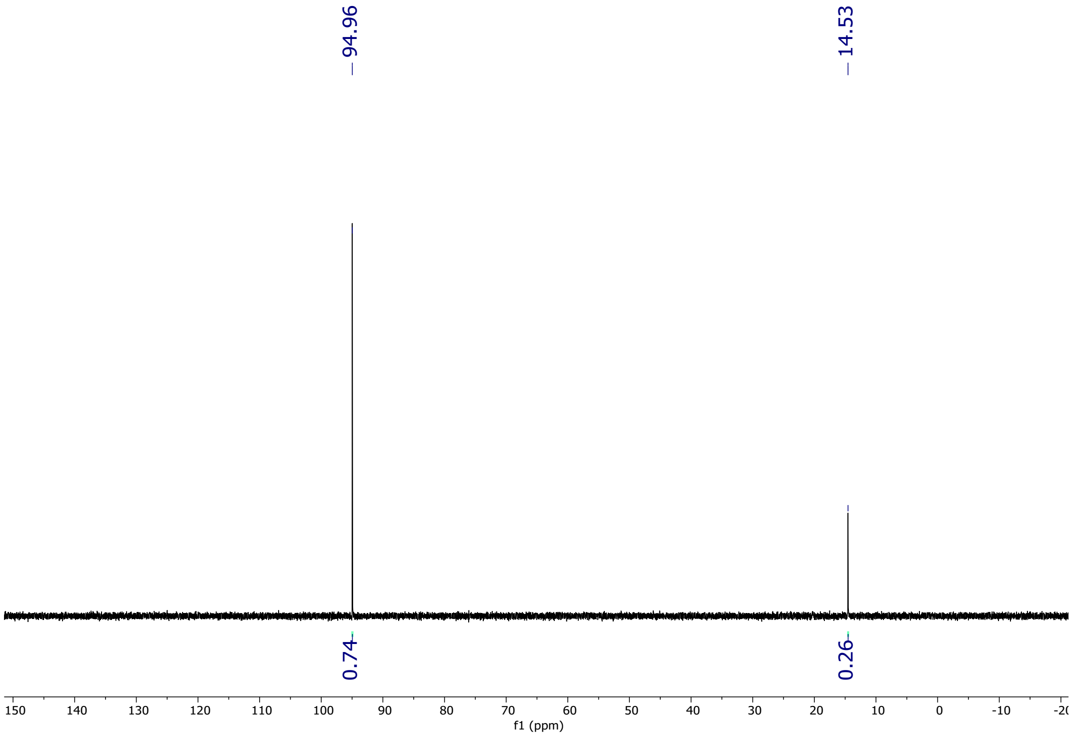

Figure S9. $^{31}P\{^{1}H\}$ NMR of the crude reaction mixture of (^{tBu}PBP)PdCl with ^{n}BuLi in C_6D_6 after 2 hours at room temperature. The decomposition product, ^{tBu}PB^{nBu}P (14.53 ppm), is growing in as (^{tBu}PBP)Pd($CH_2CH_2CH_2CH_3$) (94.96 ppm) depletes.

Reaction of (^{tBu}PBP)PdCl and $LiHBEt_3$ (Superhydride)

(^{tBu}PBP)PdCl (5.0 mg, 0.0087 mmol) was dissolved in 0.5 mL of C_6D_6 and added to a J-Young NMR tube. 5.5 µL of a 1.7 M $LiHBEt_3$ solution in cold THF (0.0092 mmol, 1.05 equivalents) was added dropwise to the C_6D_6 solution. An immediate color change to yellow was observed. Two major resonances were observed in the $^{31}P\{^1H\}$ NMR spectrum recorded 25 minutes after the reaction started. The major product is a relatively downfield shifted peak at 115 ppm, which is assigned as the palladium hydride, (^{tBu}PBP)PdH. This shift is consistent with the $^{31}P\{^1H\}$ NMR shift of the platinum congener, (^{tBu}PBP)PtH, at 114.5 ppm.[3] The major peaks in the ^1H NMR spectrum align with what is expected for the (^{tBu}PBP)PdH, and the hydride peak appears at 2.13 ppm. The second major peak appears at 96.4 ppm and although we are unable to assign this species, it is always present when (^{tBu}PBP)PdH is directly synthesized, or formed through β-hydride elimination. After 12 hours, the (^{tBu}PBP)PdH peak is mostly converted to a new species at 63.5 ppm in the $^{31}P\{^1H\}$ NMR. This complex readily crashes out of C_6D_6 as colorless X-ray diffraction quality crystals. XRD studies reveal that this complex is (^{tBu}PB^{H}P)$_2$Pd$_2$ (**1-H**), a Pd(0) dimer where each Pd is bridging across two ^{tBu}PBP ligands, and the hydrides reside on the boron atoms.

For (^{tBu}PBP)PdH: ^1H NMR (500 MHz, C_6D_6): δ 7.19 (dd, J = 5.6, 3.1 Hz, 2H, CH_{Ar}), 7.05 (dd, J = 5.5, 3.2 Hz, 2H, CH_{Ar}), 3.8 (vt, J = 2.1 Hz, 4H, CH_2), 2.13 (t, J = 19.6 Hz, 1H, Pd-H), 1.26 (t, J = 6.9 Hz, 36H, C(CH_3)$_3$). $^{31}P\{^1H\}$ NMR (202 MHz, C_6D_6): δ 115.0 (s, ^{tBu}*P*B*P*).

For (^{tBu}PB^{H}P)$_2$Pd$_2$: $^{31}P\{^1H\}$ NMR (202 MHz, C_6D_6): δ 63.5 (br s, ^{tBu}*P*B^H*P*).

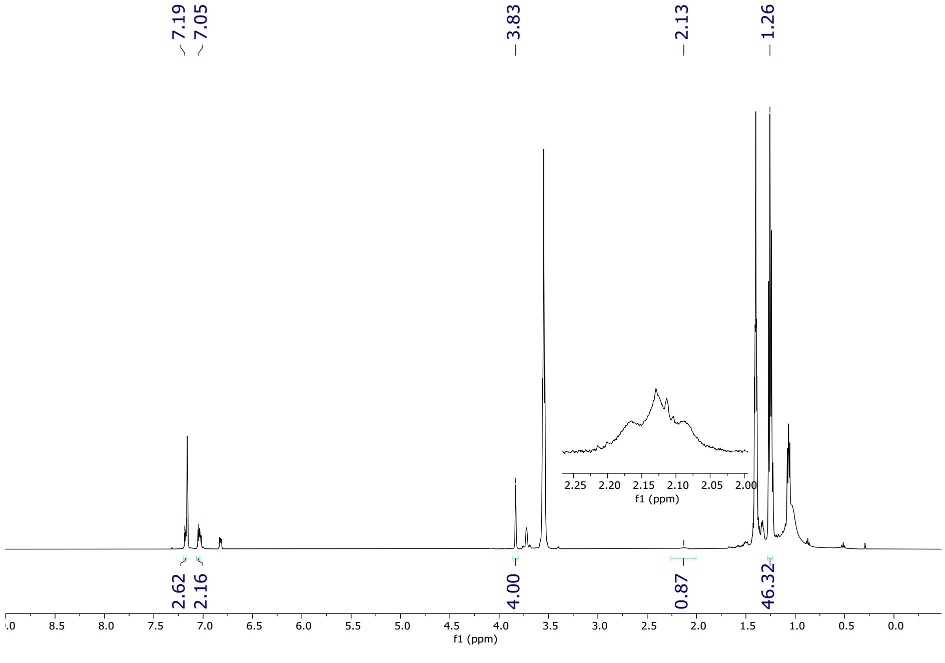

Figure S10. ^1H NMR spectrum of the reaction of (^{tBu}PBP)PdCl and $LiHBEt_3$ at room temperature after 25 minutes. The major peaks, which correspond to (^{tBu}PBP)PdH, are picked and integrated, and the Pd-H peak at 2.13 ppm is enlarged.

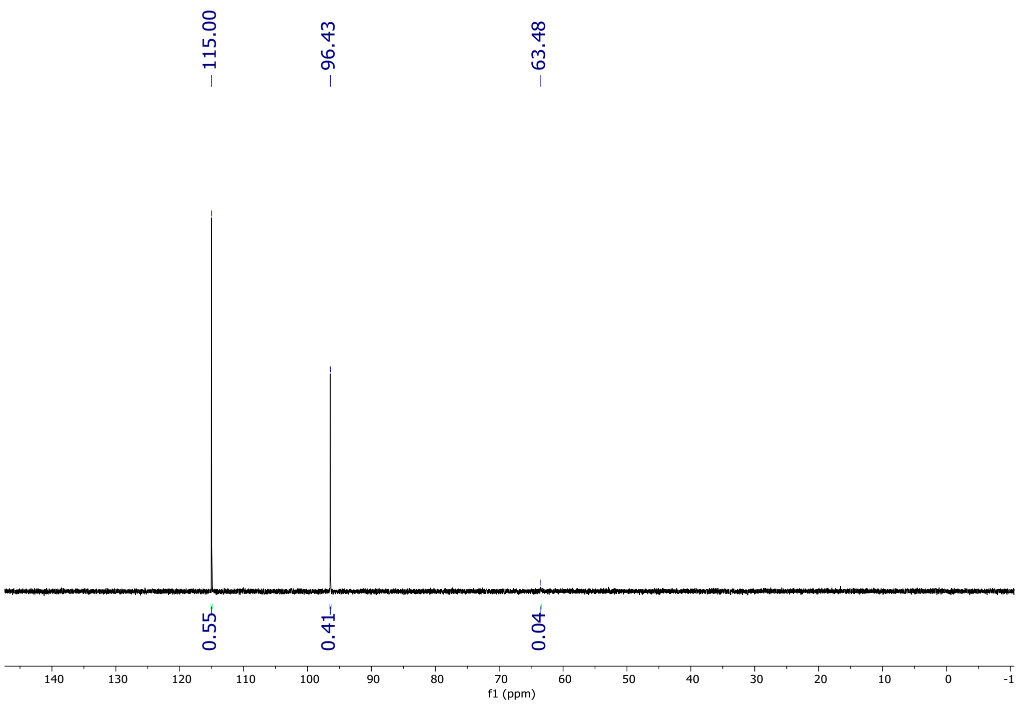

Figure S11. $^{31}P\{^1H\}$ NMR spectrum of the reaction of (^{tBu}PBP)PdCl and $LiHBEt_3$ at room temperature after 25 minutes. The major peak at 115 ppm corresponds to (^{tBu}PBP)PdH and the minor peak at 63.5 ppm corresponds to ($^{tBu}PB^HP)_2Pd_2$ (**2-H**). A third peak is present at 96.4 ppm, which we are unable to assign.

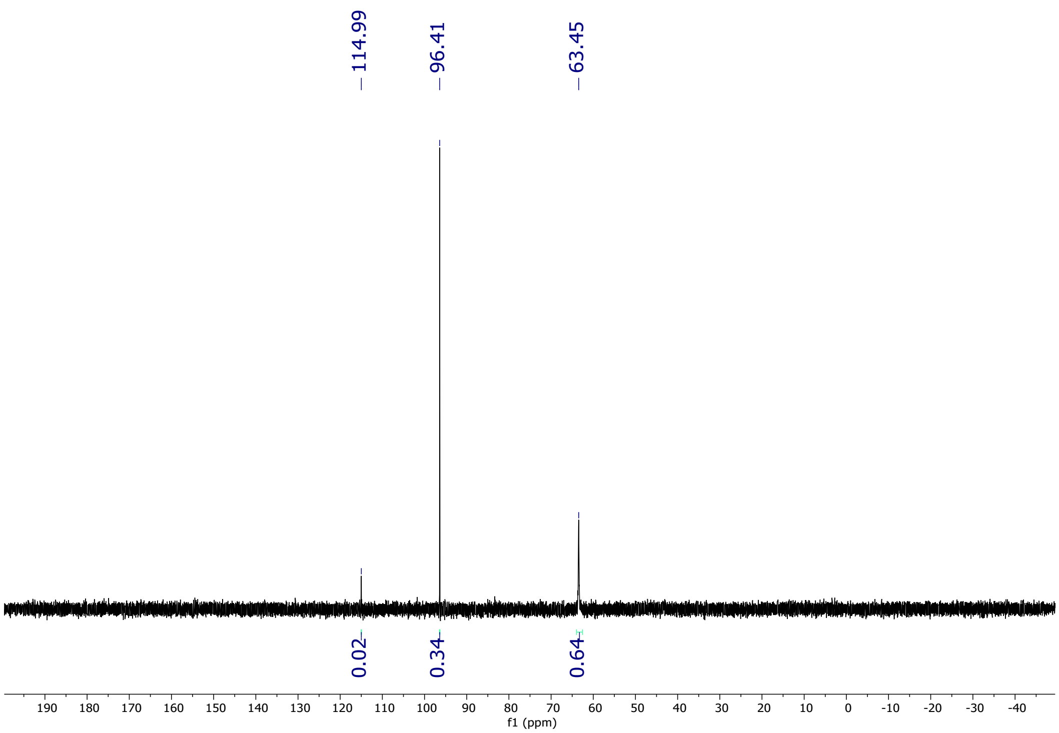

Figure S12. $^{31}P\{^1H\}$ NMR spectrum of the reaction of (^{tBu}PBP)PdCl and $LiHBEt_3$ at room temperature after 12 hours. The major peak at 115 ppm has almost fully converted to ($^{tBu}PB^HP)_2Pd_2$ (**2-H**), evidenced by the peak at 63.5 ppm. A third peak is present at 96.4 ppm, which we are unable to assign.

<u>Reaction of (^{tBu}PBP)PdCl and allylMgCl</u>

(^{tBu}PBP)PdCl (5.2 mg, 0.0090 mmol) was dissolved in 0.5 mL of C_6D_6 and added to a J-Young NMR tube. 0.01 mL of a 1.7 M allylMgCl solution in THF (0.017 mmol, 1.8 equivalents) was added dropwise to the C_6D_6 solution. An immediate color change from colorless to neon yellow was observed. Two major and three minor resonances were present in the $^{31}P\{^1H\}$ NMR spectrum recorded 10 minutes after the start of the reaction. The largest peak at 92.8 ppm (54% by integration) likely corresponds to (^{tBu}PBP)Pd(CH_2CHCH_2) (**1-All**), and the second largest peak at 41.6 ppm (29% by integration) is the decomposition dimer, (^{tBu}PB^{allyl}P)$_2$Pd$_2$ (**2-All**). After 2 hours, full conversion to (^{tBu}PB^{allyl}P)$_2$Pd$_2$ was observed. (^{tBu}PB^{allyl}P)$_2$Pd$_2$ readily crystallizes out of the C_6D_6 solution, allowing for structural characterization using single crystal X-ray diffraction.

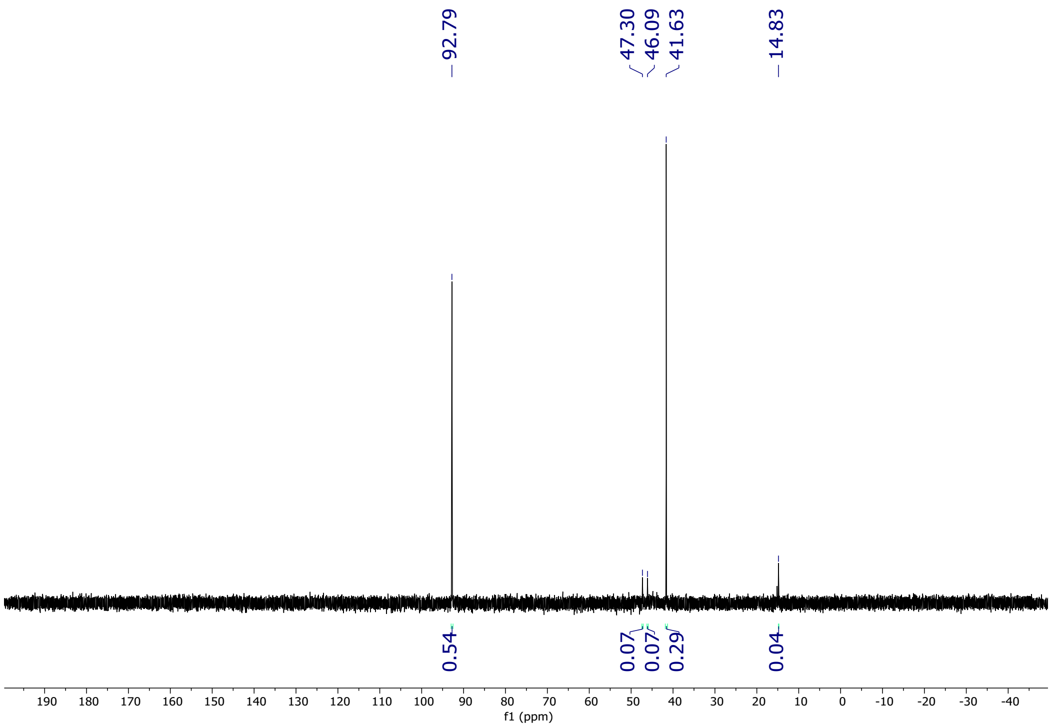

Figure S13. $^{31}P\{^1H\}$ NMR spectrum of the reaction of (^{tBu}PBP)PdCl and allylMgCl at room temperature after 10 minutes. The largest peak at 92.79 ppm (54% by integration) likely corresponds to the desired product, **1-All**, based on its chemical shift. The second major product at 41.63 ppm (29%) is the decomposition dimer **2-All**. The minor peaks are unknown impurities.

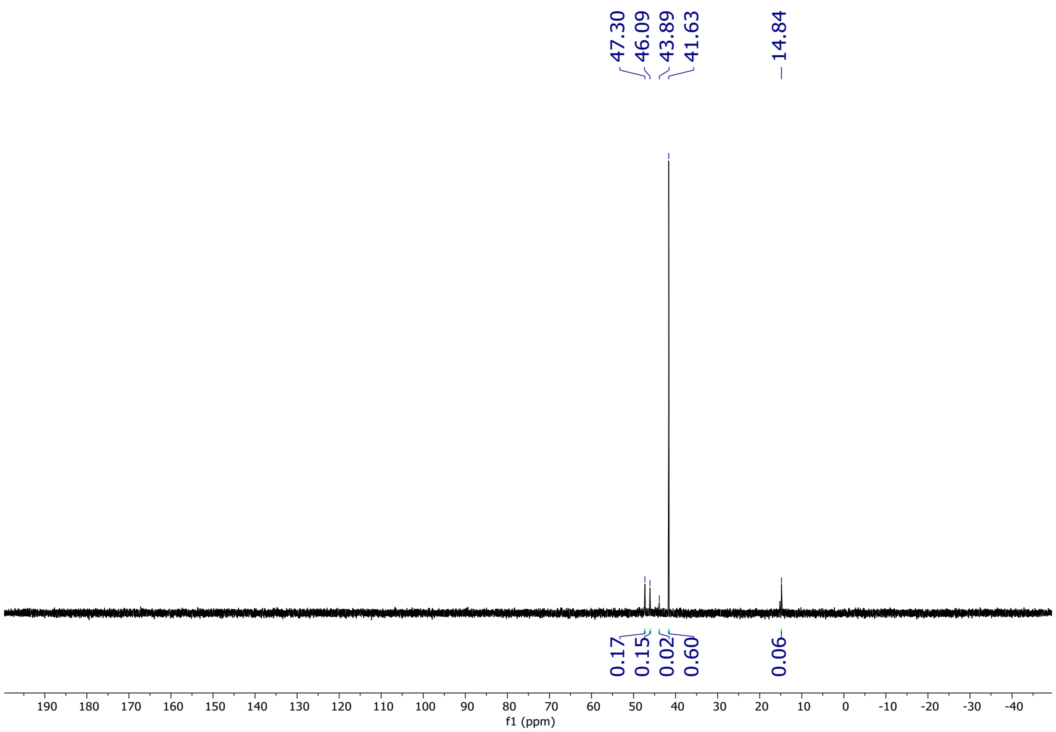

Figure S14. $^{31}P\{^{1}H\}$ NMR spectrum of the reaction of (^{tBu}PBP)PdCl and allylMgCl at room temperature after 2 hours. The resonance at 92.79 ppm (**1-All**) has fully disappeared and the resonance at 41.63 ppm (**2-All**, 60%) has grown. The minor peaks are unknown impurities.

SIV: Determination of Qualitative Rates of CO_2 Insertion

Qualitative rates of CO_2 insertion were obtained through room temperature NMR studies in C_6D_6. For (^{tBu}PBP)Pd(CH_2CH_3), the reaction was complete in 2 hours, which also allowed for quantitative kinetic studies. Similarly, the previously reported reaction of CO_2 and (^{tBu}PBP)Pd(CH_3) under these conditions is done in 6 hours.[2] However, CO_2 insertion into (^{tBu}PBP)Pd($CH_2CH_2CH_3$) is much slower. Here, reaction progress was monitored periodically via ^{1}H and ^{31}P{^{1}H} NMR spectroscopy to determine reaction half-lives and the completion of the reaction, as continuous NMR spectroscopy throughout the reaction was not feasible. ^{31}P{^{1}H} NMR spectra of the first four half-lives are shown in Figure S15. The half-life of the reaction was found to be 11 hours, and full conversion was observed after 3 days. An external triphenylphosphine oxide standard was used to ensure mass balance throughout the reaction.

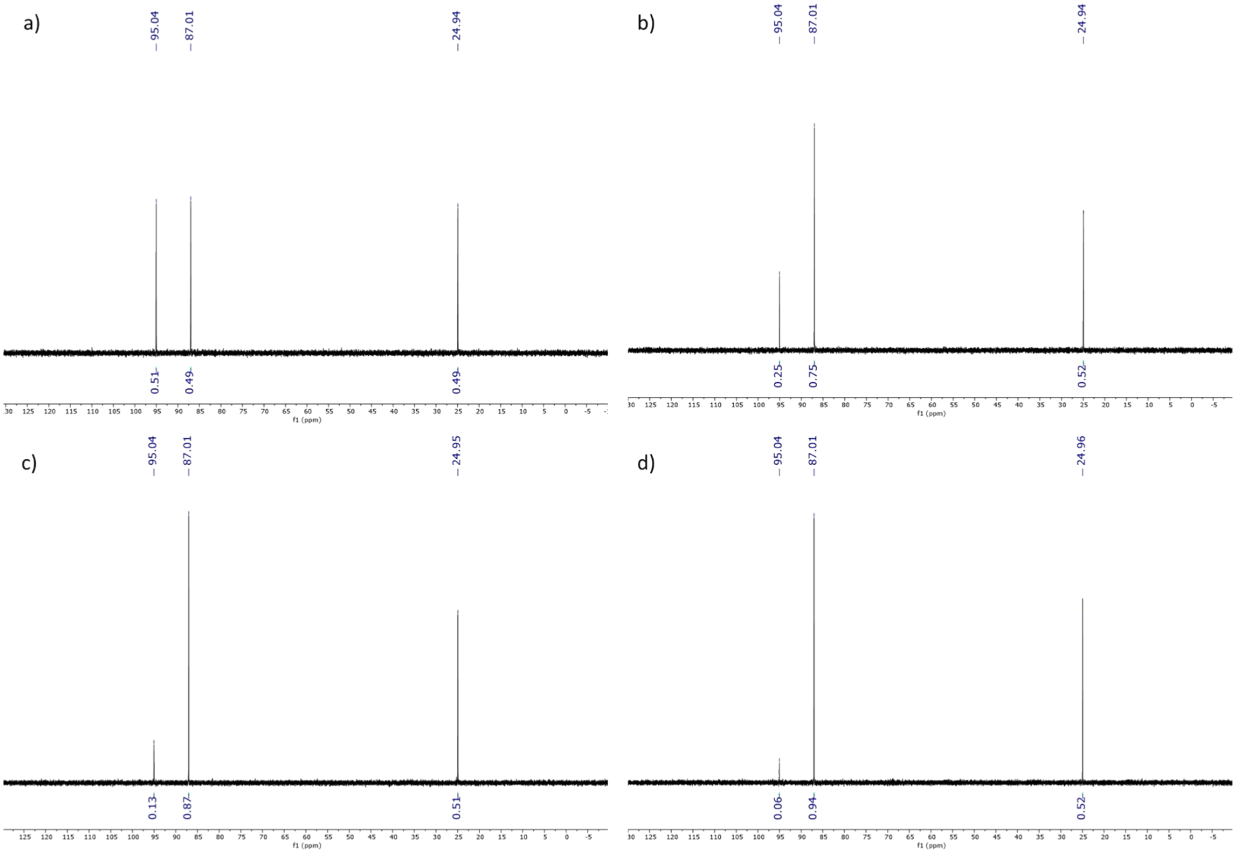

Figure S15. ^{31}P{^{1}H} NMR spectrum of the reaction of 1 atm CO_2 with (^{tBu}PBP)Pd($CH_2CH_2CH_3$) in C_6D_6 at room temperature after **a)** 11 hours, at approximately 1 half-life, **b)** 22 hours, at approximately 2 half-lives, **c)** 33 hours, at approximately 3 half-lives, and **d)** 44 hours at approximately 4 half-lives. The (^{tBu}PBP)Pd($CH_2CH_2CH_3$) peak at 95.04 ppm slowly disappears at the same rate as the (^{tBu}PBP)Pd{OC(O)$CH_2CH_2CH_3$} peak at 87.01 ppm grows in. An external standard ($OPPh_3$, 24.96 ppm) was used to ensure mass balance.

SV. Representative Kinetic Data Workup: Determination of k_1 Using ^{1}H NMR Spectroscopy

Experimental

In a nitrogen-filled glovebox, (^{tBu}PBP)Pd(CH_2CH_3) (2.3 mg, 0.0086 mmol) was added to a J-Young NMR tube with 400 µL of C_6D_6 and 100 µL of a 0.034 M trimethoxybenzene stock solution in C_6D_6. The J-Young NMR tube was attached to a Schlenk line with a J-Young to 24/40 adaptor, and vacuum was pulled on the closed system for at least 30 minutes to remove air. The tube was degassed via three freeze-pump-thaw cycles. 1 atm of CO_2 was introduced into the J-Young NMR tube. The tube was inserted into a 500 MHz NMR spectrometer, with the temperature preset to 30 °C. After the temperature regulated back to 30 °C, the sample was removed and shaken, and placed back into the NMR probe. A ^{1}H NMR array experiment was conducted, where a new ^{1}H spectrum (8 scans) was acquired every 32 seconds (Figure S16).

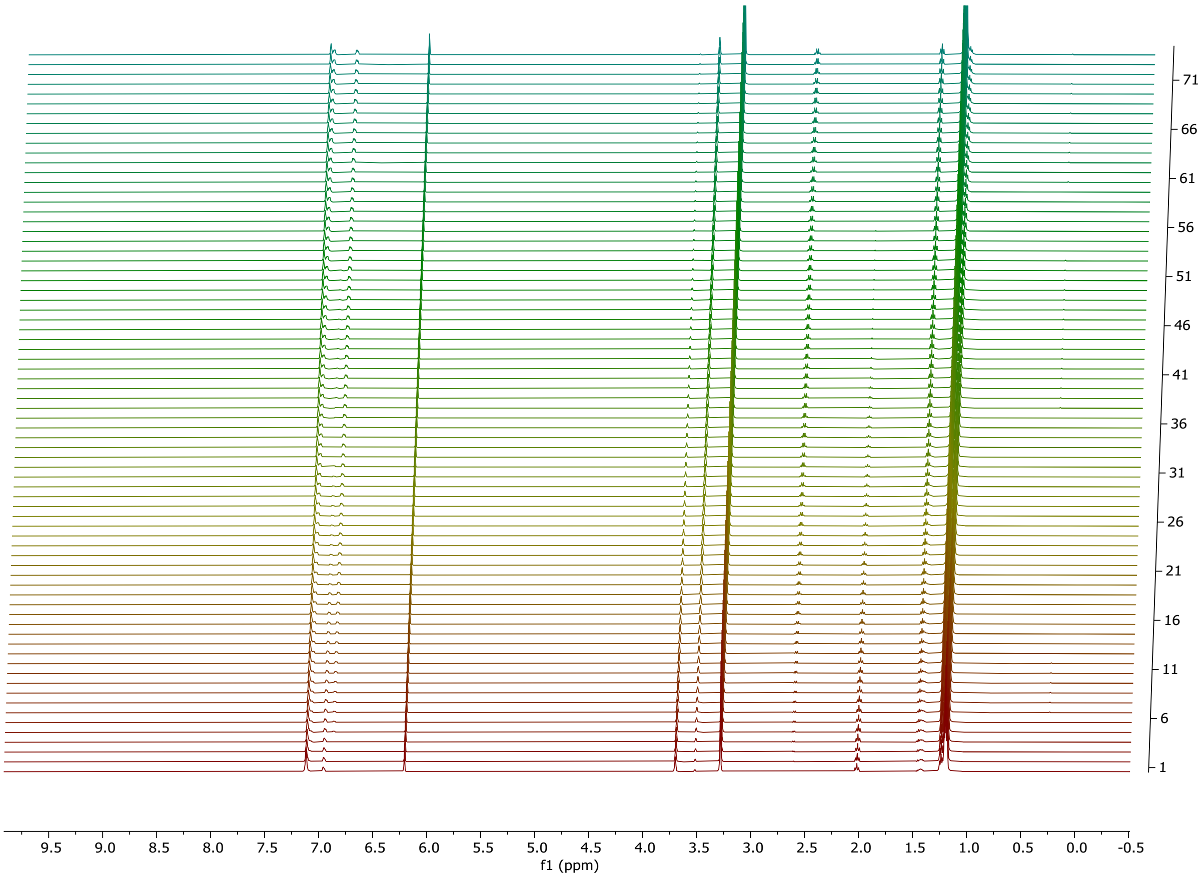

Figure S16. Representative ^{1}H NMR array for the insertion of CO_2 into (^{tBu}PBP)Pd(CH_2CH_3) at 30 °C in C_6D_6.

Data Workup

The ^{1}H NMR array was baseline and phase corrected before integration of the (^{tBu}PBP)Pd(CH_2CH_3) (3.73 and 2.09 ppm), (^{tBu}PBP)Pd{OC(O)CH_2CH_3} (3.55 and 2.66 ppm), and trimethoxybenzene standard (6.25 and 3.3 ppm) peaks. The disappearance of the -CH_2 and -CH_3 peaks of the (^{tBu}PBP)Pd(CH_2CH_3) complex

and the appearance of the -CH_2 and -CH_3 peaks of the (^{tBu}PBP)Pd{OC(O)CH_2CH_3} complex could be used to monitor reaction progress. Ultimately, the disappearance of the (^{tBu}PBP)Pd(CH_2CH_3) -CH_3 peak at 2.09 ppm was used to determine kinetic rates. Using the Data Analysis feature of Mestrenova, a table of the absolute integrals for each spectrum was generated. The following data analysis was performed using Microsoft Excel. The concentration of (^{tBu}PBP)Pd(CH_2CH_3) was obtained by dividing the absolute integration of the -CH_3 peak by the absolute integration of the trimethoxybenzene standard for each spectrum, then multiplying by 18/3 to correct for number of protons, as well as multiplying by the known concentration of trimethoxybenzene. Once the concentration of (^{tBu}PBP)Pd(CH_2CH_3) was calculated for each spectrum, a graph of ln([(^{tBu}PBP)Pd(CH_2CH_3)]) vs. time was plotted, where the slope of the line yields k_{obs}. The first data point was omitted in all cases due to a broad spectrum as a result of the sample heating up to the NMR probe temperature. A value of k_1 can be extracted from k_{obs} by dividing by the [CO_2], which was determined by quantitative $^{13}C\{^1H\}$ NMR experiments (see SVI). A similar procedure was used to determine the rate of CO_2 insertion into (^{tBu}PBP)Pd(CH_2CH_3) at various temperatures and pressures of CO_2, and for CO_2 insertion into (^{tBu}PBP)Pd($CH_2C_6H_5$) and (^{tBu}PBP)Pd(CH_2-4-OMe-C_6H_4) at 30 °C in pyridine-*d*$_5$.

SVI. Control Experiments to Determine the Effect of Impurities on the Rate of CO_2 Insertion

We performed three control experiments in order to determine if any residual Li or Mg salts from synthesizing the Pd alkyl complexes had an effect on the rate of CO_2 insertion into the Pd alkyl bond. First, we synthesized **1-Et** using either EtMgCl or EtLi and an otherwise identical synthetic route. CO_2 was inserted into **1-Et** from both batches and the rate of insertion of CO_2 into the Pd ethyl bond was measured. The rates of insertion were within error of each other, showing that even if there are salt impurities, there is no difference between the presence of a Mg salt or a Li salt.

Second, we performed kinetic studies on the insertion of CO_2 into **1-^{n}Pr**, which has been recrystallized one, two, or three times. The rate of CO_2 insertion did not change depending on the number of recrystallizations, suggesting that any impurities that may be present after one recrystallization do not significantly affect the rate of insertion. However, the CO_2 insertion was not as clean with only one recrystallization, and some small peaks (<1%) are observed in the NMR spectra.

Third, we intentionally added LiCl (2.5 mg) to a J-Young NMR tube containing **1-^{n}Pr** (2.3mg) in 0.5 mL C_6D_6 and inserted CO_2. Since LiCl is not very soluble in C_6D_6, there was solid LiCl present at the bottom of the tube throughout the reaction. We did not observe a significant change in the rate of the reaction, but again the reaction was not as clean as we had previously observed and some small peaks (<2%) are observed in the NMR spectra. These three experiments suggest that if there are any residual salts in our purified complexes, they do not play a role in the large differences in rates between the different alkyl complexes and that the identity of the salt (Mg vs Li) does not explain these large differences either.

SVII. Determination of $[CO_2]$ by Quantitative $^{13}C\{^1H\}$ NMR Spectroscopy

In order to obtain k_1 values from experimentally measured k_{obs} values, the concentration of CO_2 dissolved in solution must be known. A modified literature procedure was used to measure $[CO_2]$ using quantitative $^{13}C\{^1H\}$ NMR spectroscopy, which our group has reported in the past.[2,4] Specifically, a J-Young NMR tube was charged with 500 μL of solvent and 10-20 μL of toluene as an internal standard. The solution was degassed via three freeze-pump-thaw cycles and 1 atm CO_2 was added via a Schlenk line. The J-Young NMR tube was then inserted into a 500 MHz NMR spectrometer set to the desired temperature, and an inverse-gated $^{13}C\{^1H\}$ NMR spectrum was acquired using a relaxation delay of 60 seconds. The concentration of CO_2 was determined through comparison of the integration of the toluene quaternary carbon peak at 137.8 ppm with the integration of the CO_2 peak at 124.9 ppm. A representative $^{13}C\{^1H\}$ NMR of $[CO_2]$ in pyridine-d_5 at 30 °C is shown in Figure S17. Each experiment was run in duplicate and the results are reported in Table S1. Measured values of the concentration of CO_2 in C_6D_6 at several temperatures have been previously reported by our group and those values were used where possible.[2]

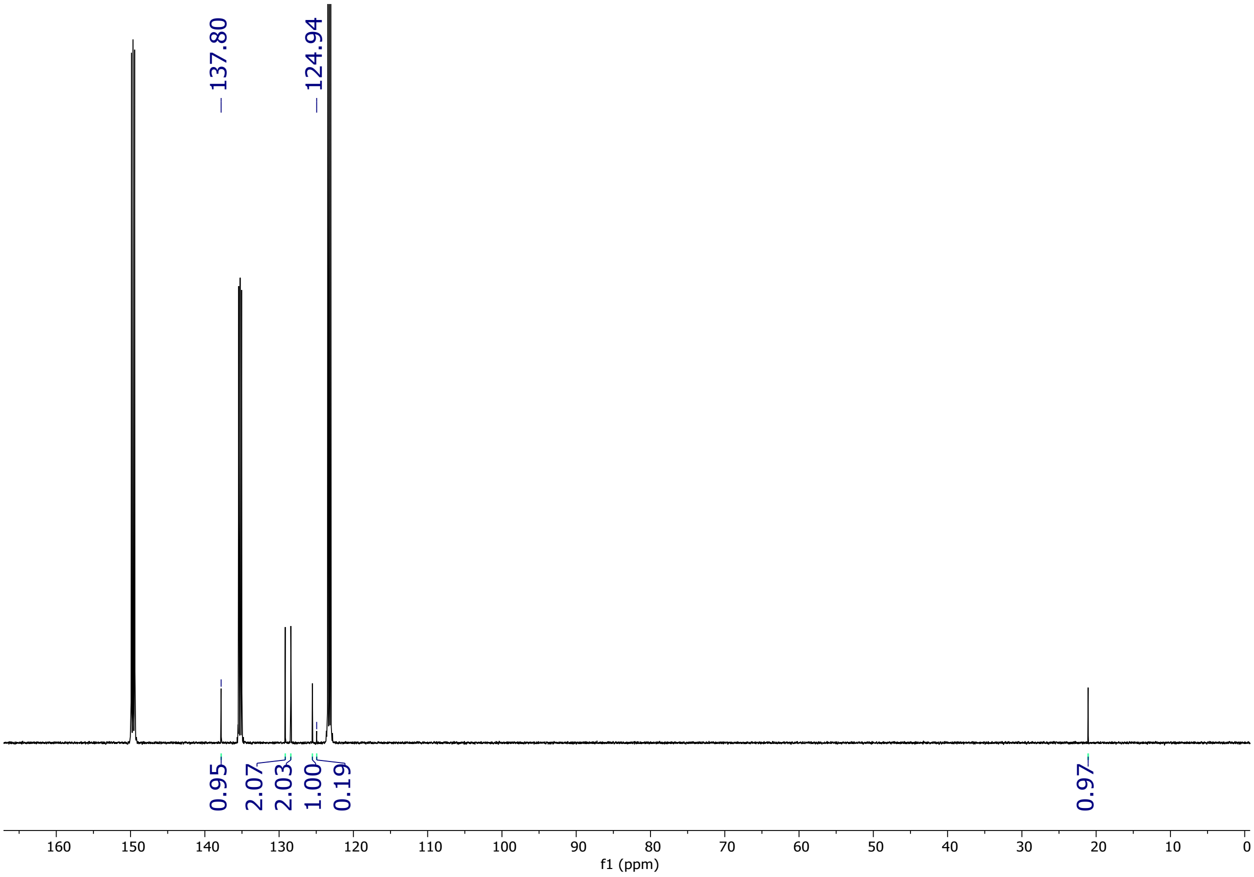

Figure S17. Determination of $[CO_2]$ in pyridine-d_5 at 30 °C using $^{13}C\{^1H\}$ NMR spectroscopy. The integration of the quaternary toluene standard peak (137.8 ppm) with known concentration is compared to the integration of the CO_2 peak (124.9 ppm) to determine $[CO_2]$.

Solvent	Temperature (°C)	$[CO_2]$ (M)
Benzene-*d*$_6$	25	0.069
Benzene-*d*$_6$	30	0.062
Benzene-*d*$_6$	35	0.056
Pyridine-*d*$_5$	30	0.083

Table S1. Summary of the measured $[CO_2]$ in different solvents and at different temperatures. The concentration of CO_2 is the average of two trials.

SVIII. Reaction Order in CO_2 for the Insertion of CO_2 into (^{tBu}PBP)Pd(CH_2CH_3)

A first order rate dependence on CO_2 for the insertion of CO_2 into (^{tBu}PBP)Pd(CH_2CH_3) was confirmed by varying the pressure of CO_2 added to the J-Young tube from 0.63 – 1.0 atm.

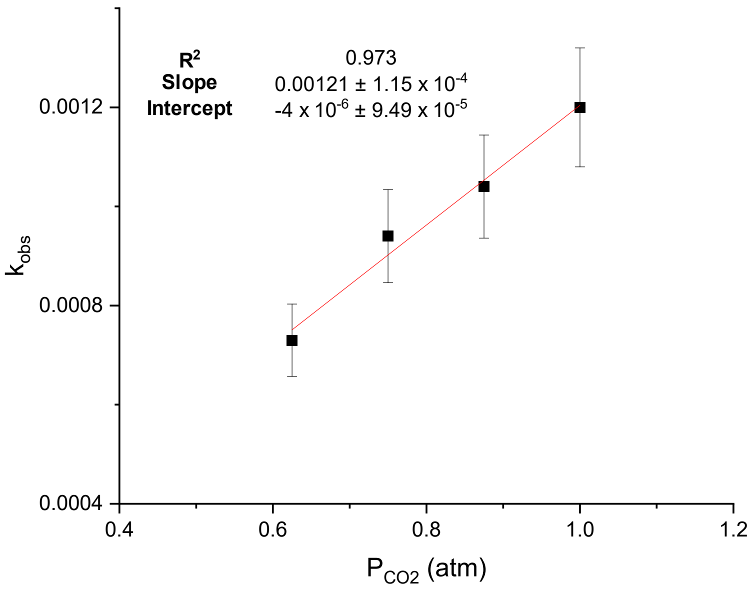

Figure S18. Reaction order in [CO_2] of the insertion of CO_2 into (^{tBu}PBP)Pd(CH_2CH_3) in C_6D_6 at 30 °C.

SIX. Eyring Analysis for the Insertion of CO_2 into (^{tBu}PBP)Pd(CH_2CH_3) in C_6D_6

The rate of insertion of CO_2 into (^{tBu}PBP)Pd(CH_2CH_3) was obtained at temperatures varying from 25-45 °C (Figure S19). Eyring plots were constructed from this data in order to obtain values for $\Delta S^‡$ and $\Delta H^‡$. $\Delta G^‡$ was calculated using $\Delta G^‡ = \Delta H^‡ - T\Delta S^‡$.

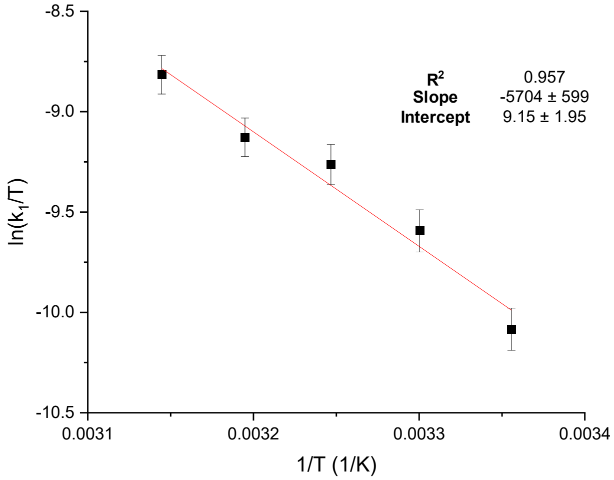

Figure S19. Eyring plot for the reaction of (^{tBu}PBP)Pd(CH_2CH_3) with 1 atm CO_2 in C_6D_6. Activation parameters: $\Delta H^‡ = 11.3 \pm 1.1$ kcal mol^{-1}, $\Delta S^‡ = -29.0 \pm 2.9$ cal mol^{-1} K^{-1}, and $\Delta G^‡ = 20.0 \pm 2.0$ kcal mol^{-1}.

SX. NMR and IR Spectra of New Compounds

(^{tBu}PBP)Pd(CH_2CH_3) (**1-Et**)

NMR spectra for (^{tBu}PBP)Pd(CH_2CH_3) at 25 °C in C_6D_6 are shown in Figures S20-S22.

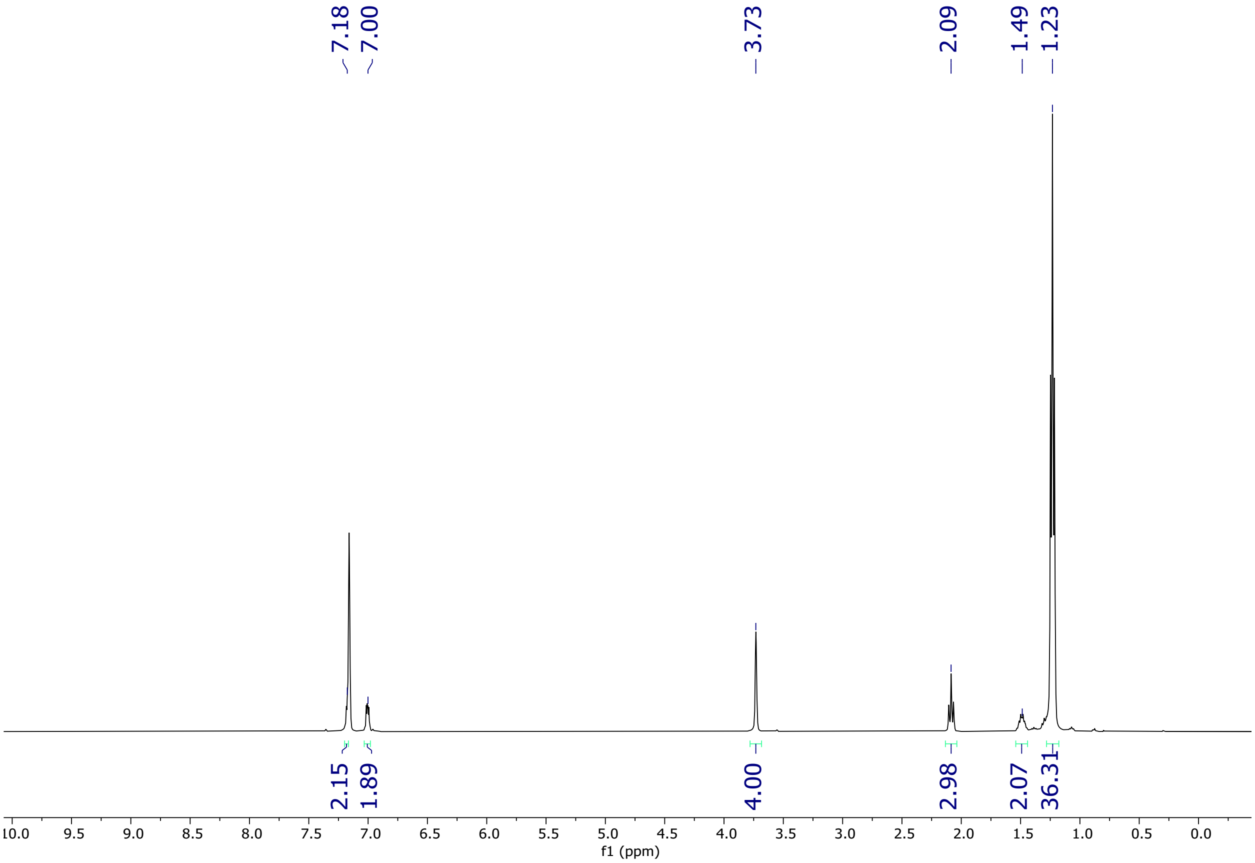

Figure S20. ^{1}H NMR spectrum of (^{tBu}PBP)Pd(CH_2CH_3) in C_6D_6 at room temperature.

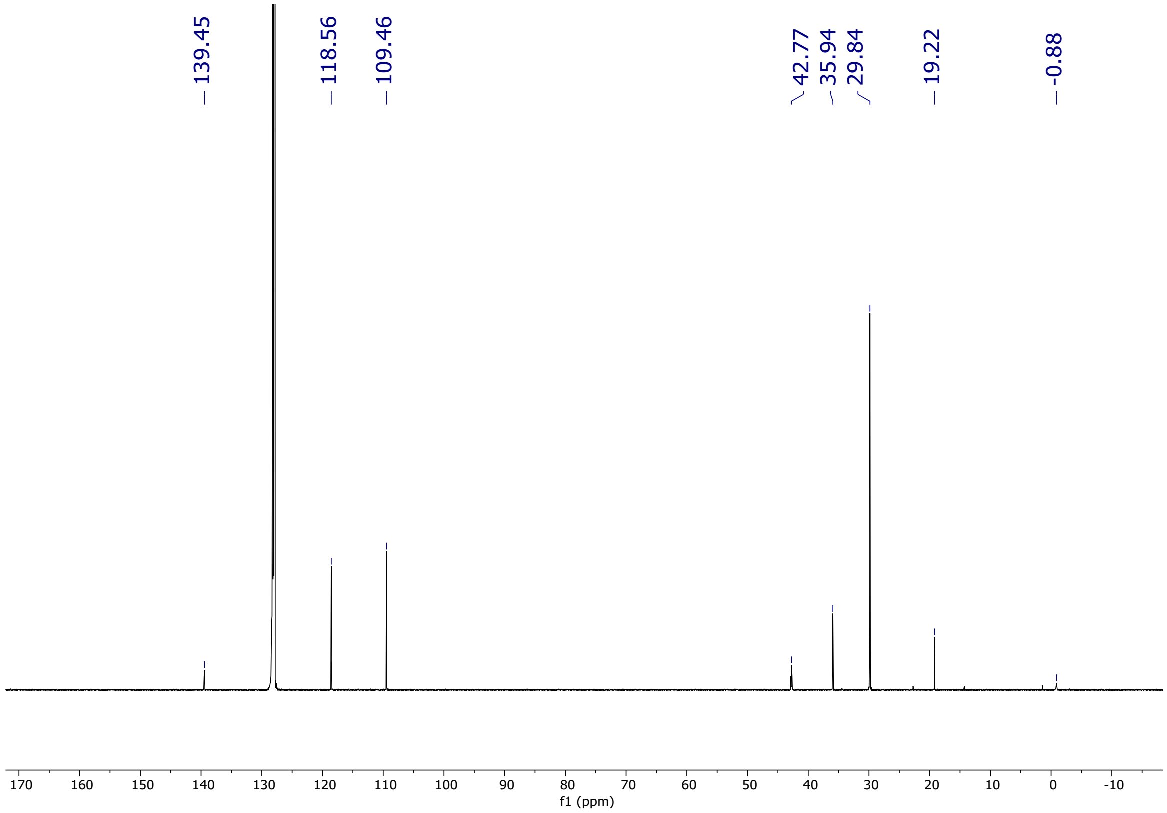

Figure S21. $^{13}C\{^1H\}$ NMR spectrum of (^{tBu}PBP)Pd(CH_2CH_3) in C_6D_6 at room temperature.

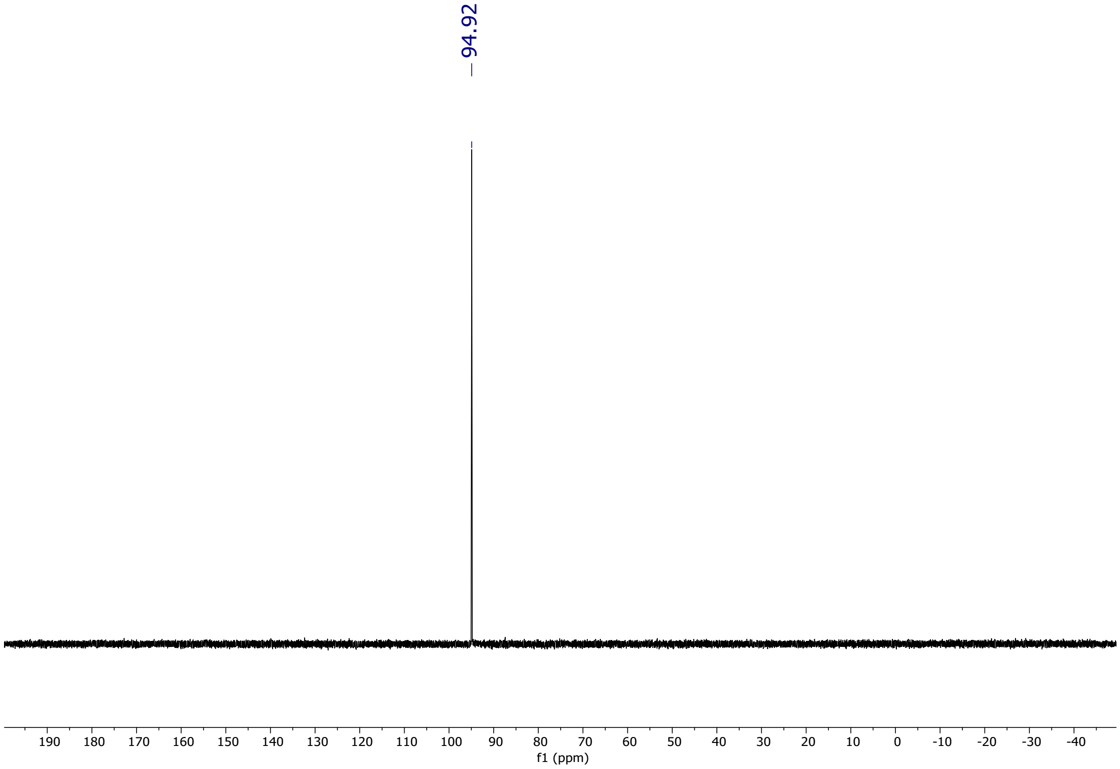

Figure S22. $^{31}P\{^1H\}$ NMR spectrum of (^{tBu}PBP)Pd(CH_2CH_3) in C_6D_6 at room temperature.

(^{tBu}PBP)Pd($CH_2CH_2CH_3$) (**1-^{n}Pr**)

NMR spectra for (^{tBu}PBP)Pd($CH_2CH_2CH_3$) at 25 °C in C_6D_6 are shown in Figures S23-25.

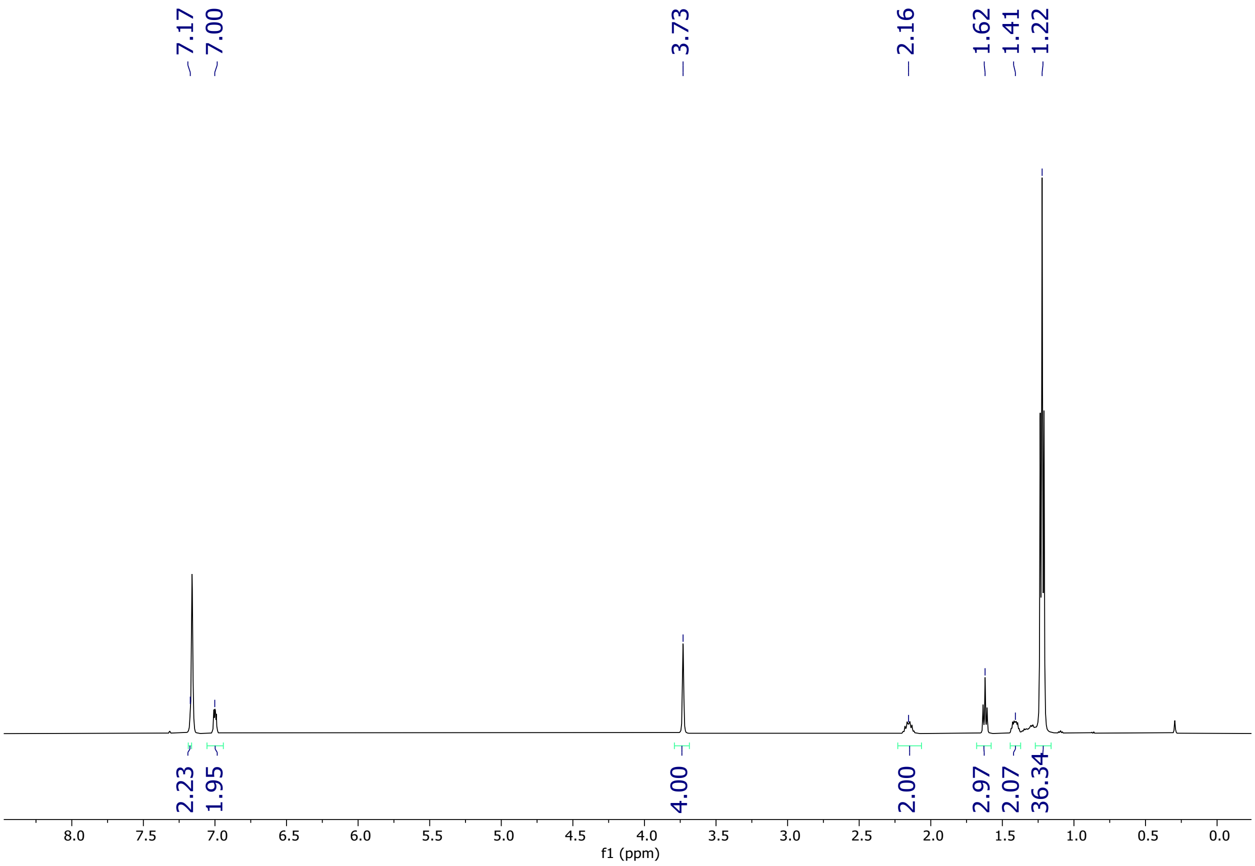

Figure S23. ^{1}H NMR spectrum of (^{tBu}PBP)Pd($CH_2CH_2CH_3$) in C_6D_6 at room temperature.

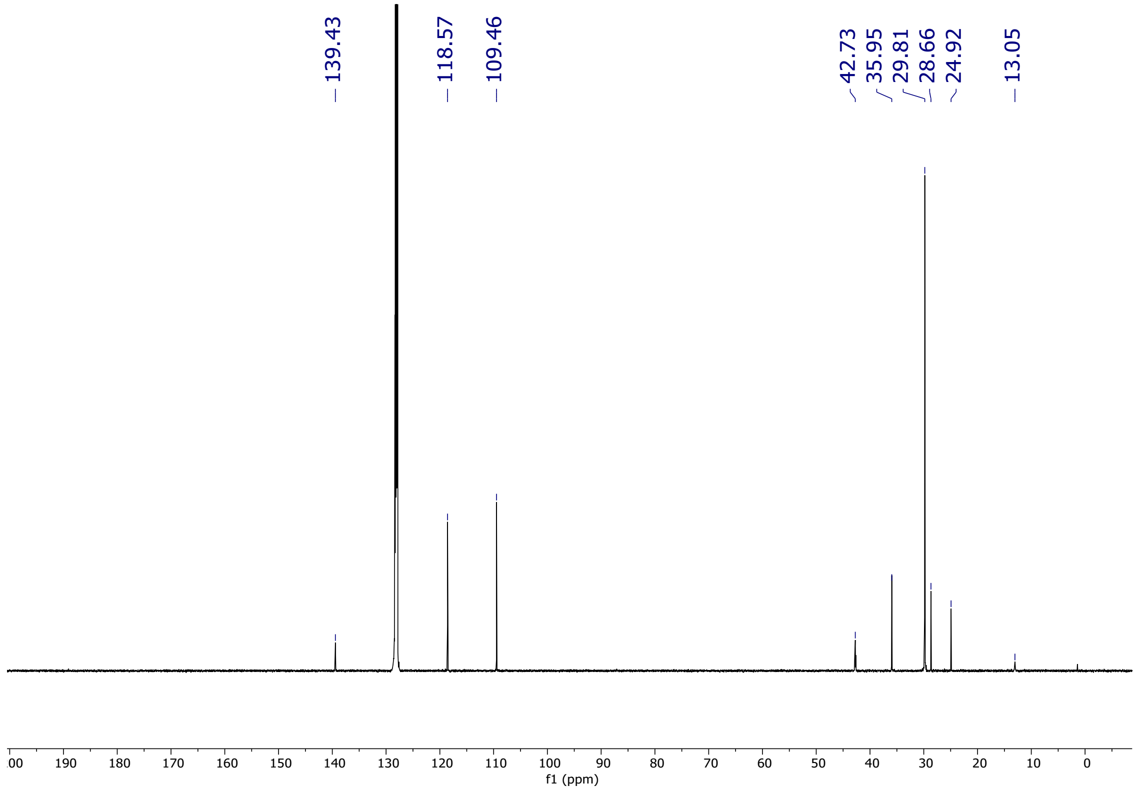

Figure S24. $^{13}C\{^{1}H\}$ NMR spectrum of $(^{tBu}PBP)Pd(CH_2CH_2CH_3)$ in C_6D_6 at room temperature.

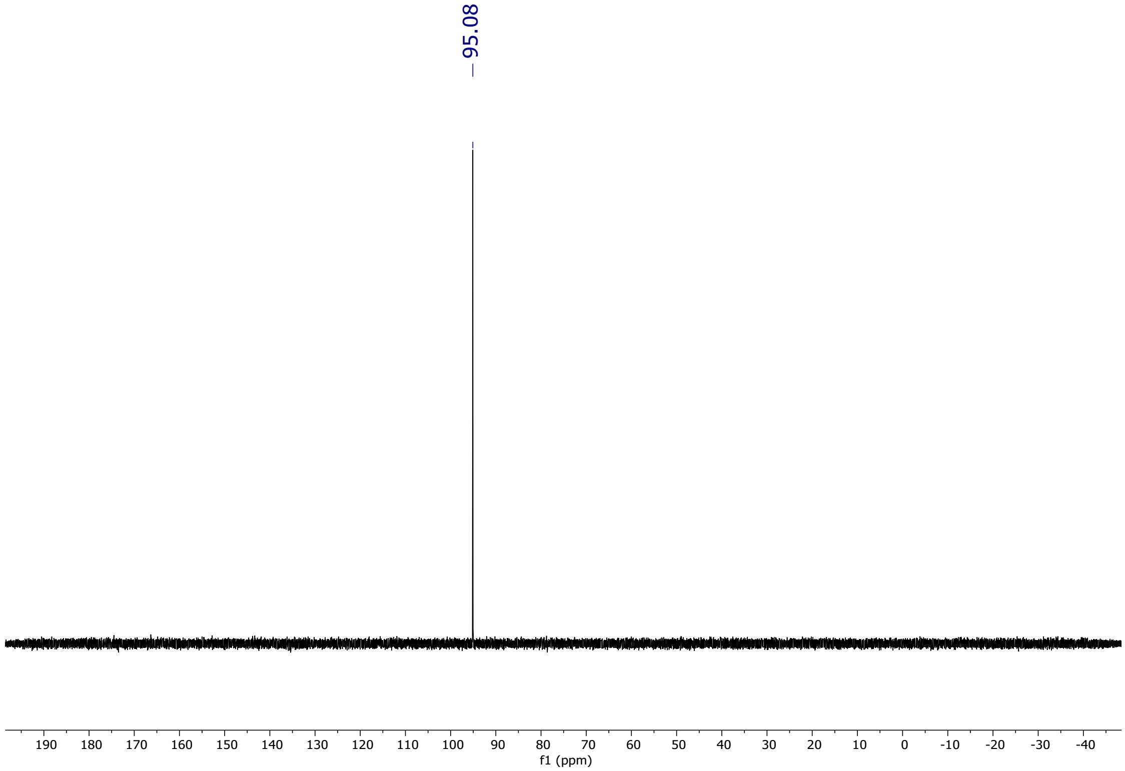

Figure S25. $^{31}P\{^{1}H\}$ NMR spectrum of $(^{tBu}PBP)Pd(CH_2CH_2CH_3)$ in C_6D_6 at room temperature.

(^{tBu}PBP)Pd($CH_2C_6H_5$) (**1-Bn**)

NMR spectra for (^{tBu}PBP)Pd($CH_2C_6H_5$) at 25 °C in C_6D_6 are shown in Figures S26-S28.

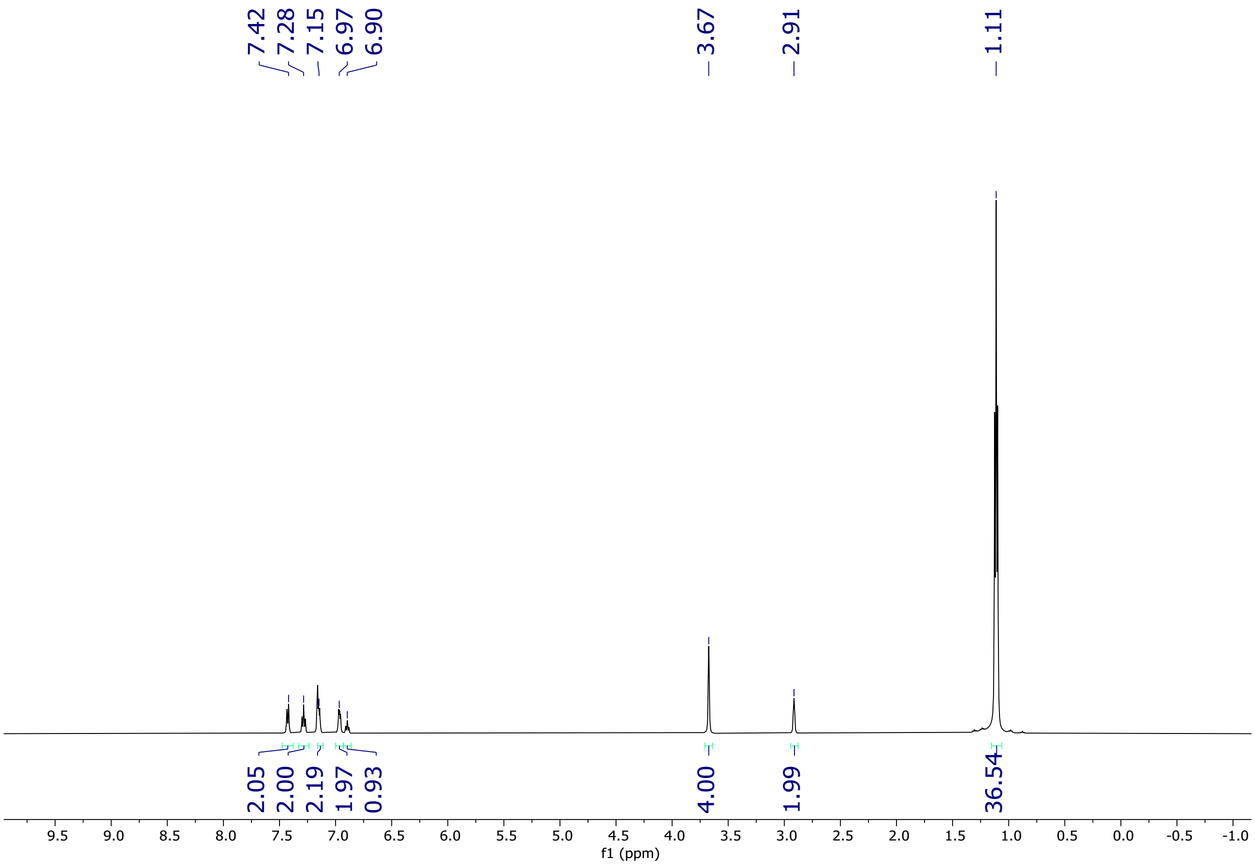

Figure S26. ^1H NMR spectrum of (^{tBu}PBP)Pd($CH_2C_6H_5$) in C_6D_6 at room temperature.

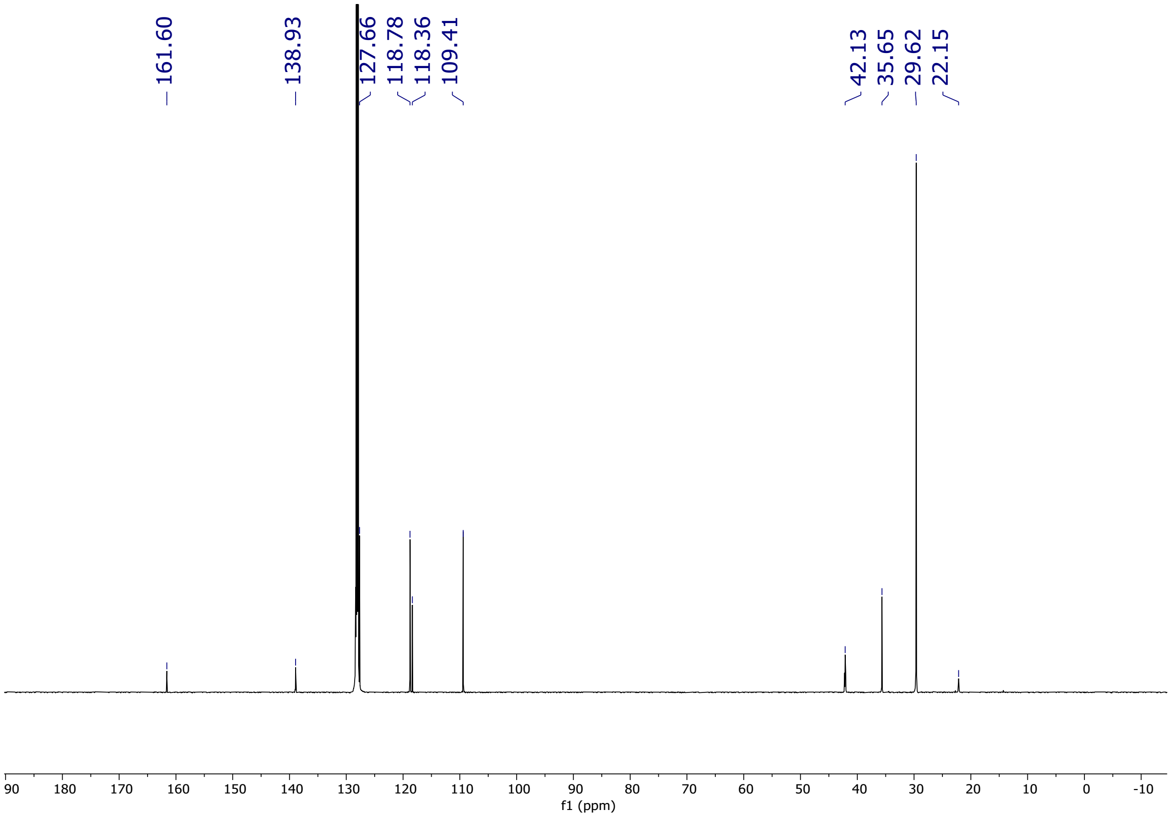

Figure S27. $^{13}C\{^{1}H\}$ NMR spectrum of (^{tBu}PBP)Pd($CH_2C_6H_5$) in C_6D_6 at room temperature.

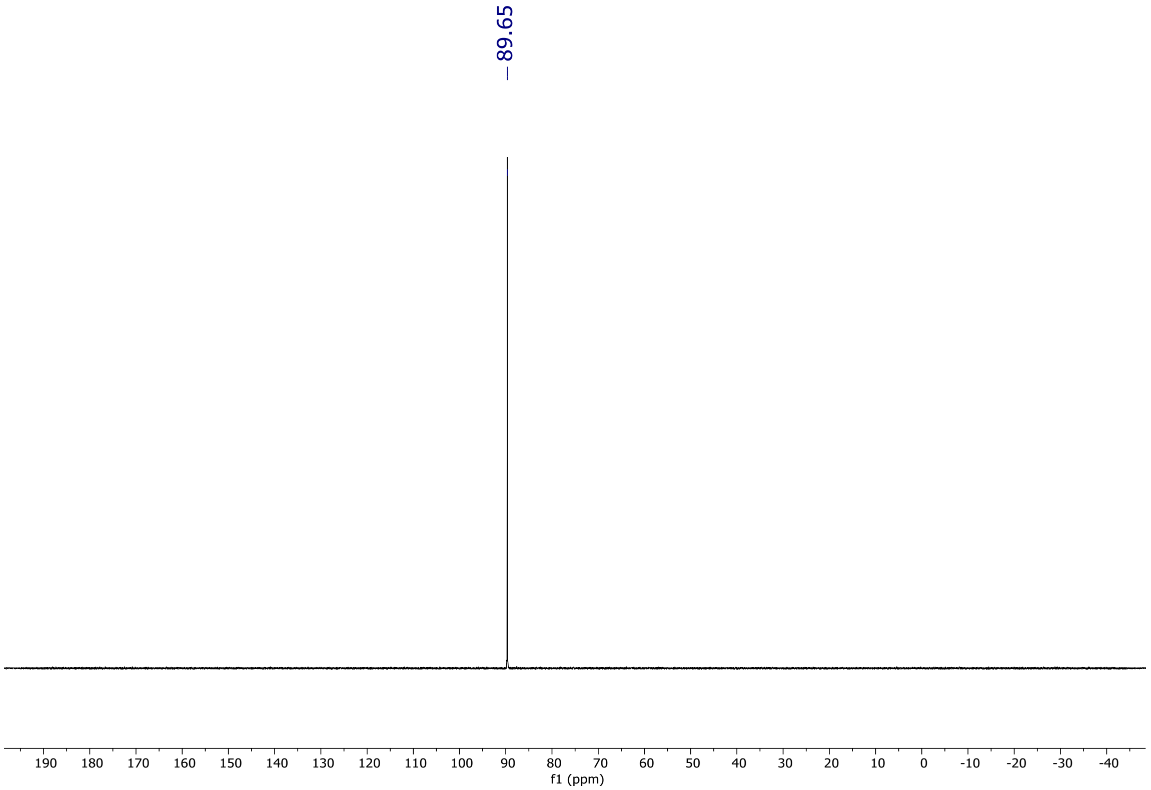

Figure S28. $^{31}P\{^{1}H\}$ NMR spectrum of (^{tBu}PBP)Pd($CH_2C_6H_5$) in C_6D_6 at room temperature.

(^{tBu}PB^{Bn}P)$_2$Pd$_2$ (**2-Bn**)

NMR spectra of (^{tBu}PB^{Bn}P)$_2$Pd$_2$ at 25 °C in C_6D_6 are shown in Figures S29-S30.

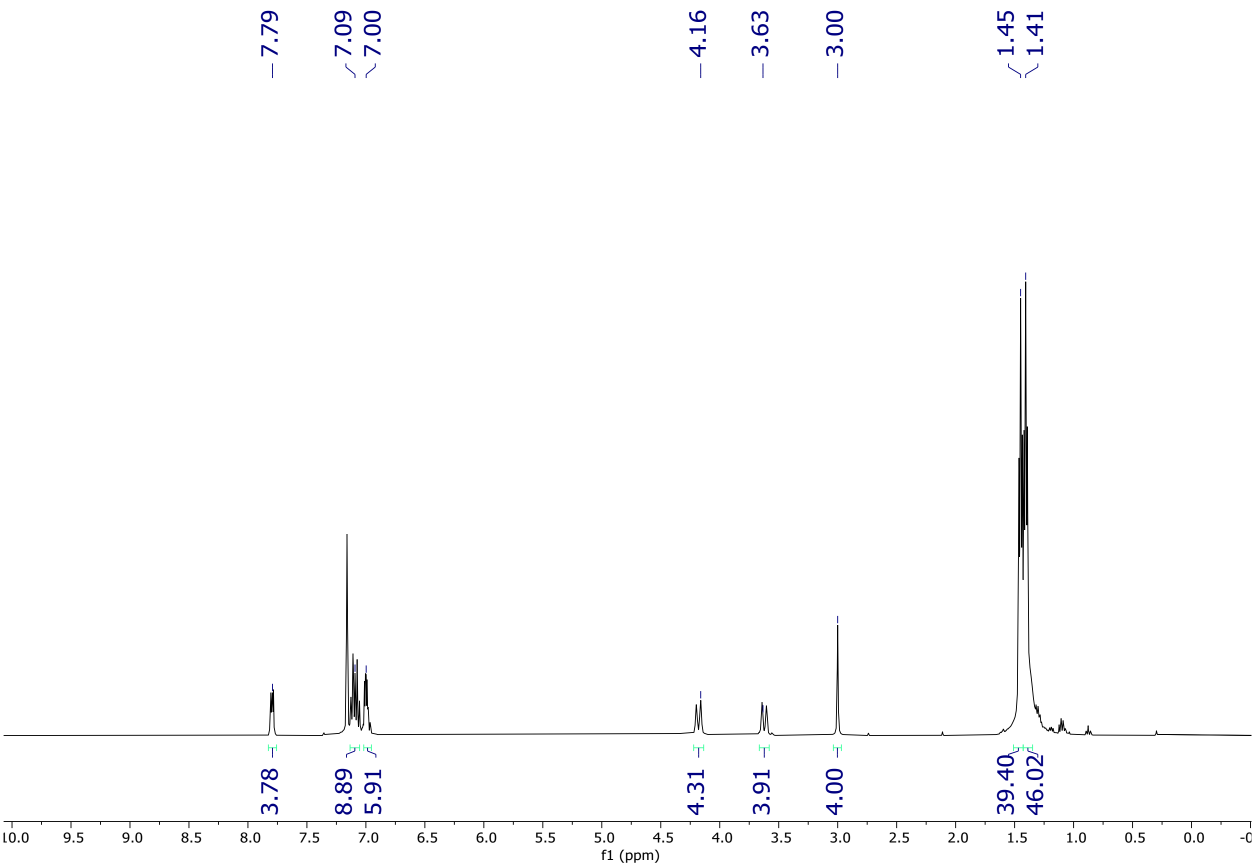

Figure S29. ^{1}H NMR spectrum of the thermal decomposition of (^{tBu}PBP)Pd($CH_2C_6H_5$) after 3 days in C_6D_6 at 65 °C. The major peaks, which correspond to the Pd(0) dimer product, (^{tBu}PB^{Bn}P)$_2$Pd$_2$, are picked and integrated.

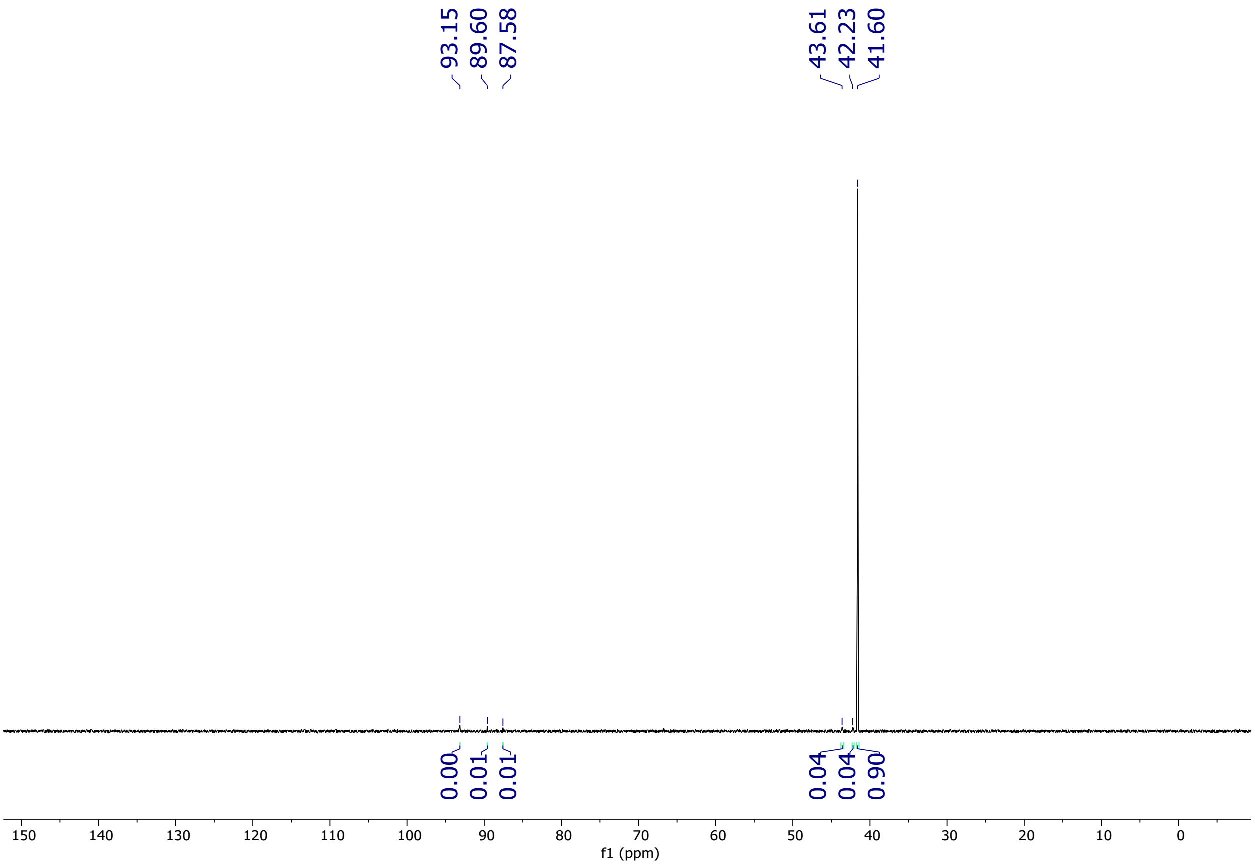

Figure S30. $^{31}P\{^{1}H\}$ NMR spectrum of the thermal decomposition of (^{tBu}PBP)Pd($CH_2C_6H_5$) after 3 days in C_6D_6 at 65 °C. The major peak is at 41.6 ppm, which corresponds to the Pd(0) dimer product, (^{tBu}PB^{Bn}P)$_2$Pd$_2$.

(^{tBu}PBP)Pd(CH_2-4-OMe-C_6H_4) (**1-^{OMe}Bn**)

NMR spectra for (^{tBu}PBP)Pd(CH_2-4-OMe-C_6H_4) at 25 °C in C_6D_6 are shown in Figures S31-S33.

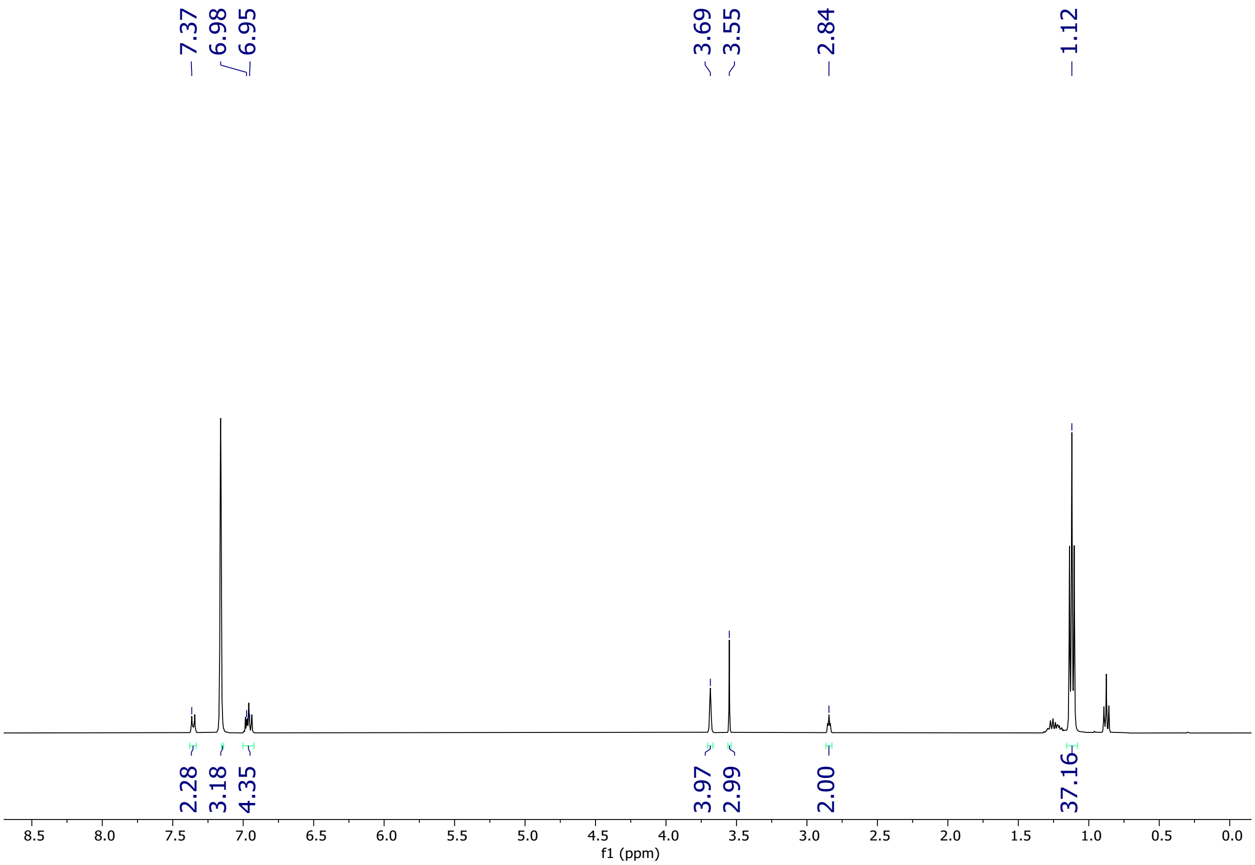

Figure S31. ^{1}H NMR spectrum of (^{tBu}PBP)Pd(CH_2-4-OMe-C_6H_4) in C_6D_6 at room temperature.

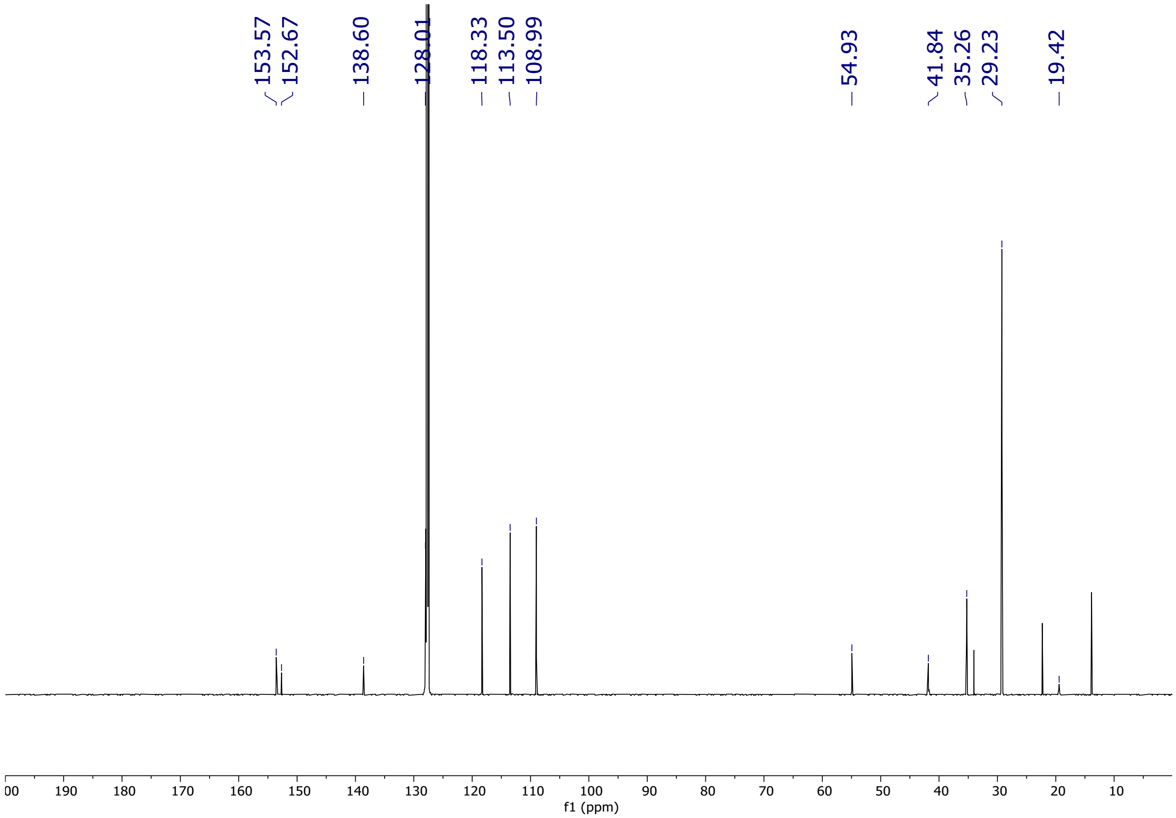

Figure S32. $^{13}C\{^1H\}$ NMR spectrum of $(^{tBu}PBP)Pd(CH_2\text{-}4\text{-}OMe\text{-}C_6H_4)$ in C_6D_6 at room temperature.

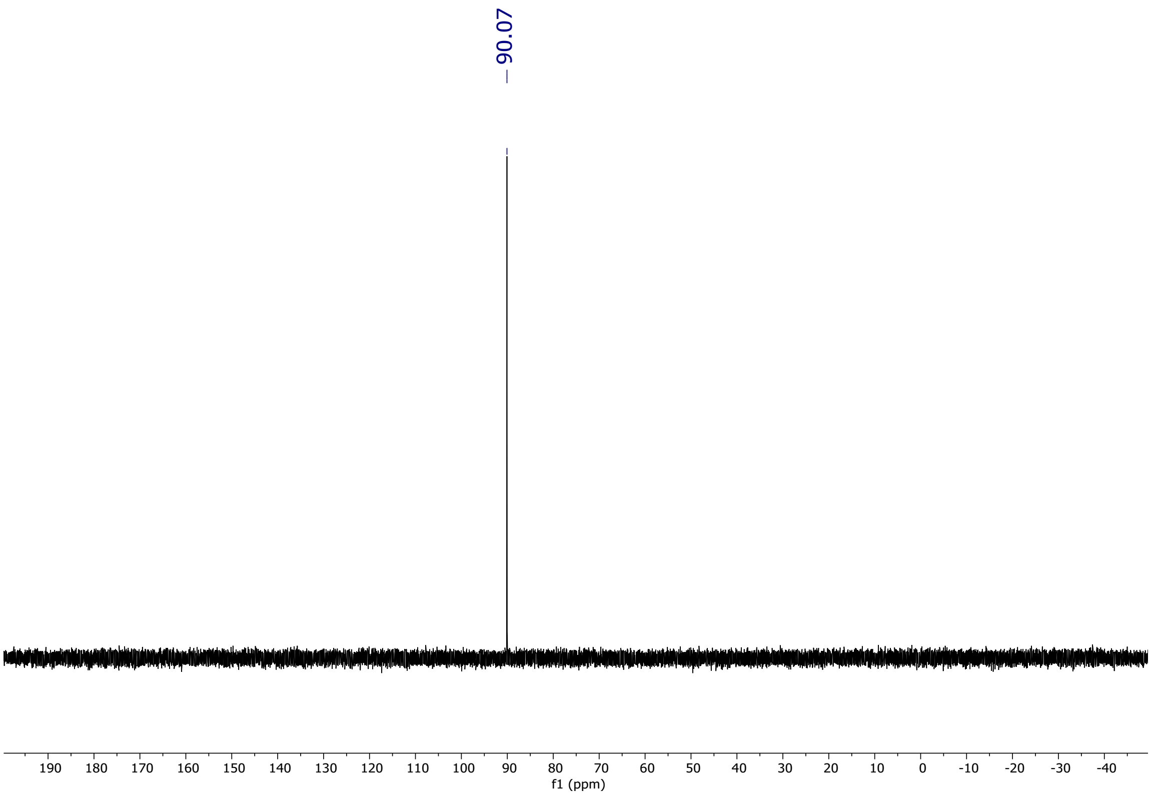

Figure S33. $^{31}P\{^1H\}$ NMR spectrum of $(^{tBu}PBP)Pd(CH_2\text{-}4\text{-}OMe\text{-}C_6H_4)$ in C_6D_6 at room temperature.

$(^{tBu}PB^{OMeBn}P)_2Pd_2$ (**2-^{OMe}Bn**)

NMR spectra of $(^{tBu}PB^{OMeBn}P)_2Pd_2$ at 25 °C in C_6D_6 are shown in Figures S34-S35.

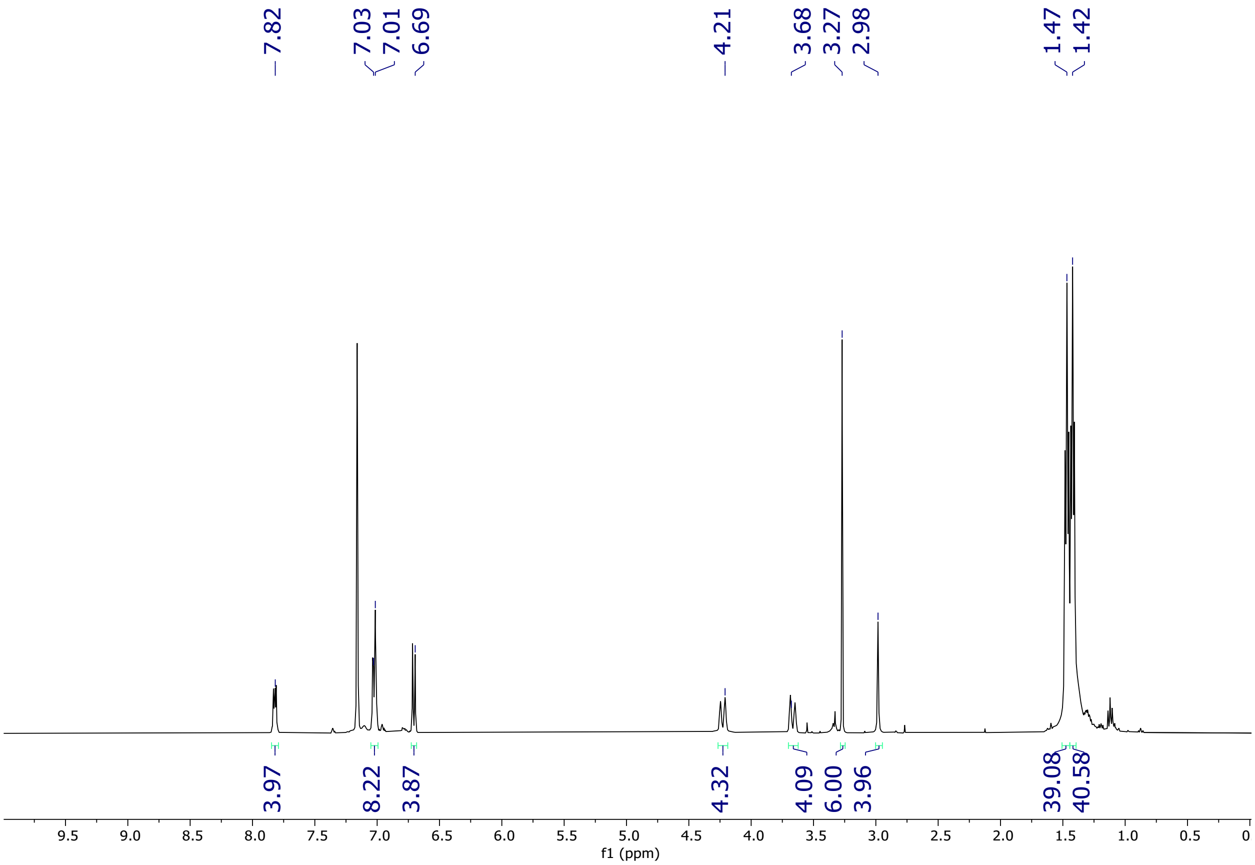

Figure S34. ^{1}H NMR spectrum of the thermal decomposition of $(^{tBu}PBP)Pd(CH_2\text{-4-OMe-}C_6H_4)$ after 3 days in C_6D_6 at 65 °C. The major peaks, which correspond to the Pd(0) dimer product, $(^{tBu}PBP^{OMeBn}P)_2Pd_2$, are picked and integrated.

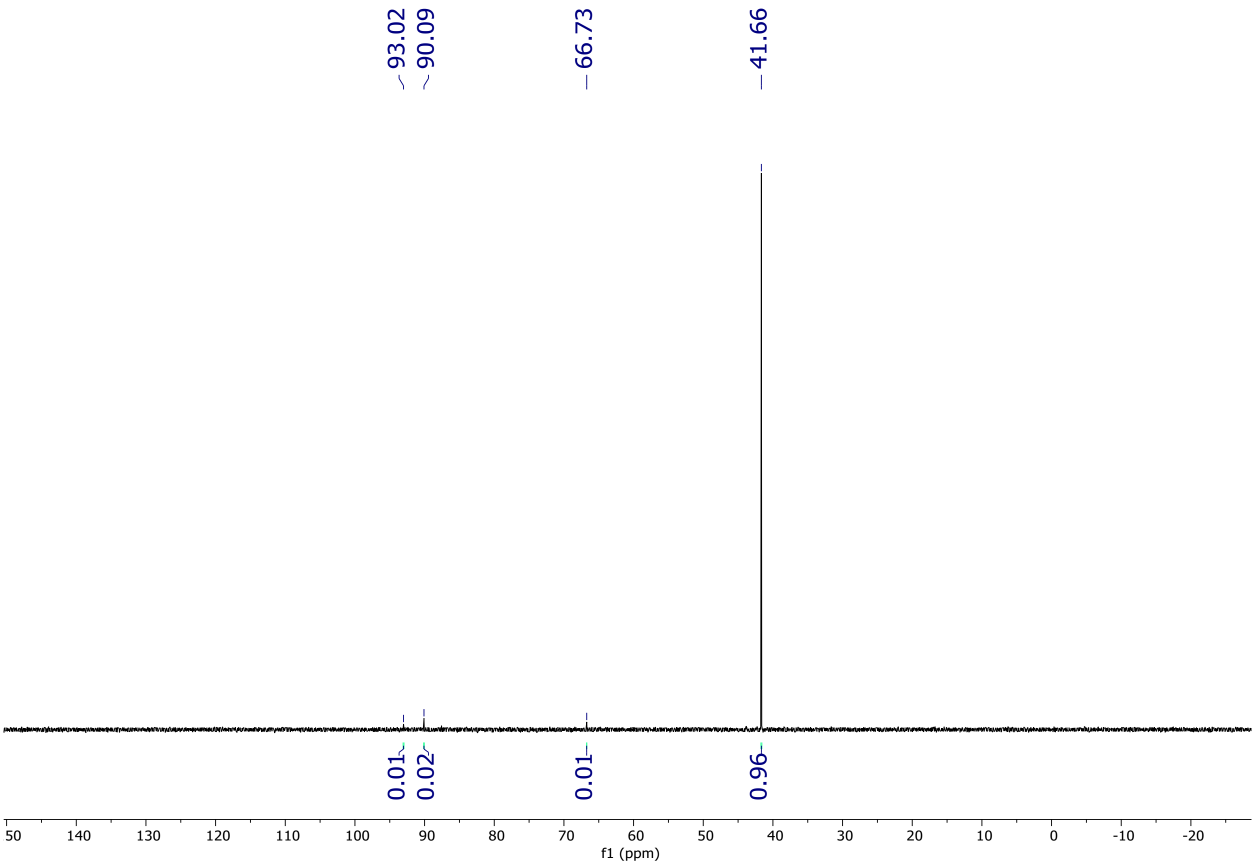

Figure S35. $^{31}P\{^{1}H\}$ NMR spectrum of the thermal decomposition of (^{tBu}PBP)Pd(CH_2-4-OMe-C_6H_4) after 3 days in C_6D_6 at 65 °C. The major peak is at 41.6 ppm, which corresponds to the Pd(0) dimer product, (^{tBu}PB^{OMeBn}P)$_2$Pd$_2$.

$(^{tBu}PBP)Pd(C_6H_5)$ (**1-Ph**)

NMR spectra for $(^{tBu}PBP)Pd(C_6H_5)$ at 25 °C in C_6D_6 are shown in Figures S36-S38.

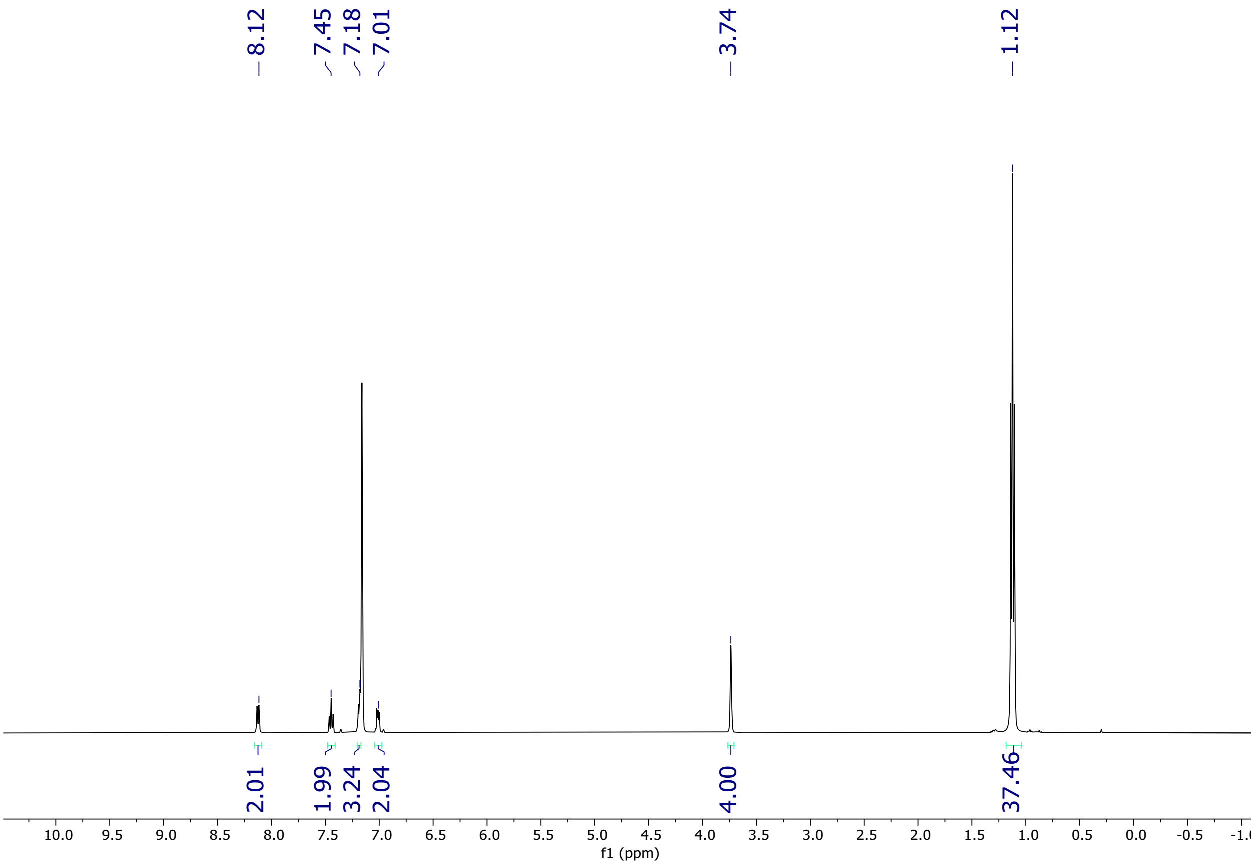

Figure S36. ^1H NMR spectrum of $(^{tBu}PBP)Pd(C_6H_5)$ in C_6D_6 at room temperature.

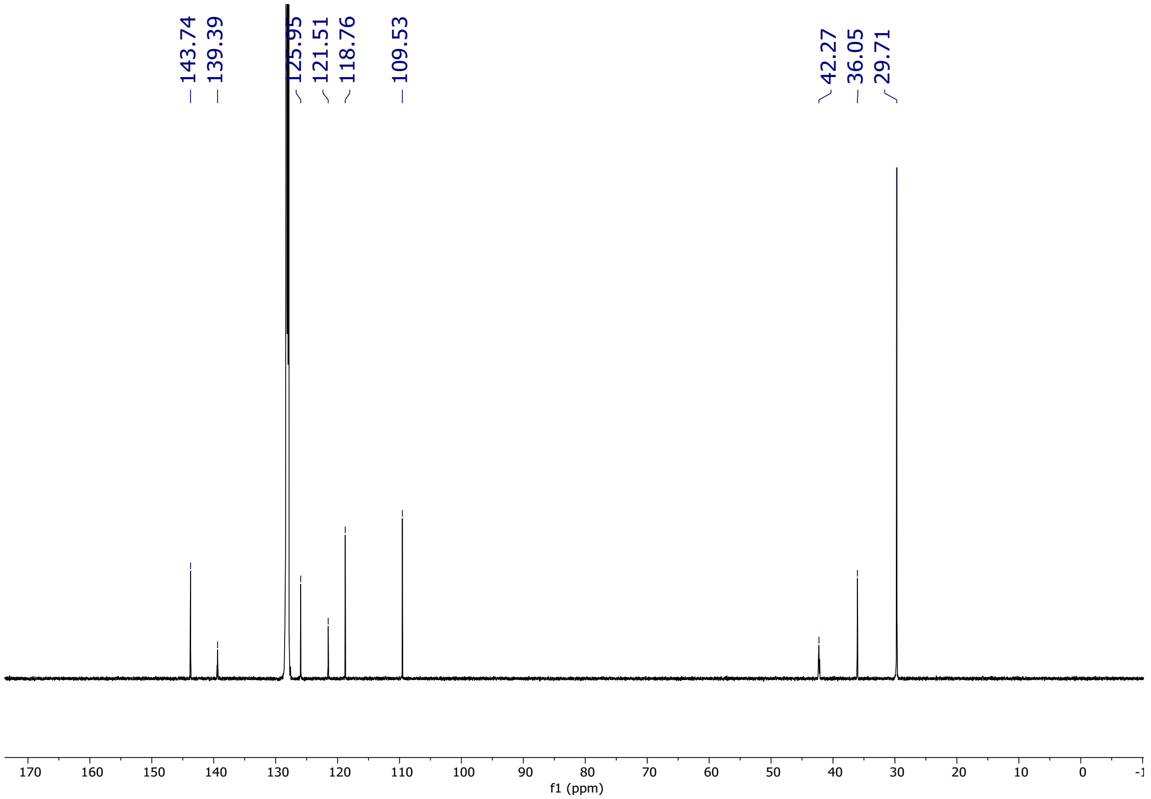

Figure S37. $^{13}C\{^{1}H\}$ NMR spectrum of (^{tBu}PBP)Pd(C_6H_5) in C_6D_6 at room temperature.

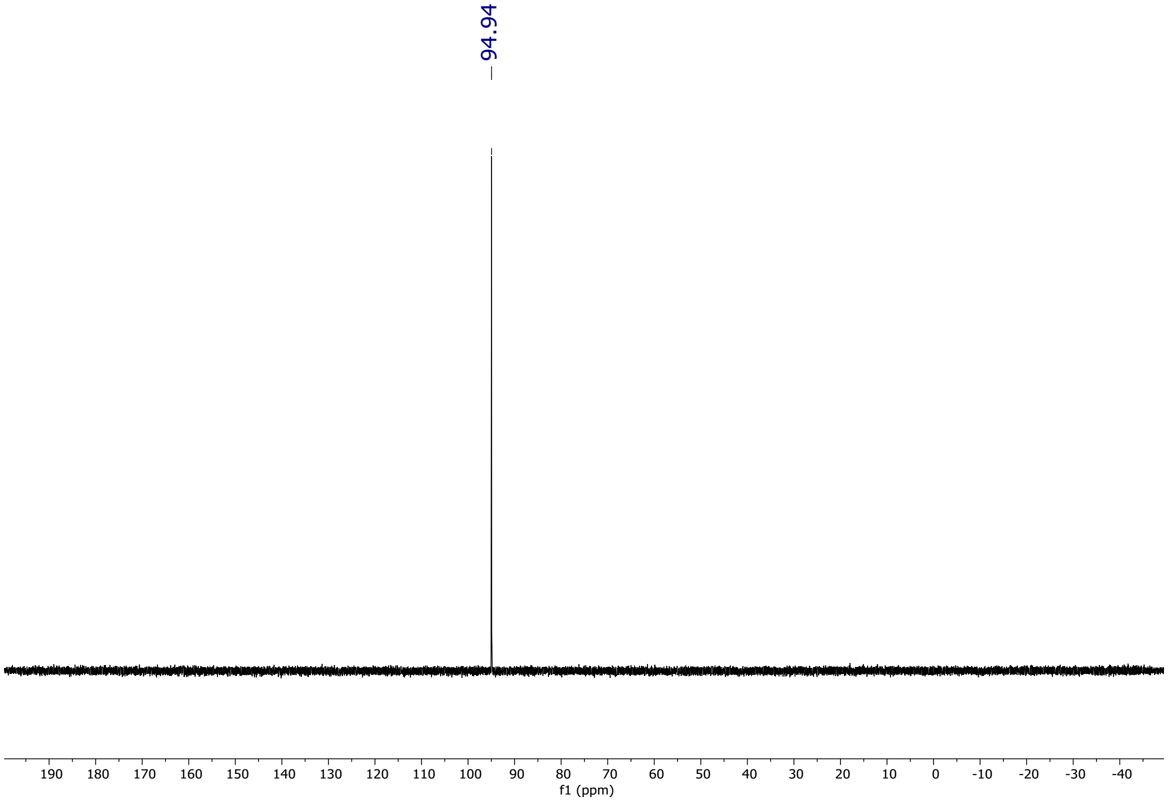

Figure S38. $^{31}P\{^{1}H\}$ NMR spectrum of (^{tBu}PBP)Pd(C_6H_5) in C_6D_6 at room temperature.

(^{tBu}PBP)Pd{OC(O)CH_2CH_3} (**3-Et**)

NMR and IR spectra for (^{tBu}PBP)Pd{OC(O)CH_2CH_3} at 25 °C in C_6D_6 are shown in Figures S39-S42.

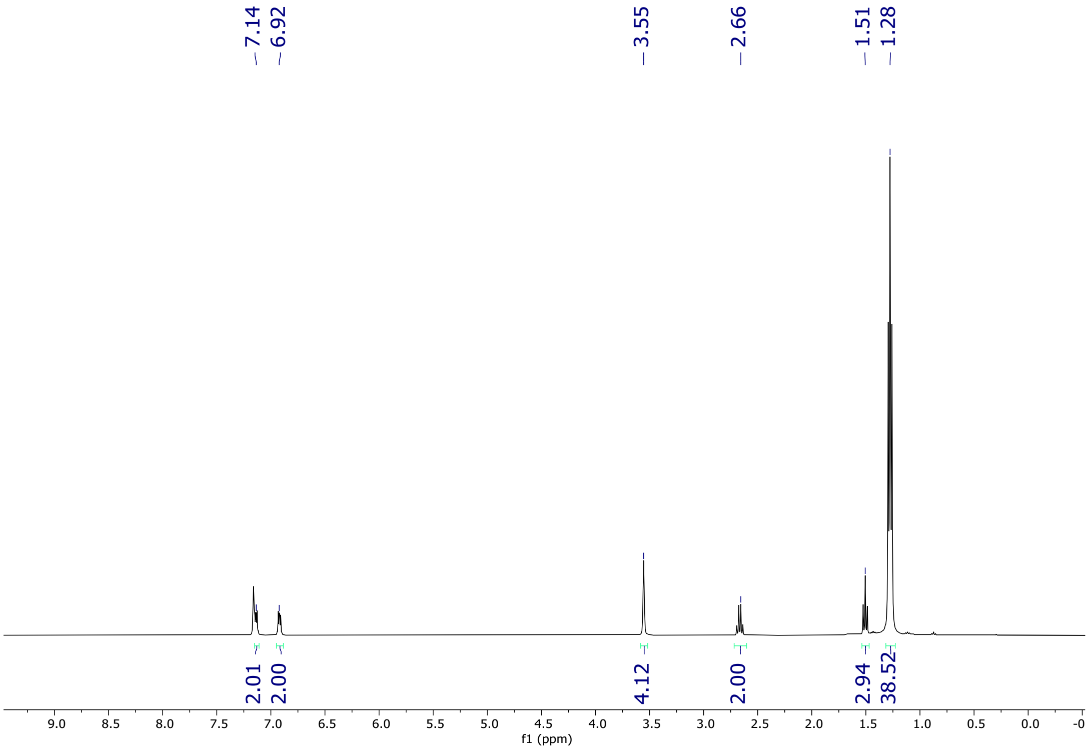

Figure S39. ^{1}H NMR spectrum of (^{tBu}PBP)Pd{OC(O)CH_2CH_3} in C_6D_6 at room temperature.

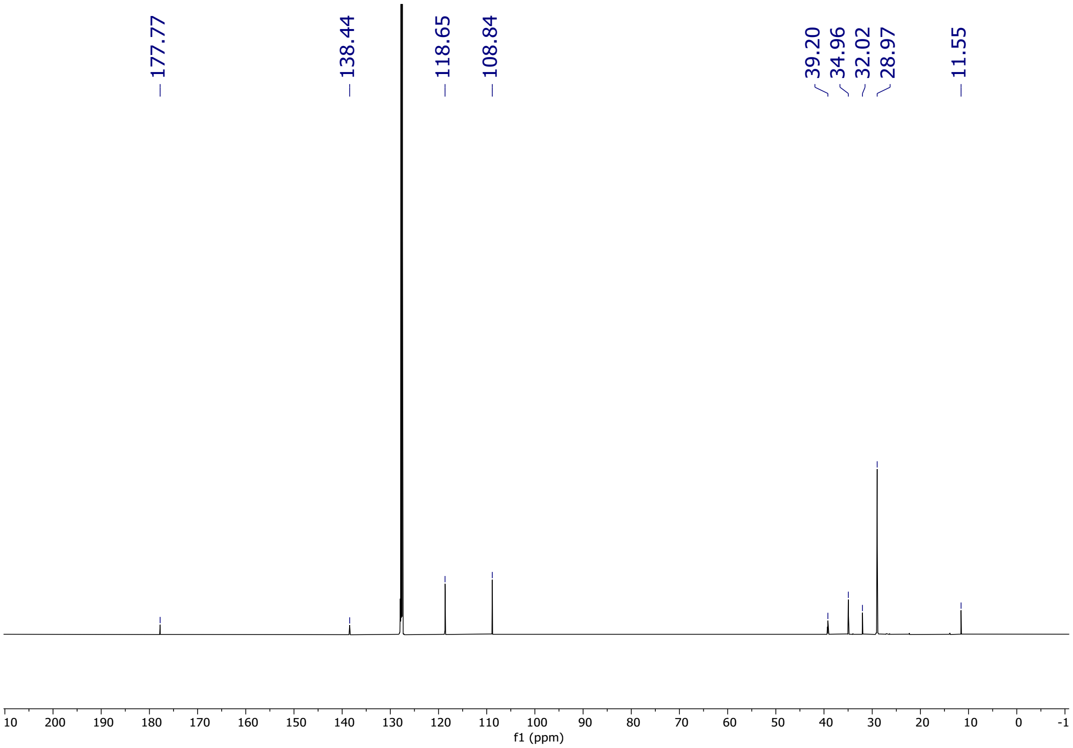

Figure S40. $^{13}C\{^{1}H\}$ NMR spectrum of (^{tBu}PBP)Pd{OC(O)CH_2CH_3} in C_6D_6 at room temperature.

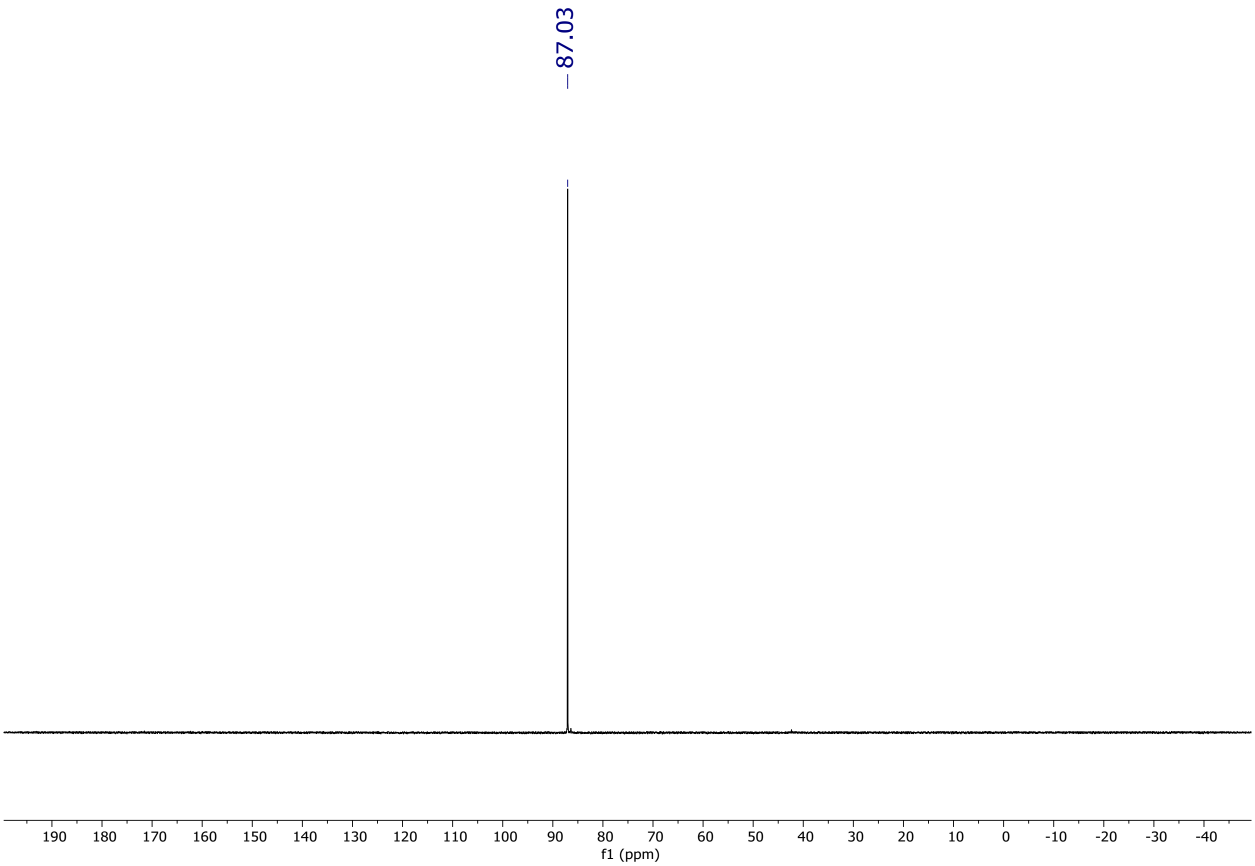

Figure S41. $^{31}P\{^{1}H\}$ NMR spectrum of (^{tBu}PBP)Pd{OC(O)CH_2CH_3} in C_6D_6 at room temperature.

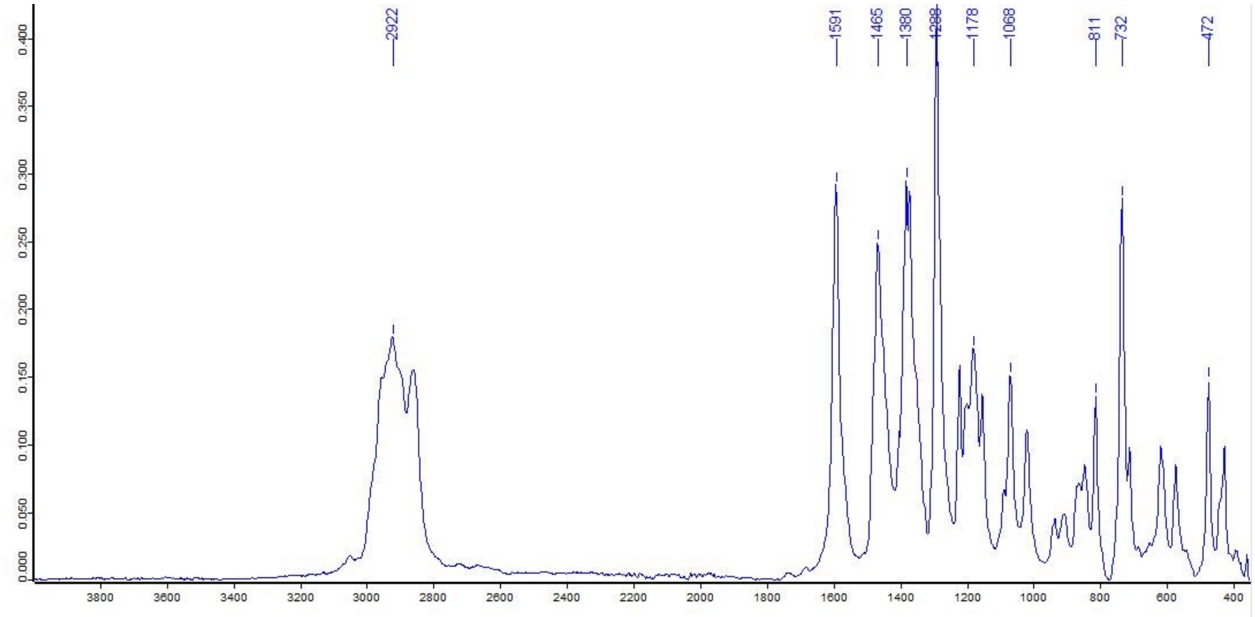

Figure S42. IR spectrum of (^{tBu}PBP)Pd{OC(O)CH_2CH_3}.

(^{tBu}PBP)Pd{OC(O)CH2CH2CH3} (**3-^{n}Pr**)

NMR and IR spectra for (^{tBu}PBP)Pd{OC(O)$CH_2CH_2CH_3$} at 25 °C in C_6D_6 are shown in Figures S43-S46.

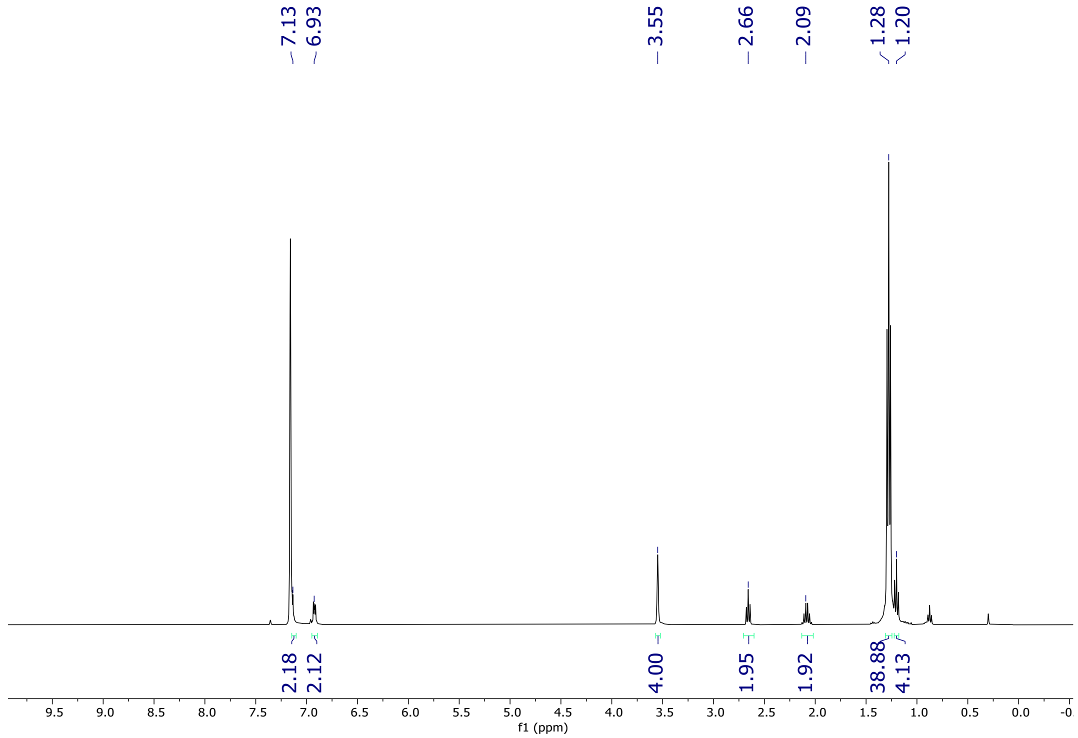

Figure S43. ^{1}H NMR spectrum of (^{tBu}PBP)Pd{OC(O)$CH_2CH_2CH_3$} in C_6D_6 at room temperature.

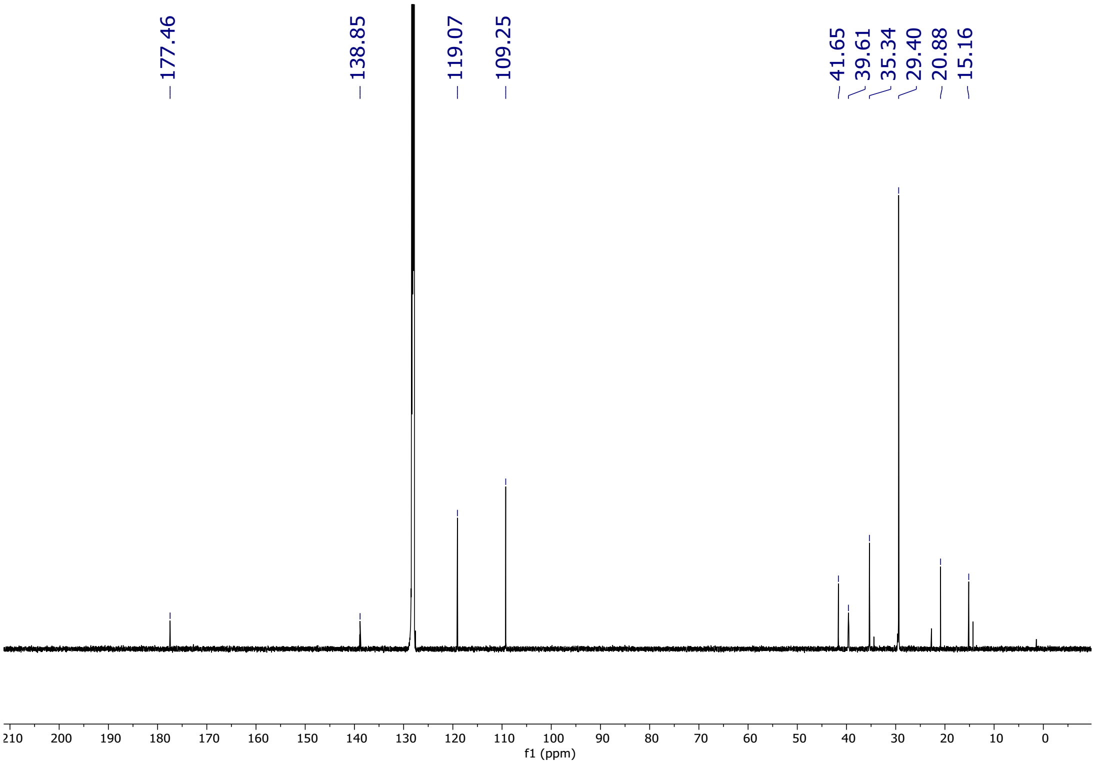

Figure S44. ^{13}C{^{1}H} NMR spectrum of (^{tBu}PBP)Pd{OC(O)$CH_2CH_2CH_3$} in C_6D_6 at room temperature.

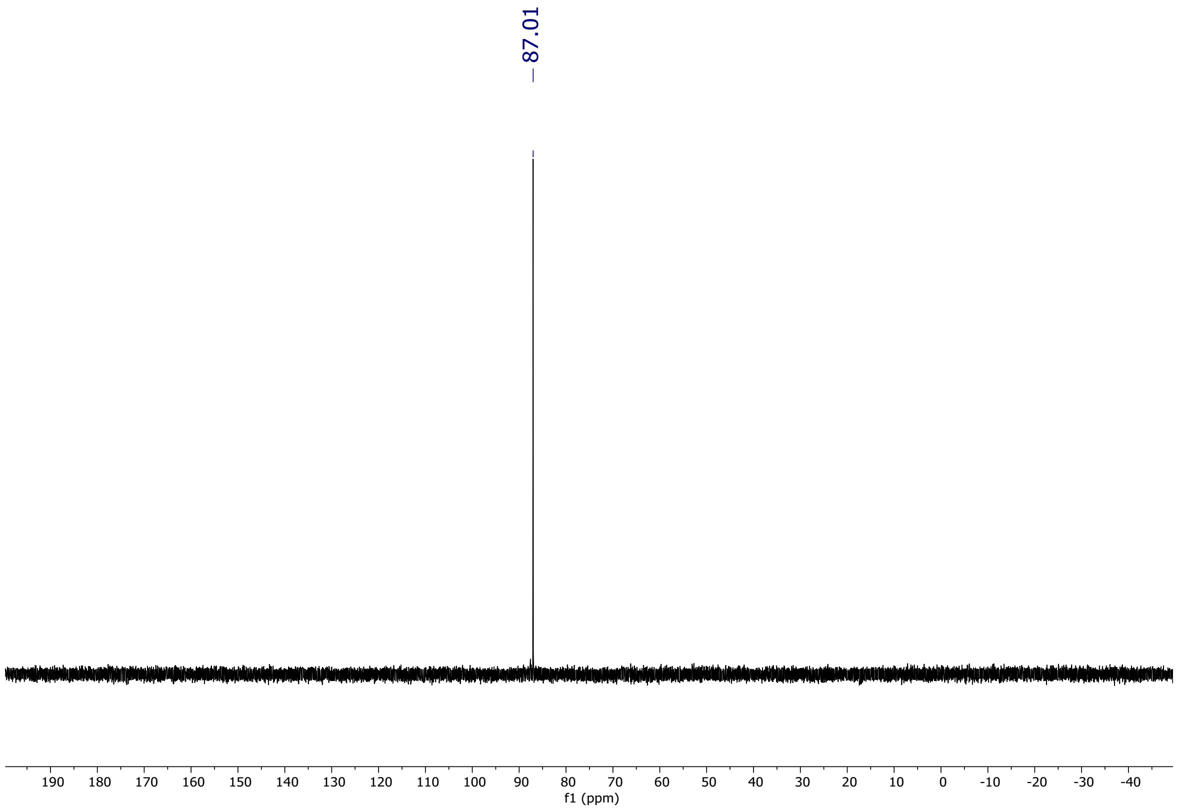

Figure S45. $^{31}P\{^{1}H\}$ NMR spectrum of (^{tBu}PBP)Pd{OC(O)$CH_2CH_2CH_3$} in C_6D_6 at room temperature.

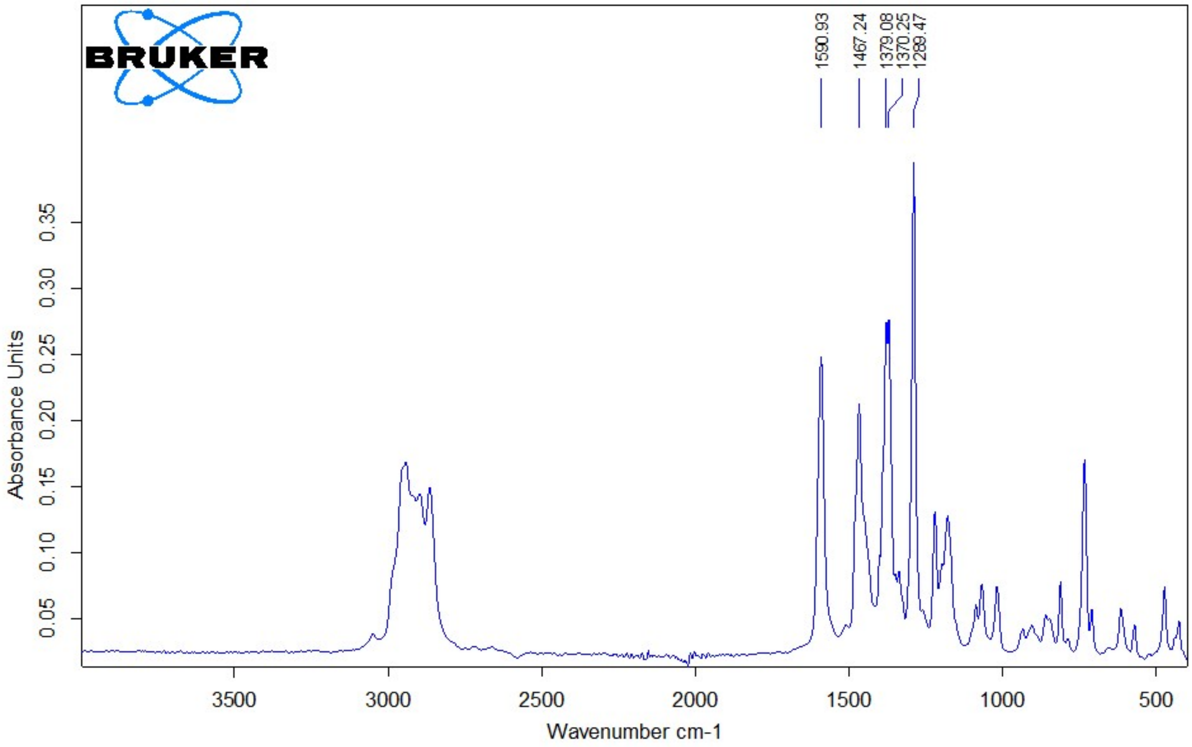

Figure S46. IR spectrum of (^{tBu}PBP)Pd{OC(O)$CH_2CH_2CH_3$}.

(^{tBu}PBP)Pd{OC(O)$CH_2C_6H_5$} (**3-Bn**)

NMR and IR spectra for (^{tBu}PBP)Pd{OC(O)$CH_2C_6H_5$} at 25 °C in C_6D_6 are shown in Figures S47-S50.

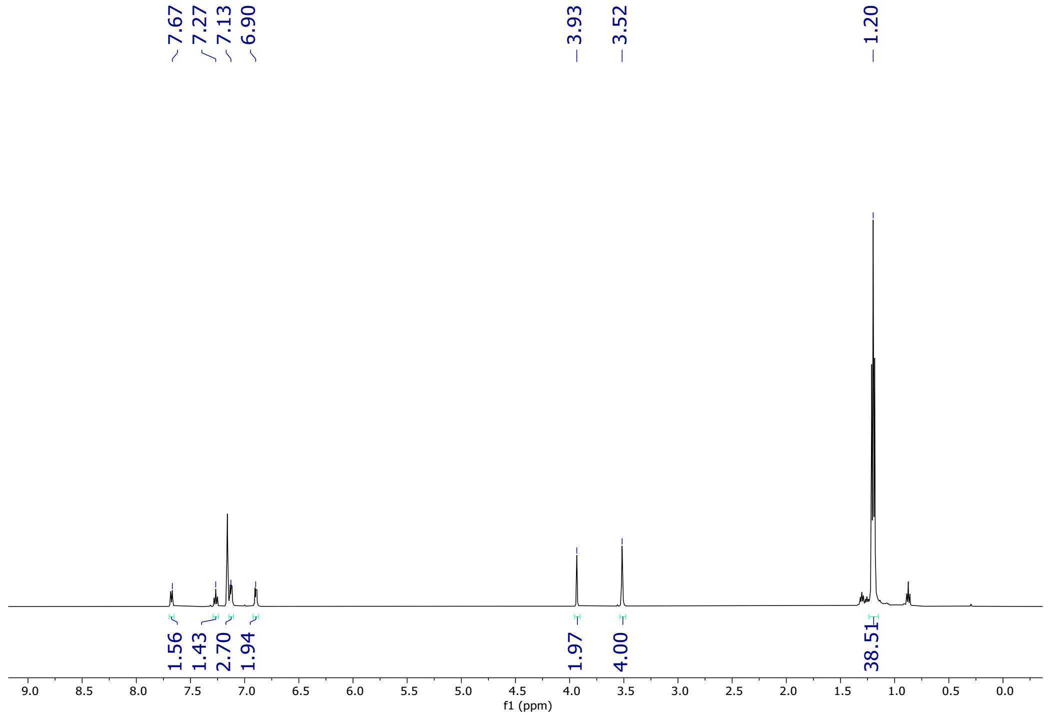

Figure S47. ^{1}H NMR spectrum of (^{tBu}PBP)Pd{OC(O)$CH_2C_6H_5$} in C_6D_6 at room temperature.

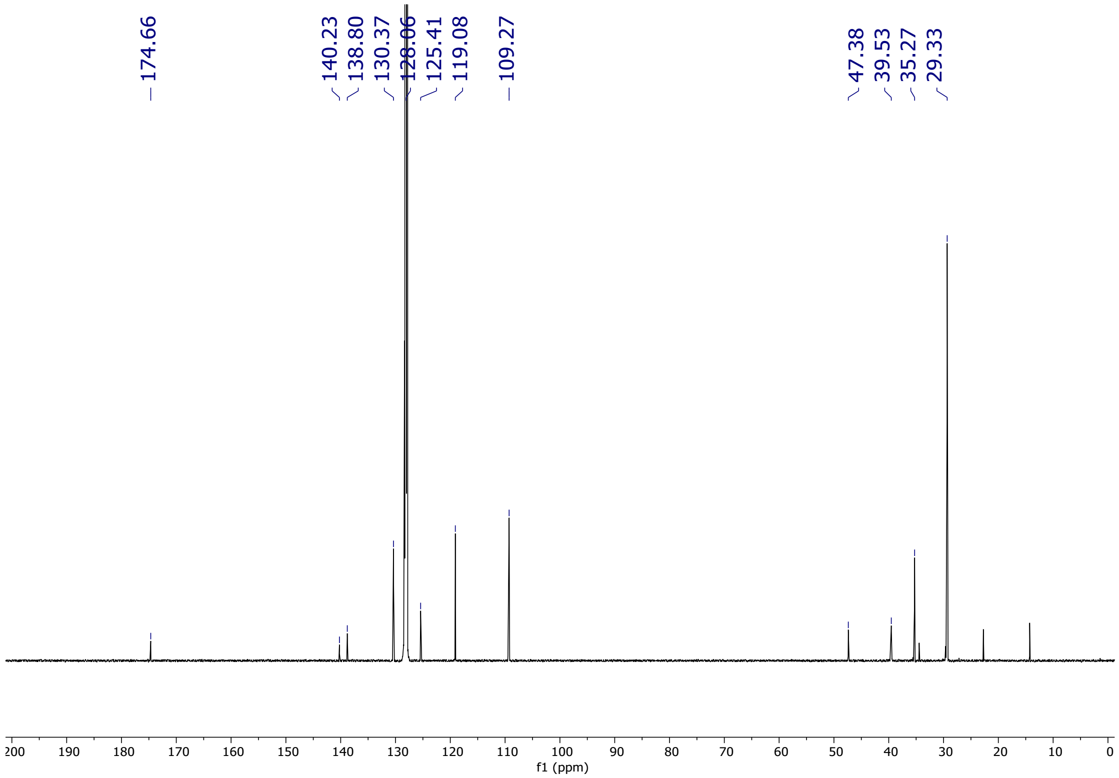

Figure S48. ${}^{13}C\{{}^{1}H\}$ NMR spectrum of (^{tBu}PBP)Pd{OC(O)$CH_2C_6H_5$} in C_6D_6 at room temperature.

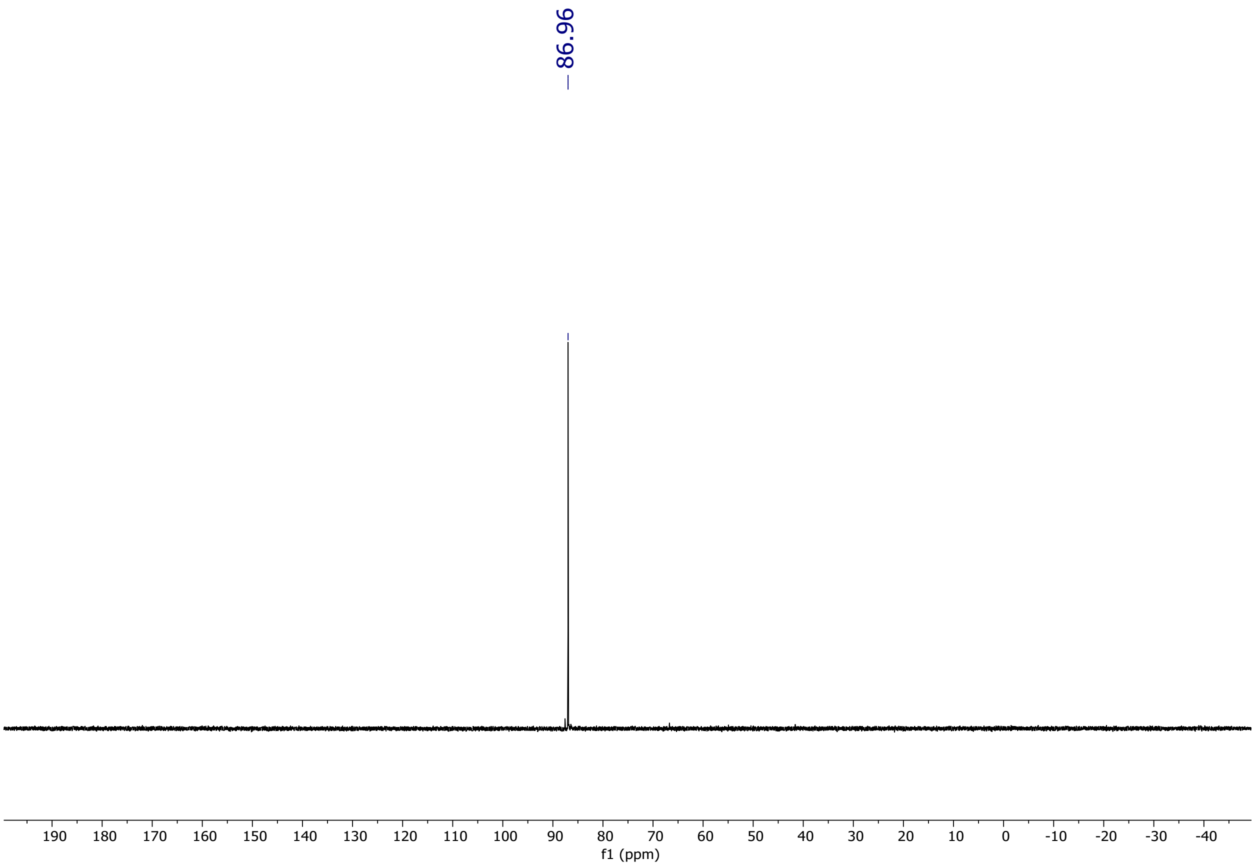

Figure S49. $^{31}P\{^{1}H\}$ NMR spectrum of (^{tBu}PBP)Pd{OC(O)$CH_2C_6H_5$} in C_6D_6 at room temperature.

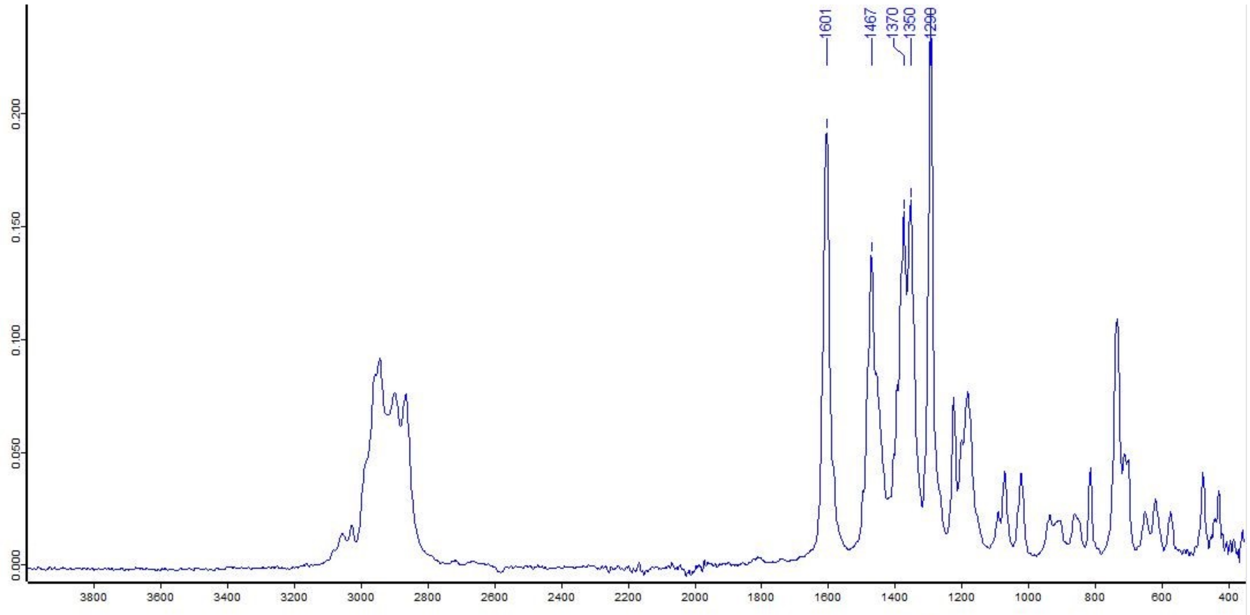

Figure S50. IR spectrum of (^{tBu}PBP)Pd{OC(O)$CH_2C_6H_5$}.

$(^{tBu}PBP)Pd\{OC(O)CH_2\text{-}4\text{-}OMe\text{-}C_6H_4\}$ (**3-^{OMe}Bn**)

NMR and IR spectra for $(^{tBu}PBP)Pd\{OC(O)CH_2\text{-}4\text{-}OMe\text{-}C_6H_4\}$ at 25 °C in C_6D_6 are shown in Figures S51-S54.

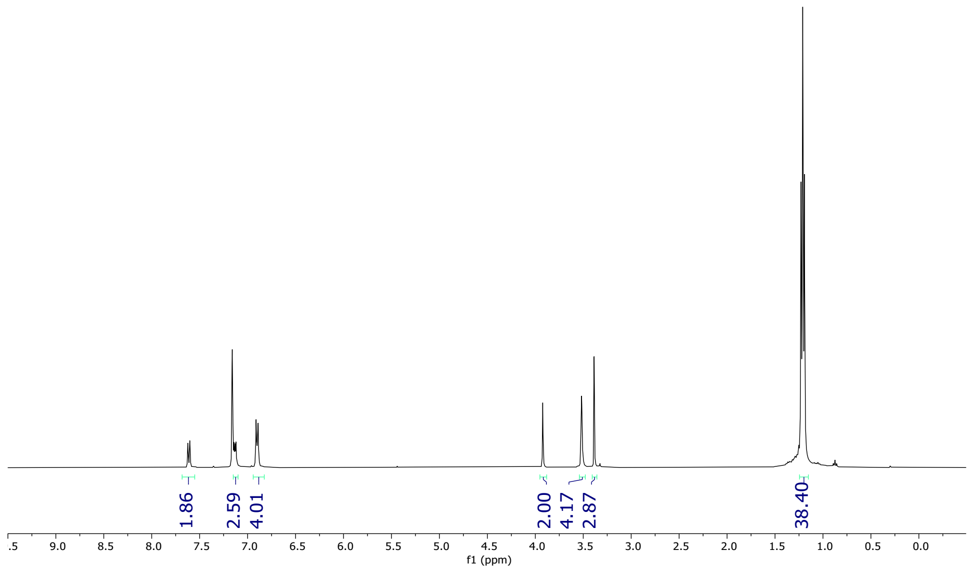

Figure S51. ^1H NMR spectrum of $(^{tBu}PBP)Pd\{OC(O)CH_2\text{-}4\text{-}OMe\text{-}C_6H_4\}$ in C_6D_6 at room temperature.

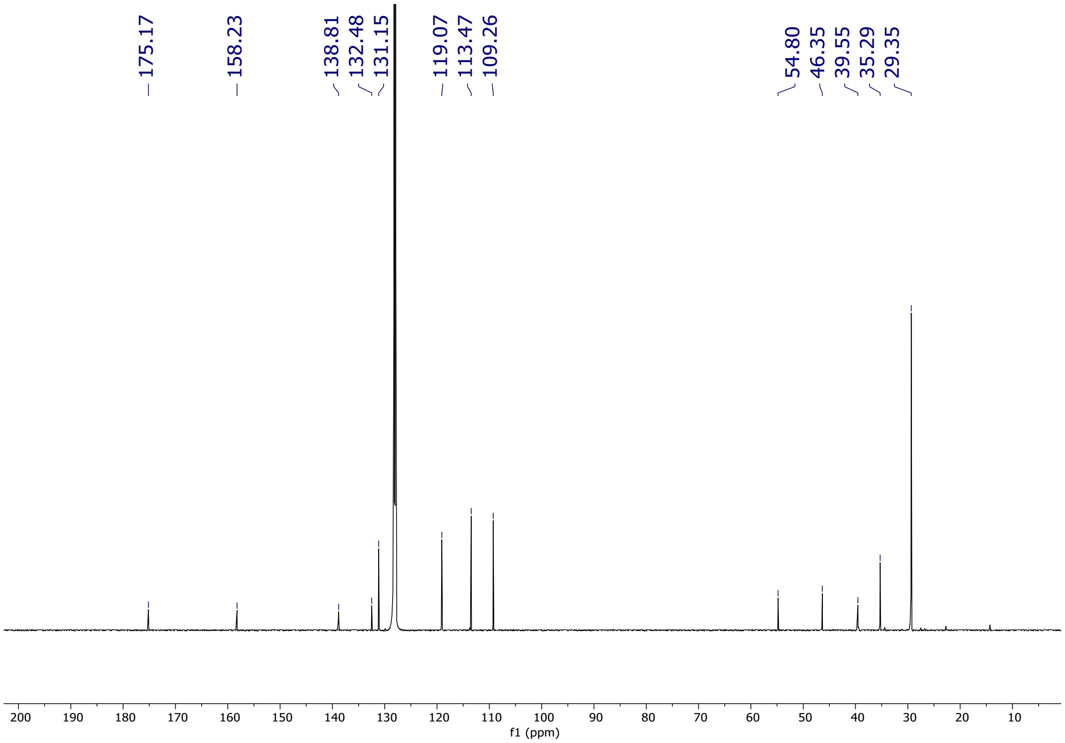

Figure S52. $^{13}C\{^1H\}$ NMR spectrum of $(^{tBu}PBP)Pd\{OC(O)CH_2\text{-}4\text{-}OMe\text{-}C_6H_4\}$ in C_6D_6 at room temperature.

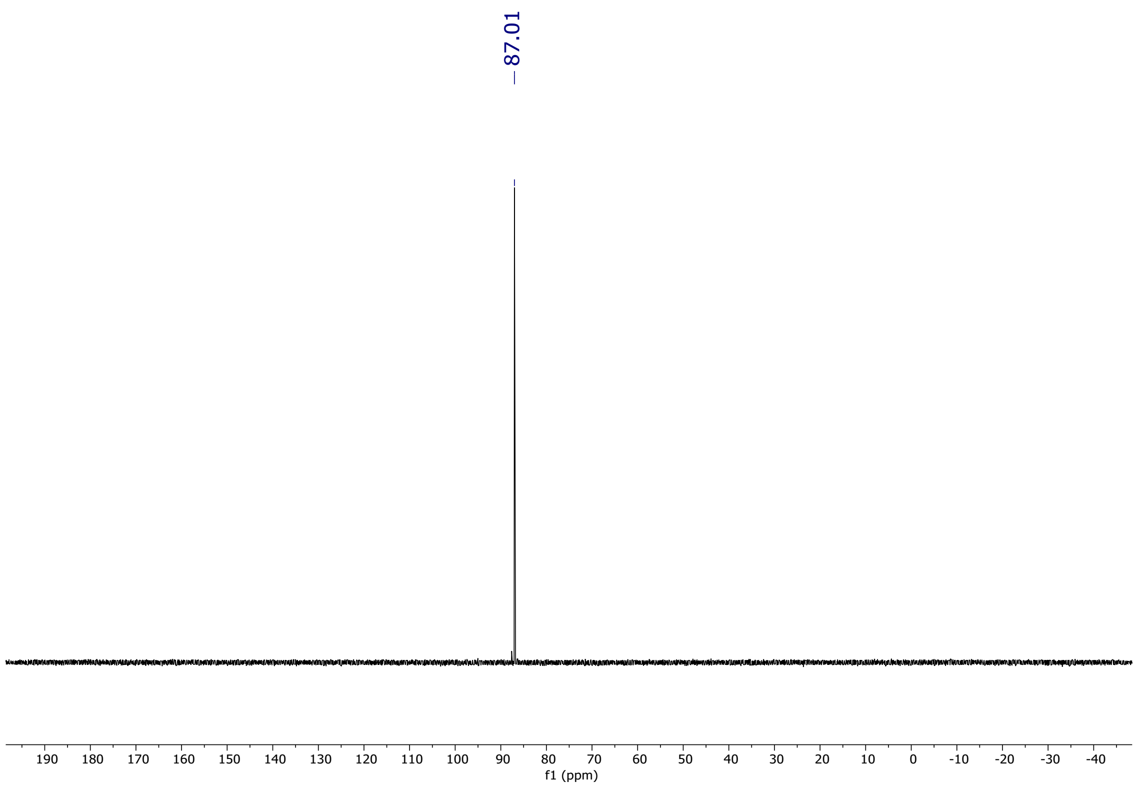

Figure S53. $^{31}P\{^{1}H\}$ NMR spectrum of (^{tBu}PBP)Pd{OC(O)CH_2-4-OMe-C_6H_4} in C_6D_6 at room temperature.

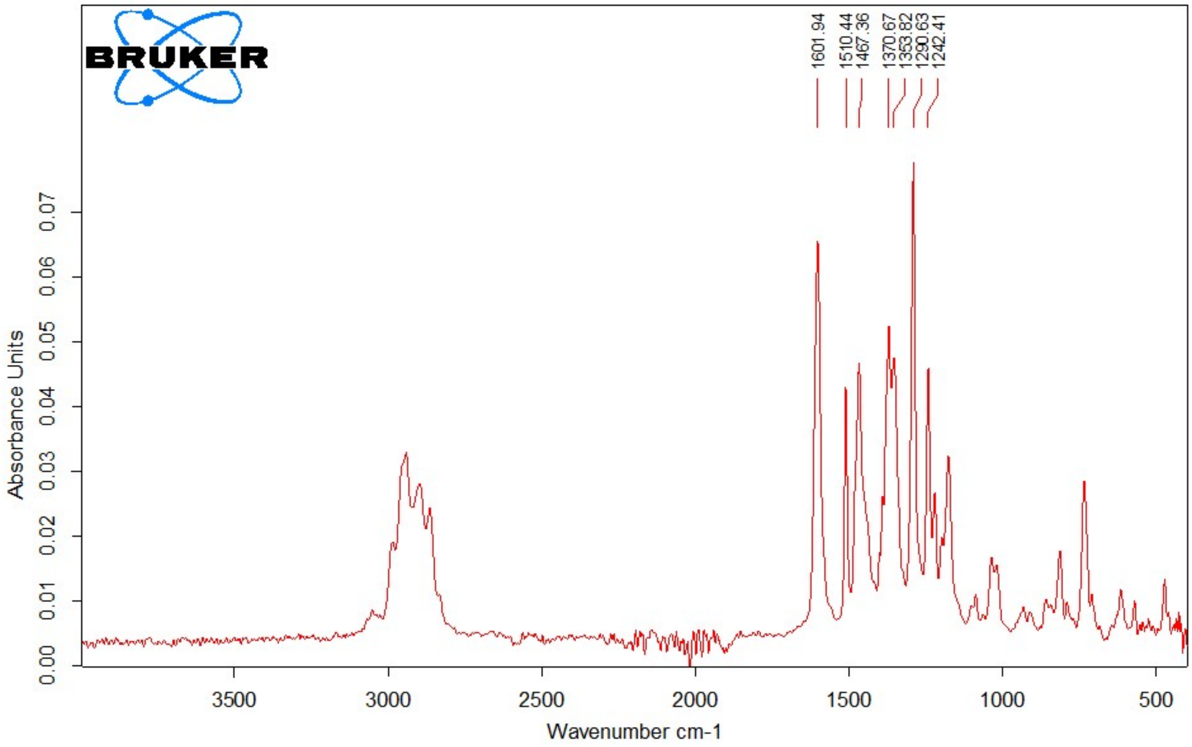

Figure S54. IR spectrum of (^{tBu}PBP)Pd{OC(O)CH_2-4-OMe-C_6H_4}.

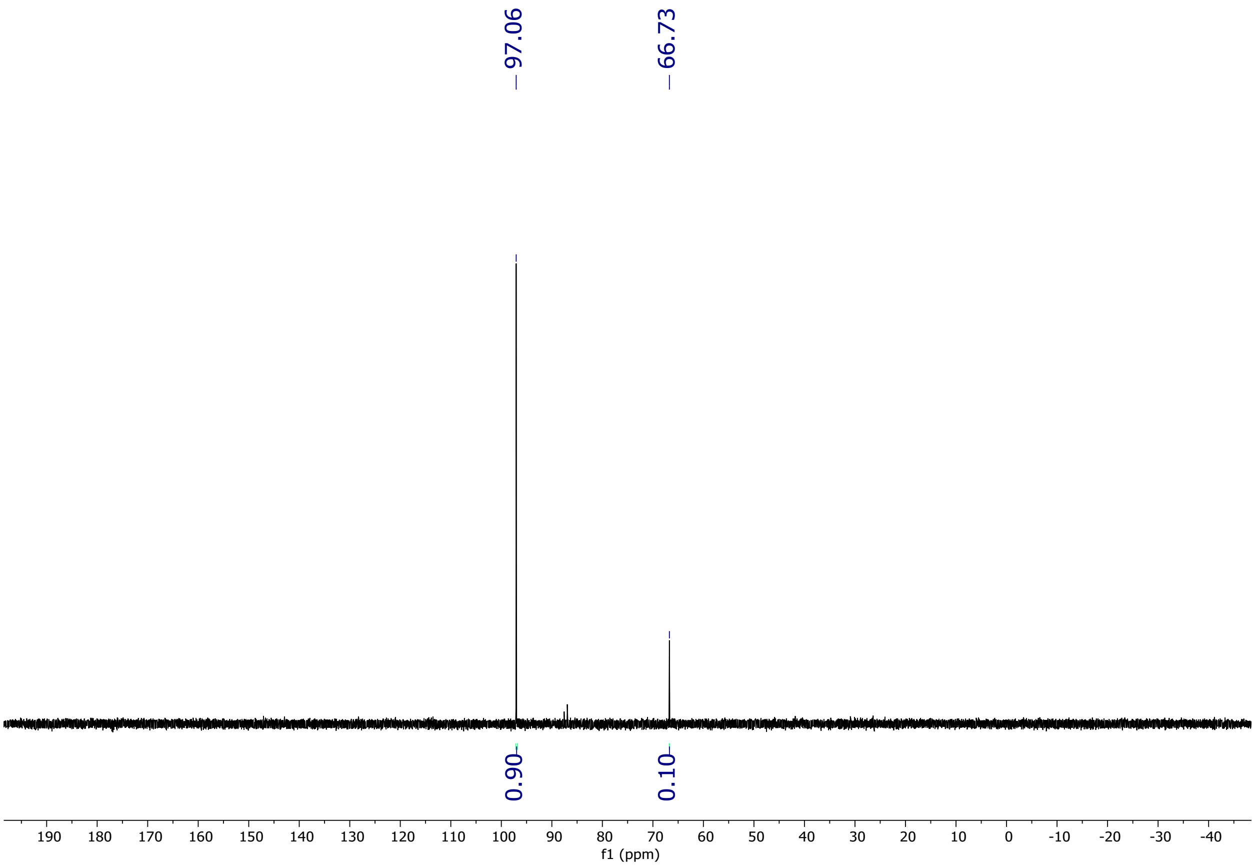

Figure S55. $^{31}P\{^1H\}$ NMR spectrum of the thermal decomposition of **1-Me** after 5 days at 65 °C. **1-Me** (97.06, 90% by integration) remains the major species and **2-Me** has started to grow in (66.73 ppm, 10% by integration)

SXI. Computational Details

All calculations were performed on complete molecular systems without any truncations using Gaussian16 (Revision B.01).[5] The systems were fully relaxed, and no symmetry constraints were imposed. For the geometry optimizations, the DFT hybrid functional PBE0,[6] was used along with the GD3BJ[7] dispersion correction. Basis set BS1 was used for geometry optimizations, which comprises the SDD[8] basis set and effective core potential (ECP)[9] for palladium and def2-SVP[10] for other elements. To correct the electronic energies, single point calculations were performed using BS2 basis set, consisting of the SDD basis set with an additional f basis function and effective core potential (ECP) for palladium and def2-TZVPPD[8] for other elements. Solvation effects were included in both geometry optimizations and single point calculations using the polarizable continuum model (IEFPCM)[11] with the parameters of benzene ($\varepsilon = 2.27$). Additional calculations with wB97XD[12], B3LYP-D3,[11] and TPSSh[13] functionals provide similar trends as the PBE0-D3BJ level of theory (see Tables S2 and S3). For the CO_2 insertion TS and carboxyl intermediate, counterpoise (CP) corrections were computed at the BS2 level of theory. Reported Gibbs free energies include thermal corrections computed at 298 K and a standard-state (SS) correction to 1 M (1.89 kcal/mol at 298 K). The standard state Gibbs free energies ($\Delta G°_{1M,298K}$) reported correspond to:

$$\Delta G°_{1M,298K} = \Delta G_{1atm,\ 298K,BS1} - \Delta E_{1atm,BS1} + \Delta E_{1atm,BS2} + CP_{BS2} + SS_{298K}$$

The Pd-hydride complex (L_nPd-H, in Figure 10) had convergence issues while optimizing with IEFPCM solvation model. The complex was optimized with CPCM solvation model and a single point energy was computed with the IEFPCM model. This convergence problem may be due to solvent cavity formation as it was able to optimize in the gas phase.

Complex	PBE0-D3BJ			ωB97XD		
	TS1_Inn	TS1_Out	Difference (kcal/mol)	TS1_Inn	TS1_Out	Difference (kcal/mol)
(^{tBu}PBP)Pd(CH_3) (**1-Me**)	25.0	19.4	5.6	28.4	25.4	3.0
(^{tBu}PBP)Pd(CH_3) (**1-Et**)	26.0	17.7	8.3	28.8	23.1	5.7
(^{tBu}PBP)Pd($CH_2CH_2CH_3$) (**1-^{n}Pr**)	26.5	21.8	4.7	29.3	n.d.	-
(^{tBu}PBP)Pd(C_6H_5) (**1-Ph**)	34.6	n.d.	-	37.0	n.d.	-
(^{tBu}PBP)Pd($CH_2C_6H_5$) (**1-Bn**)	31.3	20.3	11.0	35.1	24.7	10.4
(^{tBu}PBP)Pd(CH_2-4-OMe-C_6H_4) (**1-^{OMe}Bn**)	30.8	20.5	10.3	34.8	24.7	10.1
(^{tBu}PBP)Pd(CH_2-4-CF_3-C_6H_4) (**1-^{CF3}Bn**)	n.d.	20.6	-	35.9	26.5	9.4
(^{Me}PBP)Pd(CH_3)	17.5	20.0	-2.5	21.3	24.1	-2.8
(^{Me}PBP)Pd(CH_2CH_3)	17.3	17.3	0.0	n.d.	21.5	-

Table S2. Computed barriers for inner- and outer- sphere C-CO_2 bond formation with different complexes using PBE0-D3BJ and ωB97XD levels of theory. (n.d. = TS could not be optimized)

Complex	B3LYP-D3BJ			TPSSh		
	TS1_Inn	TS1_Out	Difference (kcal/mol)	TS1_Inn	TS1_Out	Difference (kcal/mol)
(^{tBu}PBP)Pd(CH_3) (**1-Me**)	14.2	11.3	2.9	21.4	15.3	6.1
(^{tBu}PBP)Pd(CH_3) (**1-Et**)	15.9	9.6	6.3	22.9	14.9	8.0
(^{tBu}PBP)Pd($CH_2CH_2CH_3$) (**1-^{n}Pr**)	15.9	11.4	4.5	23.4	18.7	4.7
(^{tBu}PBP)Pd($CH_2C_6H_5$) (**1-Bn**)	19.6	12.2	7.7	27.7	18.2	9.5

Table S3. Single-point computed energy barriers for inner and outer sphere C-CO_2 bond formation with different complexes using B3LYP-D3 and TPSSh levels of theory.

SXII. X-ray Diffraction Data

General X-ray Experimental

Low-temperature diffraction data (ω-scans) were collected on either a Rigaku MicroMax-007HF diffractometer coupled to a Dectris Pilatus3R detector with Mo Kα (λ = 0.71073 Å), a MicroMax-007HF diffractometer coupled to a Saturn994+ CCD detector with Cu Kα (λ = 1.54178 Å), Rigaku xtaLAB mini II diffractometer coupled to a HyPix-Bantam hybrid photon counting X-ray detector with Mo Kα (λ = 0.71073 Å), or a Rigaku Synergy-S diffractometer coupled to a HyPix-Arc 100 detector with Cu Kα (λ = 1.54178 Å). The diffraction images were processed and scaled using Rigaku Oxford Diffraction software (CrysAlisPro; Rigaku OD: The Woodlands, TX, 2015). The structure was solved with SHELXT and was refined against F^2 on all data by full-matrix least squares with SHELXL.[14] All non-hydrogen atoms were refined anisotropically. Unless stated otherwise, hydrogen atoms were included in the model at geometrically calculated positions and refined using a riding model. The isotropic displacement parameters of all hydrogen atoms were fixed to 1.2 times the U value of the atoms to which they are linked (1.5 times for methyl groups). The full numbering scheme of all submitted compounds can be found in the full details of the X-ray structure determination. CCDC numbers 2243696 (007b-22021), 2243697 (syn-23013_auto), 2243698 (mini2-22007), 2243699 (mini2-22012), 2243700 (007a-22028), 2243701 (007a-23009), 2243702 (mini2-22009), 2243703 (mini2-22004) and 2264960 (syn-23079) contain the supplementary crystallographic data for this paper. These data can be obtained free of charge from The Cambridge Crystallographic Data Center via www.ccdc.cam.ac.uk/data_request/cif.

X-ray special details for (^{tBu}PBP)Pd(CH_2CH_3) (CCDC: 2243696)

The difference map has large negative peaks around the palladium atom. These peaks are likely from either Fourier truncations or are artifacts created by the spherical form factor.

X-ray special details for (^{tBu}PBP)Pd($CH_2CH_2CH_3$) (CCDC: 2243697)

All information is included in the general x-ray experimental section: no special refinement details.

X-ray special details for (^{tBu}PBP)Pd($CH_2C_6H_5$) (CCDC: 2243698)

All information is included in the general x-ray experimental section: no special refinement details.

X-ray special details for (^{tBu}PBP)Pd(CH_2-4-OMe-C_6H_4) (CCDC: 2243699)

All information is included in the general x-ray experimental section: no special refinement details.

X-ray special details for ($^{tBu}PB^{Bn}P)_2Pd_2$ (CCDC: 2243701)

Data was refined as a 2-component twin. The fractional volume of the second component was refined to 0.1978(14). The twin law found was (1 0 0 0 -1 0 -1 0 -1). The HKLF5 was generated with the program suite PLATON.[15] Several reflections were omitted due to detector artifacts. The relatively large Q peaks in the difference map (both positive and negative) around the palladium are likely due to artifacts created through using a spherical form factor.

X-ray special details for ($^{tBu}PB^{H}P)_2Pd_2$ (CCDC: 2243702)

The hydrogen on the boron atom was found in the difference map and allowed to freely refine (Figure S53.) The relatively large Q peaks in the difference map (both positive and negative) around the palladium are likely due to artifacts created through using a spherical form factor.

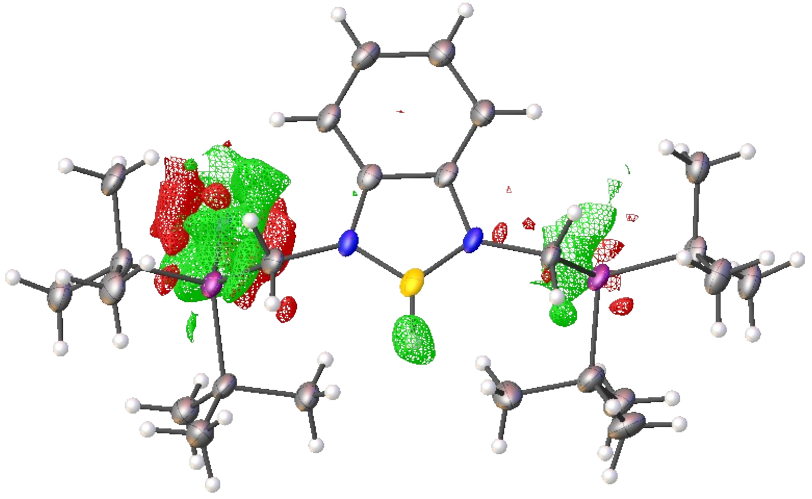

Figure S56. Difference map of the asymmetric unit. The hydrogen atom can be seen by the positive electron density near the Boron atom.

X-ray special details for ($^{tBu}PBP)Pd(C_6H_5)$ (CCDC: 2243700)

Disorder of a tert-butyl group was modeled as a two-part system with restraints of chemically equivalent bond distances and equivalent anisotropic atomic displacement parameters (U). The two parts were allowed to refine such that the total occupancy of the parts does not exceed 1. Due to instrumentation limits, five bad reflections were omitted. A high-resolution cut-off command was used to ignore reflections above 0.84 Angstroms. One low angle reflection was omitted from the least square refinement. This reflection was obscured by the beam stop.

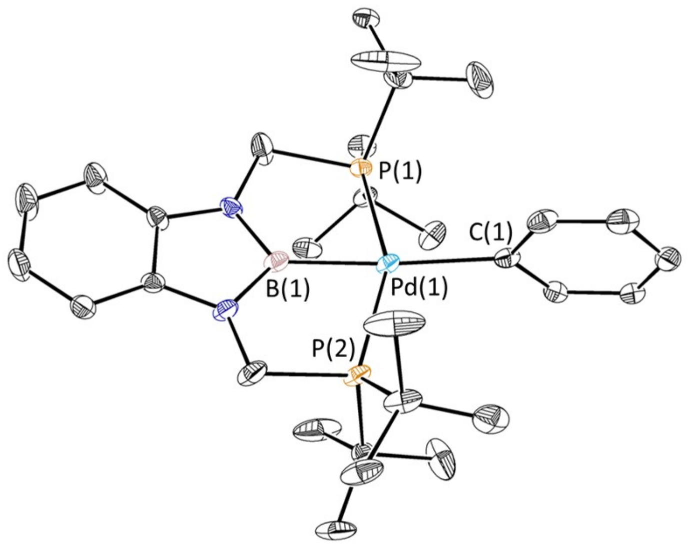

Figure S57. Solid-state structure of **1-Ph** with thermal ellipsoids at 30% probability. Hydrogen atoms are omitted for clarity. Selected distances (Å) and angles (°): Pd(1)-C(1) 2.162(3), Pd(1)-B(1) 2.022(4), Pd(1)-P(1) 2.3231(9), Pd(1)-P(2) 2.3265(8), B(1)-Pd(1)-C(1) 171.25(14), B(1)-Pd(1)-P(1) 77.01(11), B(1)-Pd(1)-P(2) 77.13(11), C(1)-Pd(1)-P(1) 102.18(9), C(1)-Pd(1)-P(2) 104.67(9), P(1)-Pd(1)-P(2) 152.78(3).

X-ray special details for (^{tBu}PBP)Pd{OC(O)CH_2CH_3} (CCDC: 2243703)

All information is included in the general x-ray experimental section: no special refinement details.

X-ray special details for (^{tBu}PB^{All}P)$_2$Pd$_2$ (CCDC: 2264960)

The data was refined as a 2-component twin. The fractional volume of the second component was refined to 0.4284(7). The twin law found was a ~180° rotation about the b-axis. Two reflections were omitted due to detector artifacts.

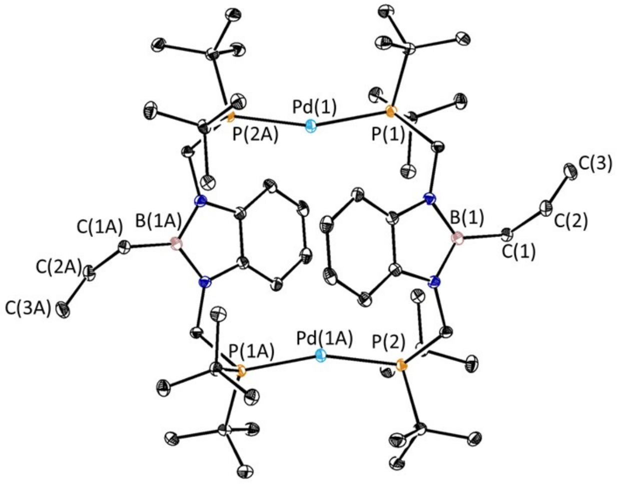

Figure S58. Solid-state structure of **2-All** with thermal ellipsoids at 30% probability. Hydrogen atoms and solvent are omitted for clarity. Selected distances (Å) and angles (°): Pd(1)-P(1) 2.2825(8), Pd(1)-P(2A) 2.2838(8), P(1)-Pd(1)-P(2A) 162.32(3), C(1)-C(2) 1.500(5), C(2)-C(3) 1.304(5).

Compound	$(^{tBu}PBP)Pd(CH_2C_6H_5)$	$(^{tBu}PBP)Pd(CH_2\text{-}4\text{-}OMe\text{-}C_6H_4)$	$(^{tBu}PBP)Pd(CH_2CH_2CH_3)$	$(^{tBu}PBP)Pd\{OC(O)CH_2CH_3\}$
**CCDC**	2243698	2243699	2243697	2243703
**Identification Code**	mini2-22007	mini2-22012	syn-23013_auto	mini2-22004
**Empirical Formula**	$C_{31}H_{51}BN_2P_2Pd$	$C_{37}H_{65}BN_2OP_2Pd$	$C_{27}H_{51}BN_2P_2Pd$	$C_{27}H_{49}BN_2O_2P_2Pd$
**Wavelength (Å)**	0.71073	0.71073	1.54184	0.71073
**Temperature (K)**	93(2)	93(2)	93(2)	93(2)
**FW**	630.88	733.06	582.84	612.83
**Crystal System**	monoclinic	monoclinic	triclinic	monoclinic
**Space Group**	$P2_1/n$	$P2_1/n$	$P\bar{1}$	$P2_1/n$
***a* (Å)**	12.9206(9)	14.9723(7)	7.85480(10)	11.8927(7)
***b* (Å)**	17.1954(13)	11.4923(6)	11.08790(10)	19.9252(9)
***c* (Å)**	14.8970(10)	23.5265(14)	17.8239(2)	13.4943(8)
**α (º)**	90	90	104.2180(10)	90
**β (º)**	103.790(7)	105.439(6)	94.7470(10)	112.762(7)
**γ (º)**	90	90	100.4430(10)	90
**V (Å^3)**	3214.3(4)	3902.0(4)	1466.62(3)	2948.6(3)
Z	4	4	2	4
**ρ (g/cm^3)**	1.304	1.248	1.320	1.380
**μ (mm^{-1})**	0.699	0.587	6.247	0.764
**Data / restraints / parameters**	6351 / 0 / 346	7971 / 0 / 412	5183 / 0 / 311	6766 / 0 / 329
**$R1$, w$R2$ (I > 2σ(I))**	0.0466, 0.0777	0.0403, 0.0696	0.0249, 0.0594	0.0295, 0.0598
**$R1$, $wR2$ (all data)**	0.0882, 0.0871	0.0627, 0,0741	0.0273, 0.0605	0.0410, 0.0627
**GOF**	0.998	1.020	1.064	1.021
**Largest Diff. Peak, Hole (e.A^{-3})**	0.71, -0.64	0.46, -0.64	0.44, -0.64	0.44, -0.39

Table S4. Details of X-ray crystal structures.

Compound	$(^{tBu}PBP)Pd(CH_2CH_3)$	$(^{tBu}PBP)Pd(C_6H_5)$	$(^{tBu}PB^{Bn}P)_2Pd_2$	$(^{tBu}PB^{H}P)_2Pd_2$	$(^{tBu}PB^{All}P)_2Pd_2$
**CCDC**	2243696	2243700	2234701	2243702	2264960
**Identification Code**	007b-22021	007a-22028	007a-23009	mini2-22009	syn-23079
**Empirical Formula**	$C_{26}H_{49}BN_2P_2Pd$	$C_{30}H_{49}BN_2P_2Pd$	$C_{74}H_{114}B_2N_4P_4Pd_2$	$C_{60}H_{102}B_2N_4P_4Pd_2$	$C_{78}H_{122}B_2N_4P_4Pd_2$
**Wavelength (Å)**	1.54184	1.54184	1.54184	0.71073	1.54184
**Temperature (K)**	93(2)	93(2)	93(2)	93(2)	100(2)
**FW**	568.82	616.86	1417.99	1237.75	1474.09
**Crystal System**	monoclinic	monoclinic	monoclinic	triclinic	triclinic
**Space Group**	$P2_1/n$	$C2$/c	$P2_1/c$	$P\bar{1}$	$P\bar{1}$
a **(Å)**	10.6371(3)	36.5512(13)	24.5886(7)	10.1464(8)	9.9648(3)
b **(Å)**	19.9621(5)	8.0925(2)	11.3054(3)	11.2723(8)	13.3134(4)
c **(Å)**	14.4091(3)	21.4368(6)	29.5087(7)	15.0798(10)	14.9073(4)
**α (º)**	90	90	90	71.643(6)	85.337(2)
**β (º)**	109.290(3)	103.208(3)	114.397(3)	89.513(3)	79.407(3)
**γ (º)**	90	90	90	83.927(6)	89.959(2)
V **(Å³)**	2887.84(13)	6173.1(3)	7470.5(4)	1627.2(2)	1937.34(10)
Z	4	8	4	1	1
ρ **(g/cm³)**	1.308	1.327	1.261	1.263	1.263
μ **(mm⁻¹)**	6.332	5.971	5.002	0.689	4.841
**Data / restraints / parameters**	5101 / 0 / 302	5461 / 16 / 373	13189 / 0 / 800	5701 / 0 / 340	17232 / 0 / 419
***R*1, w*R*2 (I > 2σ(I))**	0.0787, 0.2066	0.0376, 0.0834	0.0789, 0.1878	0.0650, 0.1535	0.0428, 0.1124
***R*1, *wR*2 (all data)**	0.0861, 0.2222	0.0399, 0.0847	0.0834, 0.1899	0.0871, 0.1637	0.0482, 0.1160
**GOF**	1.055	1.096	1.176	0.994	1.048
**Largest Diff. Peak, Hole (e.A⁻³)**	0.52, -3.13	0.60, -0.76	2.09, -1.40	2.16, -1.71	0.96, -1.05

Table S4 Continued. Details of X-ray crystal structures.

SXIII. References

1. Harris, R. K.; Becker, E. D.; De Menezes, S. M.; Granger, P.; Hoffman, R. E.; Zilm, K. W. Further Conventions for NMR Shielding and Chemical Shifts (IUPAC Recommendations 2008). *Magn. Reson. Chem.* **2008**, *46*, 582-598.
2. Deziel, A. P.; Espinosa, M. R.; Pavlovic, L.; Charboneau, D. J.; Hazari, N.; Hopmann, K. H.; Mercado, B. Q. Ligand and Solvent Effects on CO_2 Insertion into Group 10 Metal Alkyl Bonds. *Chem. Sci.* **2022**, *13*, 2391-2404.
3. Ogawa, H.; Yamashita, M. Platinum Complexes Bearing a Boron-Based PBP Pincer Ligand: Synthesis, Structure, and Application as a Catalyst for Hydrosilylation of 1-Decene. *Dalton Trans.* **2013**, *42*, 625-629.
4. (a) Espinosa, M. R.; Ertem, M. Z.; Barakat, M.; Bruch, Q. J.; Deziel, A. P.; Elsby, M. R.; Hasanayn, F.; Hazari, N.; Miller, A. J. M.; Pecoraro, M. V.; Smith, A. M.; Smith, N. E. Correlating Thermodynamic and Kinetic Hydricities of Rhenium Hydrides. *J. Am. Chem. Soc.* **2022**, *144*, 17939-17954; (b) Johnson, M. T.; Johansson, R.; Kondrashov, M. V.; Steyl, G.; Ahlquist, M. S. G.; Roodt, A.; Wendt, O. F. Mechanisms of the CO_2 Insertion into (PCP) Palladium Allyl and Methyl σ-Bonds. A Kinetic and Computational Study. *Organometallics* **2010**, *29*, 3521-3529.
5. Frisch, M. J.; Trucks, G. W.; Schlegel, H. B.; Scuseria, G. E.; Robb, M. A.; Cheeseman, J. R.; Scalmani, G.; Barone, V.; Petersson, G. A.; Nakatsuji, H.; Li, X.; Caricato, M.; Marenich, A. V.; Bloino, J.; Janesko, B. G.; Gomperts, R.; Mennucci, B.; Hratchian, H. P.; Ortiz, J. V.; Izmaylov, A. F.; Sonnenberg, J. L.; Williams; Ding, F.; Lipparini, F.; Egidi, F.; Goings, J.; Peng, B.; Petrone, A.; Henderson, T.; Ranasinghe, D.; Zakrzewski, V. G.; Gao, J.; Rega, N.; Zheng, G.; Liang, W.; Hada, M.; Ehara, M.; Toyota, K.; Fukuda, R.; Hasegawa, J.; Ishida, M.; Nakajima, T.; Honda, Y.; Kitao, O.; Nakai, H.; Vreven, T.; Throssell, K.; Montgomery Jr., J. A.; Peralta, J. E.; Ogliaro, F.; Bearpark, M. J.; Heyd, J. J.; Brothers, E. N.; Kudin, K. N.; Staroverov, V. N.; Keith, T. A.; Kobayashi, R.; Normand, J.; Raghavachari, K.; Rendell, A. P.; Burant, J. C.; Iyengar, S. S.; Tomasi, J.; Cossi, M.; Millam, J. M.; Klene, M.; Adamo, C.; Cammi, R.; Ochterski, J. W.; Martin, R. L.; Morokuma, K.; Farkas, O.; Foresman, J. B.; Fox, D. J. Wallingford, CT, 2016.
6. (a) Perdew, J. P.; Burke, K.; Ernzerhof, M. Generalized Gradient Approximation Made Simple. *Phys. Rev. Lett.* **1996**, *77*, 3865-3868; (b) Perdew, J. P.; Burke, K.; Ernzerhof, M. Generalized Gradient Approximation Made Simple. *Phys. Rev. Lett.* **1997**, *78*, 1396-1396; (c) Adamo, C.; Barone, V. Toward Reliable Density Functional Methods Without Adjustable Parameters: The PBE0 Model. *J. Chem. Phys.* **1999**, *110*, 6158-6170.
7. (a) Grimme, S.; Ehrlich, S.; Goerigk, L. Effect of the Damping Function in Dispersion Corrected Density Functional Theory. *J. Comput. Chem.* **2011**, *32*, 1456-1465; (b) Grimme, S.; Antony, J.; Ehrlich, S.; Krieg, H. A Consistent and Accurate Ab Initio Parametrization of Density Functional Dispersion Correction (DFT-D) for the 94 Elements H-Pu. *J. Chem. Phys.* **2010**, *132*, 154104.
8. (a) Schuchardt, K. L.; Didier, B. T.; Elsethagen, T.; Sun, L.; Gurumoorthi, V.; Chase, J.; Li, J.; Windus, T. L. Basis Set Exchange: A Community Database for Computational Sciences. *J. Chem. Inf. Model.* **2007**, *47*, 1045-1052; (b) Pritchard, B. P.; Altarawy, D.; Didier, B.; Gibson, T. D.; Windus, T. L. New Basis Set Exchange: An Open, Up-to-Date Resource for the Molecular Sciences Community. *J. Chem. Inf. Model.* **2019**, *59*, 4814-4820.
9. Andrae, D.; Häußermann, U.; Dolg, M.; Stoll, H.; Preuß, H. Energy-Adjusted Ab Initio Pseudopotentials for the Second and Third Row Transition Elements. *Theor. Chim. Acta* **1990**, *77*, 123-141.
10. (a) Weigend, F.; Ahlrichs, R. Balanced Basis Sets of Split Valence, Triple Zeta Valence and Quadruple Zeta Valence Quality for H to Rn: Design and Assessment of Accuracy. *Phys. Chem. Chem. Phys.* **2005**, *7*, 3297-3305; (b) Schäfer, A.; Huber, C.; Ahlrichs, R. Fully Optimized Contracted Gaussian Basis Sets of Triple Zeta Valence Quality for Atoms Li to Kr. *J. Chem. Phys.* **1994**, *100*, 5829-5835; (c) Schäfer, A.; Horn, H.; Ahlrichs, R. Fully Optimized Contracted Gaussian Basis Sets for Atoms Li to Kr. *J. Chem. Phys.* **1992**, *97*, 2571-2577; (d) Weigend, F. Accurate Coulomb-Fitting Basis Sets for H to Rn. *Phys. Chem. Chem. Phys.* **2006**, *8*, 1057-1065.
11. Scalmani, G.; Frisch, M. J. Continuous Surface Charge Polarizable Continuum Models of Solvation. I. General Formalism. *J. Chem. Phys.* **2010**, *132*, 114110.

12. Chai, J.-D.; Head-Gordon, M. Long-Range Corrected Hybrid Density Functionals with Damped Atom–Atom Dispersion Corrections. *Phys. Chem. Chem. Phys.* **2008**, *10*, 6615-6620.
13. Tao, J.; Perdew, J. P.; Staroverov, V. N.; Scuseria, G. E. Climbing the Density Functional Ladder: Nonempirical Meta--Generalized Gradient Approximation Designed for Molecules and Solids. *Phys. Rev. Lett.* **2003**, *91*, 146401.
14. Sheldrick, G. M. A Short History of SHELX. *Acta Crystallogr. A* **2008**, *64*, 112-122.
15. Spek, A. Single-Crystal Structure Validation with the Program PLATON. *J. Appl. Crystallogr.* **2003**, *36*, 7-13.
